# The New Paradigm of Network Medicine to Analyze Breast Cancer Phenotypes

**DOI:** 10.3390/ijms21186690

**Published:** 2020-09-12

**Authors:** Anna Maria Grimaldi, Federica Conte, Katia Pane, Giulia Fiscon, Peppino Mirabelli, Simona Baselice, Rosa Giannatiempo, Francesco Messina, Monica Franzese, Marco Salvatore, Paola Paci, Mariarosaria Incoronato

**Affiliations:** 1 IRCCS SDN, Via Emanuele Gianturco 113, 80143 Naples, Italy; annamaria.grimaldi@synlab.it (A.M.G.); katia.pane@synlab.it (K.P.); peppino.mirabelli@synlab.it (P.M.); simona.baselice@synlab.it (S.B.); monica.franzese@synlab.it (M.F.); direzionescientifica@sdn-napoli.it (M.S.); 2 Institute for Systems Analysis and Computer Science “Antonio Ruberti”, National Research Council, 00185 Rome, Italy; federica.conte@iasi.cnr.it (F.C.); giulia.fiscon@iasi.cnr.it (G.F.); 3 Ospedale Evangelico Betania, Via Argine 604, 80147 Naples, Italy; giannatiemporosa@libero.it (R.G.); messina52@alice.it (F.M.); 4 Department of Computer, Control and Management Engineering, Sapienza University of Rome, 00185 Rome, Italy

**Keywords:** breast cancer, Network Medicine, TCGA, Network-based algorithm, Disease modules, Switch genes and Interactome

## Abstract

Breast cancer (BC) is a heterogeneous and complex disease as witnessed by the existence of different subtypes and clinical characteristics that poses significant challenges in disease management. The complexity of this tumor may rely on the highly interconnected nature of the various biological processes as stated by the new paradigm of Network Medicine. We explored The Cancer Genome Atlas (TCGA)-BRCA data set, by applying the network-based algorithm named SWItch Miner, and mapping the findings on the human interactome to capture the molecular interconnections associated with the disease modules. To characterize BC phenotypes, we constructed protein–protein interaction modules based on “hub genes”, called switch genes, both common and specific to the four tumor subtypes. Transcriptomic profiles of patients were stratified according to both clinical (immunohistochemistry) and genetic (PAM50) classifications. 266 and 372 switch genes were identified from immunohistochemistry and PAM50 classifications, respectively. Moreover, the identified switch genes were functionally characterized to select an interconnected pathway of disease genes. By intersecting the common switch genes of the two classifications, we selected a unique signature of 28 disease genes that were BC subtype-independent and classification subtype-independent. Data were validated both in vitro (10 BC cell lines) and ex vivo (66 BC tissues) experiments. Results showed that four of these hub proteins (AURKA, CDC45, ESPL1, and RAD54L) were over-expressed in all tumor subtypes. Moreover, the inhibition of one of the identified switch genes (AURKA) similarly affected all BC subtypes. In conclusion, using a network-based approach, we identified a common BC disease module which might reflect its pathological signature, suggesting a new vision to face with the disease heterogeneity.

## 1. Introduction

Breast cancer (BC) impacts 2.1 million women each year, representing the most frequent cancer among females, such that in 2018 approximately 15% of all cancer deaths among women were for BC (627,000 women) [1]. BC is a heterogeneous pathology as witnessed by the existence of different subtypes with distinct morphologies and clinical implications [2]. These subtypes are usually defined by using immunohistochemical (IHC) [3] and genetic (PAM50) [4,5] classifications. Although the information provided by PAM50 includes a more complete molecular profile than IHC, it is not readily available for all patients in many countries being too expensive. Consequently, the use of IHC stratification is routinely adopted in the clinical practice for prognostication and treatment decision making. Despite the remarkable increase in the depth of understanding of BC, the disease is still a major public health problem worldwide and poses significant open challenges. This failure may be attributed to continuing adherence to the classical hypothesis (one gene, one drug, one disease) of the reductionist paradigm that has driven medical diagnosis in the modern era. Another important factor limiting the development of effective therapeutic strategies is the canonical disease classification that is largely based on clinicopathological evidence and often categorized according to the organ that the disease primarily affects, neglecting the interconnected nature of many diseases. To overcome these shortcomings, very promising insights come from the newly emerging field of Network Medicine, which applies tools and concepts from network theory to elucidate the relation between perturbations on the molecular-level and phenotypic disease manifestations. In Network Medicine construct diseases are rarely caused by a single gene mutation, but more typically by the deregulation of a network of genes interconnected each other. Thus, in this revolutionary vision of the human diseases, the interactome, i.e., the integrated network of all physical interactions within the cell, can be interpreted as a map and diseases as local perturbations. Network Medicine research promises to provide a more global understanding of how the specific interactome neighborhood is perturbed in a certain disease, identify pathways and key components to be targeted in clinical interventions and reveal common molecular mechanisms between seemingly unrelated diseases [6,7]. 

Searching for molecular signatures underlying the different subtypes of BC, here we exploited one of the most promising network-medicine-based algorithms, named SWItch Miner (SWIM). SWIM methodology builds upon the structural properties of gene co-expression networks (GENs) to mine key genes (called switch genes) likely associated with drastic physiological changes in many biological settings. Up to now, the relevance of switch genes related to an observed phenotype has been widely assessed through several applications ranging from grapevine berry maturation to complex diseases development [8,9,10,11,12,13]. 

Specifically, for each BC subtypes of both subtype classifications (IHC and PAM50), the transcriptomic profiling of The Cancer Genome Atlas (TCGA) breast collection [14] was analyzed by computational and functional genomic approaches, to identify “switch” genes Shared among subtypes (S) and Specific for each Subtype (SS). Moreover, data obtained from the two classifications (IHC and PAM50) were intercrossed each other to identify a network of switch genes common to all subtypes and both classifications. Then, to validate the in silico data, in vitro and ex vivo experiments were performed, by using BC model cell lines and tissue specimens, as well as functional studies. In this scenario, the results of the present study could effectively transform genomic data in actionable knowledge to improve our understanding of this pathology and the management of the BC patient.

## 2. Results

### 2.1. Overlapping BC Subtype PPI-Based Modules by Switch Genes

**Figure 1:**
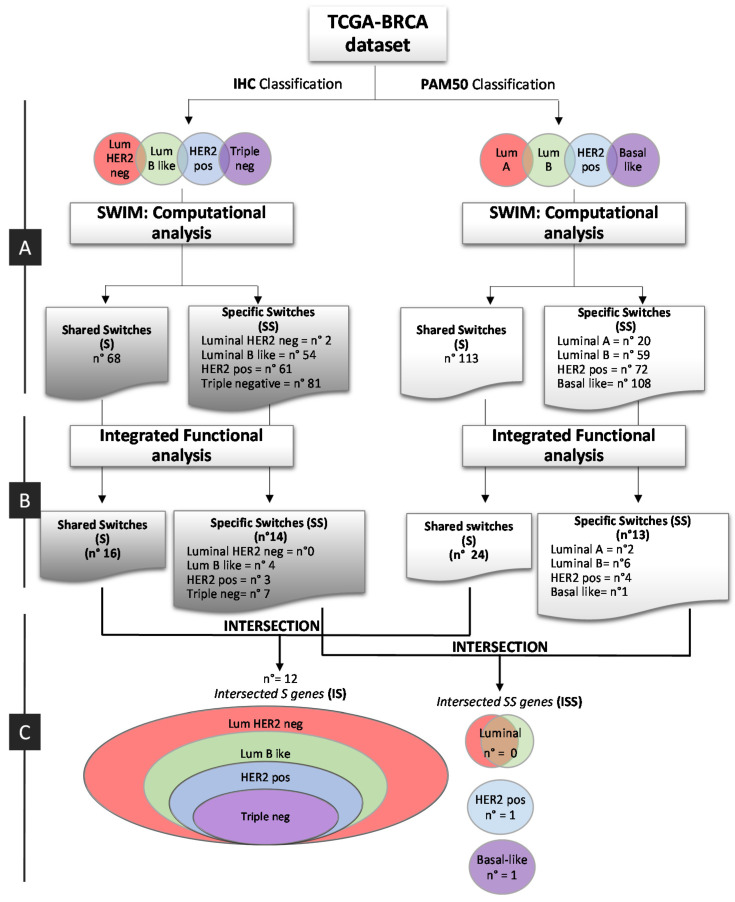
Bioinformatic workflow. Study design for the identification of breast cancer switch genes from IHC and PAM50 subtype classification. (**A**) Computational analysis; (**B**) Integrated functional analysis; (**C**) Integration of IHC and PAM50 Key Regulatory Switches. pos: positive; neg: negative.

**Figure 2:**
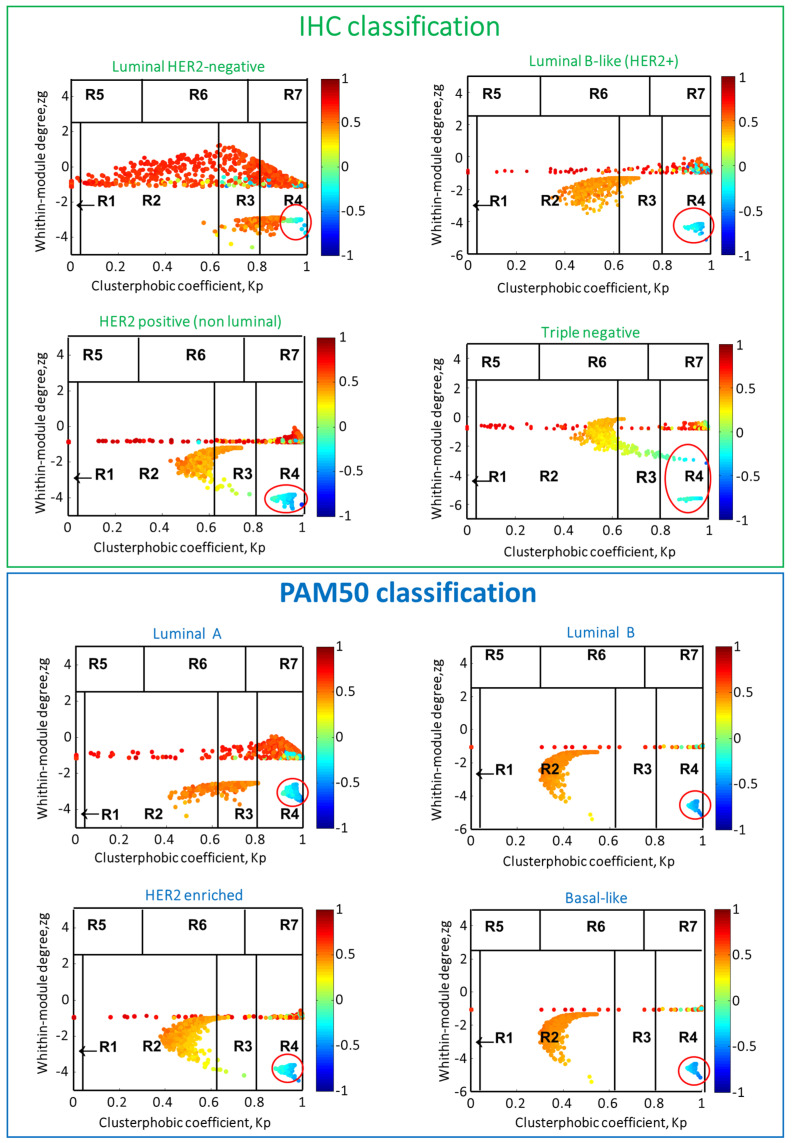
Heat cartography maps for each breast subtype. The cartographic representation of the correlation network of each analyzed breast subtype is reported. The x and y axes correspond to the within-module degree zg and the clusterphobic coefficient Kp, respectively. The within-module degree zg measures how “well-connected” each node is to other nodes in its own cluster, and the clusterphobic coefficient Kp measures the “fear” of being confined in a cluster. High zg values correspond to nodes that are hubs within their community, while high values of Kp identify nodes that interact mainly outside their community, i.e., with many more external than internal links [8]. Dots correspond to nodes in the networks colored according to the value of the Average Pearson Correlation Coefficients (APCC) between its expression profile and that of its nearest neighbors. Switch genes correspond to nodes colored in blue and falling in R4 region (highlighted with a red circle).

The whole bioinformatics workflow is reported in Figure 1. We used TCGA BC gene expression data and the SWIM methodology [8] to identify switch genes, for each BC subtype (Table 1, Figure 2, and Figure S1). Please note that we chose SWIM running parameters to obtain a manageable correlation network for each BC subtype (Table 1).

We categorized TCGA BC patients in the genetic and clinical molecular subtype (Table 2 and Material and Methods), according to PAM50 and IHC classification. Then, for both classifications (IHC and PAM50), we determined switches shared among all subtypes (S) and those subtype-specific (SS). We identified a total of 266-IHC switches of which 68-S (Figure S2A), and 372-PAM50 switches of which 113-S (Figure S2B). 

**Figure 3:**
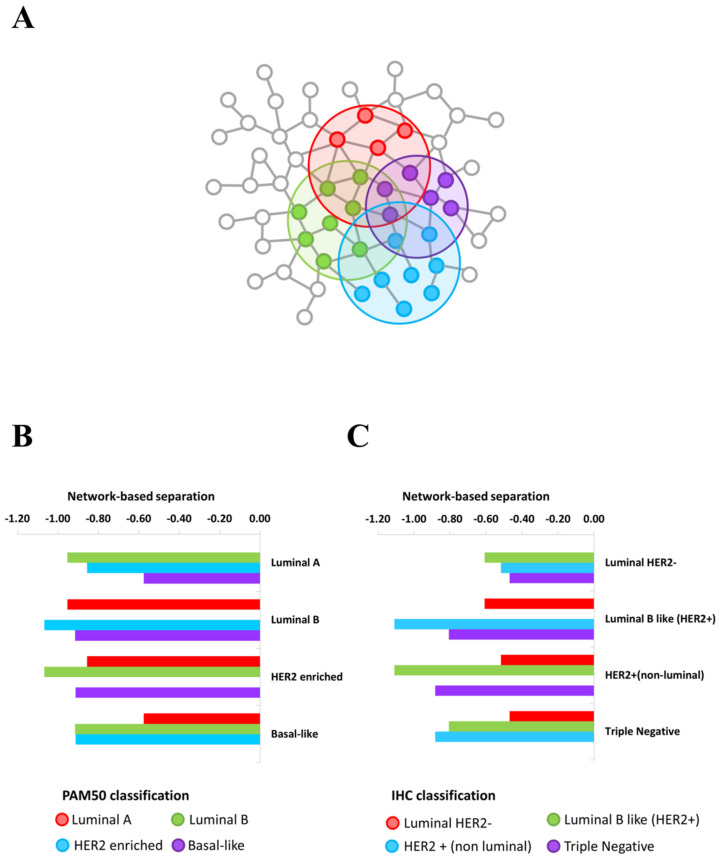
Overlapping BC subtype PPI-based modules by switch genes. (**A**) Schematic representation of the overlapping modules identified by BC subtypes switch genes in the human interactome. (**B**,**C**) Bar plot reporting the values of the network-based separation measure computed between each pair of switch genes’ modules of PAM50 classification (**B**) and IHC classification (**C**).

The switch genes identified for each BC subtypes of each classification showed an intriguing common pattern of molecular co-abundance (Table 1 and Figure S3), which strongly point at them as a small core of functionally coordinated genes that cooperate together to potentially orchestrate the observed pathobiological phenotype transition. This observation suggested how they might be part of complexes, influence each other, or be part of the same pathways or mechanisms, expecting to localize in the same interactome neighborhood [15]. Thus, we investigated whether BC subtype switch genes tend to agglomerate in connected subgraphs in the human interactome. Thus, for each BC subtype, we verified that the largest connected component of switch genes showed the size and several interactions greater than expected by chance, and thus we demonstrated that switch genes constituted statistically significant BC subtype modules in the human interactome. Furthermore, we verified whether these modules overlapped each other by computing the network-based separation measure between each BC subtype switch genes module and by applying a degree-preserving randomization procedure to assess a statistical significance. We found statistically significant (*p*-value < 0.05) negative separation values between each pair of BC subtype, meaning that the switch genes modules directly overlap themselves (Figure 3 and Table S1). 

### 2.2. Integration and Prediction of IHC and PAM50 Key Regulatory Switches

**Figure 4:**
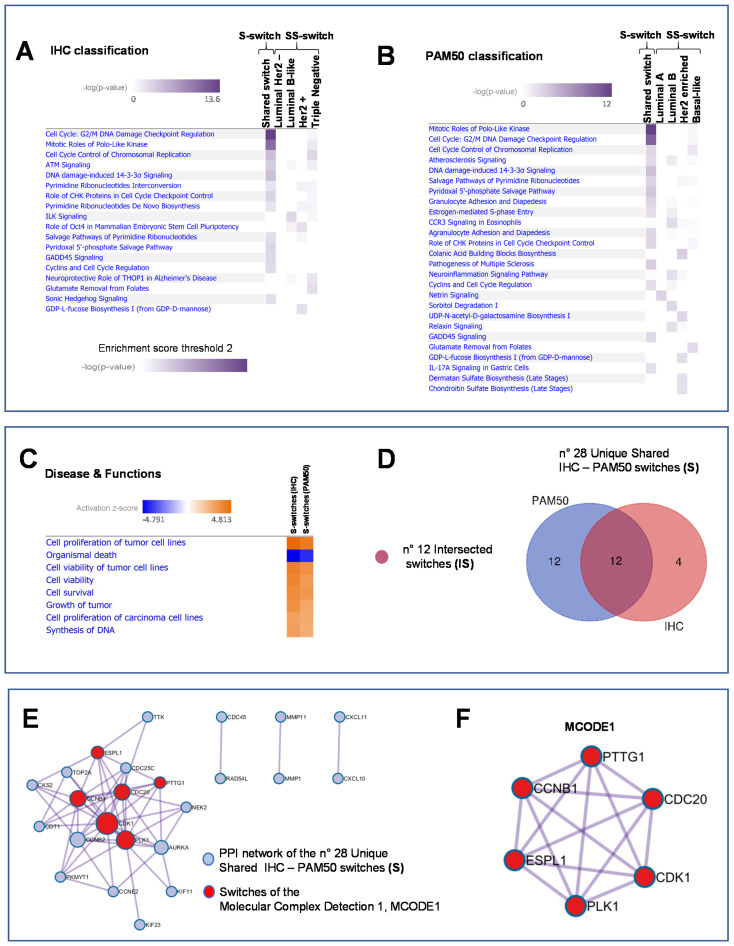
Integration and prediction of IHC and PAM50 key regulatory switches. (**A**) and (**B**) Heatmaps of the enriched pathways for IHC and PAM50 classification, respectively. Enrichment score (Fisher’s Exact right-tailed test, *p*-value < 0.01). (**C**) Prediction of inhibited (blue squares) and activated (orange squares) functions of shared IHC and PAM50 switches sorted by activation Z-score (absolute value 3). (**D**) Venn diagram defining the Intersected Shared (IS) switches of shared IHC and PAM50 switches. (**E**) Protein–protein interaction (PPI) network of the 28 Unique Shared IHC–PAM50 switches resulted in 24 PPI network molecules. (**F**) MCODE1 represents a densely connected protein complex (6 in red out of 24) of the PPI network by the Molecular Complex Detection algorithm.

According to the workflow depicted in Figure 1B left and right, we integrated the 266-IHC and 372-PAM50 switch genes into regulatory processes by the Ingenuity Pathways Analysis (IPA) software (IPA, QIAGEN, Inc. Redwood City, CA, USA [16]). Heatmaps show the enriched biological processes scores of IHC and PAM50 S-switches and SS switches, respectively (Figure 4A and B, Table S2 and Table S3, respectively). Since pathways selectively enriched may elucidate critical disease mechanisms, we defined for IHC 16 S-switches and 14 SS switches as members of the top enriched pathways (Figure 4A, Table S4 and Table S5, respectively), while for PAM50, 24 S-switches and 13 SS switches (Figure 4B, Table S6 and Table S7, respectively). Notable, the highest enriched pathways related to cell-cycle regulation and mitotic signaling pathways were found in association with S-switches both in IHC and in PAM50 classifications, as well as S-switches enriched many biological pathways common to IHC and PAM50 classifications (Figure 4A and B and Table S4 and Table S6, respectively). We furtherly explored the disease functions underlying IHC and PAM50 S-switches expression changes, by IPA activation Z-score (Figure 4C and Methods). IHC and PAM50 S-switches inhibited mechanisms related to “Organismal death” (Figure 4C blue squares) and activated “Cell proliferation of tumor cell lines”, “Cell viability” and “Cell survival” mechanisms (Figure 4C, orange squares). Overall, in silico analyses indicated that S-switches may promote uncontrolled proliferation program due to their aberrant expression. Thus, being the identified S-switches shared in all subtypes for each classification, we hypothesized that these genes might be key disease phenotype molecules independent not only from tumor subtypes but even from classifications. To assess this hypothesis, we carried out an intersection (I) between S-switches of IHC and PAM50 classifications (Figure 1C left) to find a common switches signature. We computed overall 28 unique IHC – PAM50 S-switches (Figure 4D); all of them resulted up-regulated in TCGA breast tumor samples compared with normal samples; 22 out of 28 switches are “oncogenes” annotated in CancerMine database [17] (Table S8); 12 out of 28 switches are common to both classifications and named “intersected S genes” (IS; Figure 1C left and Figure 4D). We also evaluated the intersection of SS switches between IHC and PAM50 (Figure 1C right and Figure S4), and named “intersected SS genes” (ISS). We found no ISS for luminal subtypes intersection (Figure S4A), 1 ISS such as the tissue-specific transplantation antigen P35B (TSTA3, ENSG00000278243, Figure S4B) for HER2 positive subtype, and 1 ISS such as the gamma-glutamyl hydrolase (GGH, ENSG00000137563, Figure S4C) for Basal subtype.

We focused on the 28 unique IHC-PAM50 S-switches to investigate their physical interactions. To this aim, we performed interactome analysis using the Molecular Complex Detection (MCODE) algorithm. In the protein–protein interaction (PPI) network (Figure 4E), we distinguished a densely connected protein complex that constituted the MCODE1 (Figure 4E in red and Figure 4F) and three pairs of neighborhood molecules. As expected, this result was predominantly confirmed by applying the MCODE algorithm even to the 12 IS switches (Figure S5).

### 2.3. In Vitro and Ex Vivo Validation of Switch Genes and Functional Studies

In summary, SWIM tool and in silico functional analyses led us to isolate and analyze a small number of regulatory genes (28 unique IHC-PAM50 S-switches) starting from thousands of them. To validate the results of the computational analyses, in vitro and ex vivo experiments have been performed choosing: (i) those genes densely connected (MCODE1 Figure 4F; *PTTG1, CCNB1, ESPL1, PLK1, CDK1* and *CDC20*); (ii) resting genes included in the Figure S5C (MCODE1; *AURKA, NEK2* and *CCNB2*); (iii) a pair of neighborhood molecules conserved in both PPI network (Figure 4E and Figure S5B; *CDC45* and *RAD54L*). Thus, we integrated the biological meaning of both PPI networks (Figure 4E and Figure S5) for downstream analysis.

The relative expression of this group of 11 genes (Table 3) was evaluated by real-time PCR on different BC cell lines.

**Figure 5:**
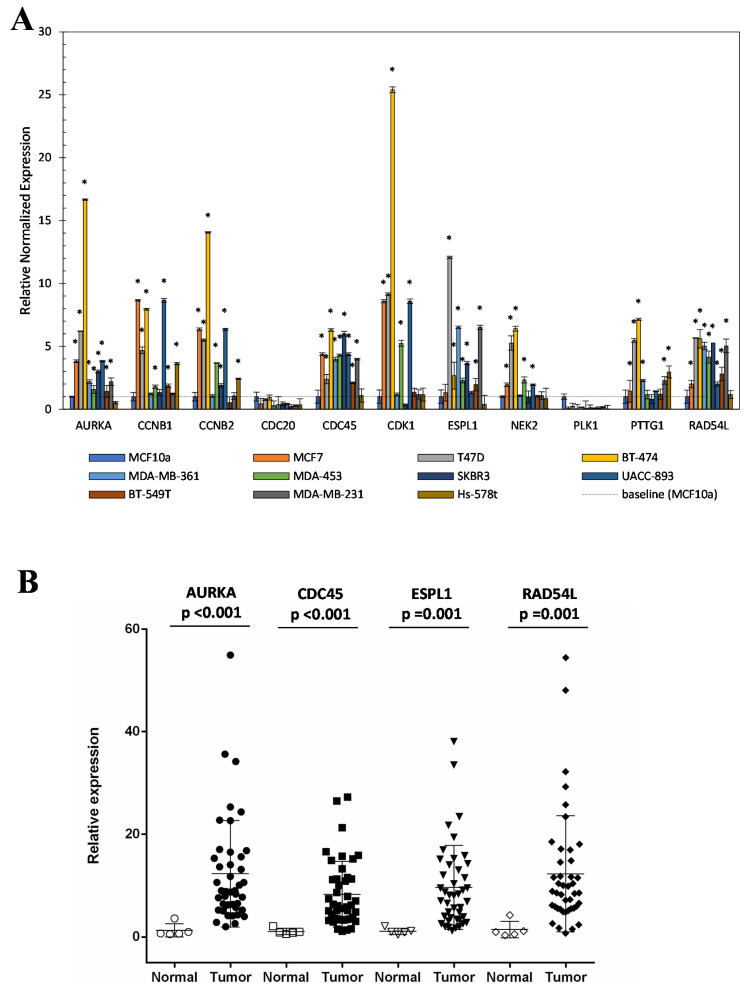
Relative expression of switch genes in BC cell lines and tissue. (**A**) Switch gene expression was evaluated in BC cell lines vs control cell by real-time PCR. (**B**) AURKA, CDC45, ESPL1, and RAD54L genes were evaluated in BC tissue using TissueScan qPCR Arrays. *p < 0.05 was considered statistically significant.

Total RNA was extracted from the following cell lines: T-47D and MCF-7 as representative of Luminal A subtype; BT-474 and MDA-MB-361 as representative of Luminal B subtype; MDA-MD-453, SKBR3 and UACC-893 as representative of HER2 positive subtype; and MDA-MB-231, BT-549, and Hs-578T as representative of Triple-negative subtype. As a negative control, MCF10a cell line was used. The results reported in Figure 5A showed that except for CDC20 and PLK1 genes, mRNA levels of identified switches increased in most tested BC cell lines compared to the negative control (MCF10a).

Figure S6 shows the expression of these switches in cell lines grouped by tumor subtype. As shown in Figure 5A, except for Hs-578T cell line, the mRNA levels of AURKA, CDC45, ESPL1, and RAD54L proteins were high in all analyzed BC cell lines. To confirm this result, the mRNA expression of these four proteins was also evaluated in breast tissue specimens of unaffected and affected subjects using TissueScan qPCR Arrays (https://cdn.origene.com/assets/documents/tissuescan/bcrt502.xls). As reported in Figure 5B, mRNA of all four switch genes was significantly increased in BC tissues compared to normal ones (AURKA *p*-value < 0.001, ESPL1 *p*-value = 0.001, CDC45 *p*-value < 0.001 and RAD54L *p*-value = 0.001). Using arrays data, and grouping Luminal A with Luminal B cases as “luminal” and Her2+ with Triple-negative as “not luminal” subtypes, we evaluated the distribution of the four switches among two subtypes groups. As expected, we found that their distribution among BC subtypes was not statistically significant (Figure S7A–D). These results indicated that the increased mRNA amount of these molecules in BC cell lines and tissues was independent of tumor subtype, so it was not influenced by the heterogeneity of this pathology. Their distribution was also evaluated among grade and staging of disease and we found that it was independent by cellular differentiation (Grade) (Figure S8A–D) but, except for CDC45, associated with the staging of the disease (Figure S9A–D). Taken together, these results suggested that a protein network including AURKA, RAD54L, CDC45, and ESPL1 proteins, key molecules of pathways deregulated in cancer, could represent a common regulatory signature to all BC subtypes.

**Figure 6:**
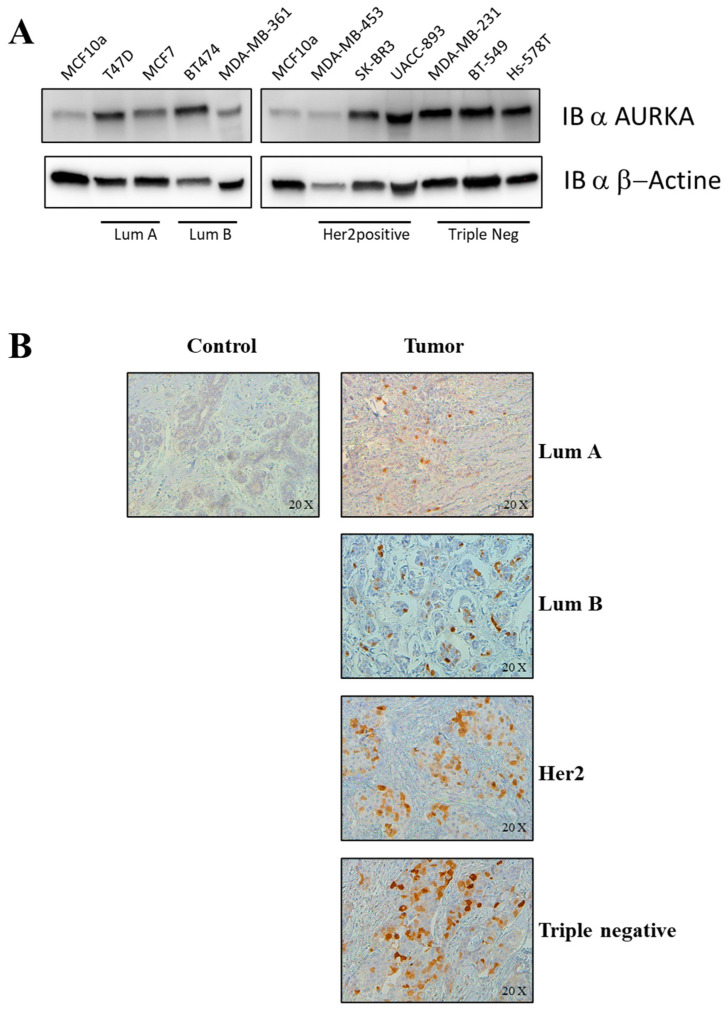
AURKA expression in vitro and ex vivo. (**A**) AURKA was examined by immunoblotting in T47D, MCF7 (Lum A), BT474, MDA-MB-361 (Lum B), MDA-MB-453, SK-BR3, UACC-893 (Her2), BT-549, Hs-578T (Triple Negative) and MCF10a (Control). Β-actin served as a loading control. (**B**) Immunohistochemical analysis of AURKA was performed in surgical specimens of BC. The panel shows a control tissue with no AURKA-positive cells, and four tumoral tissues, one for each subtype (Lum A, Lum B, Her2, Triple-negative) with AURKA-positive cells. Magnification 20X.

Functional studies have been performed to evaluate if the inhibition of at least one of the identified switch genes could affect all BC subtypes. To this aim, we decided to choose AURKA protein as a representative switch among those identified because this kinase: (i) regulates cell division and cell-cycle progression; (ii) is deregulated in many human cancers; (iii) is suggested as a priority pharmaceutical target for the treatment of cancers [18]; and (iv) are known and validated its specific inhibitors [19,20,21]. As reported in Figure 6A, AURKA protein was markedly over-expressed in all BC cell lines analyzed compared to the negative control (MCF10a cell line). Furthermore, AURKA protein expression was evaluated in breast tissues specimens of affected and unaffected subjects by IHC experiments. As reported in Figure 6B, AURKA protein was over-expressed in all BC tissues compared to normal tissues. Taken together the results on cells and tissues correlated with those obtained analyzing AURKA mRNA expression. There are currently many commercially available AURKA inhibitors, several involved in many clinical trials, and one of these is MLN8237 (alisertib). AURKA is primarily regulated by phosphorylation in a cell-cycle-dependent manner, occurring on residue Thr288. The binding of alisertib at the pocket site of AURKA inhibits its autophosphorylation at Thr288 and so its catalytic activity [22]. AURKA is critical for proper formation of the mitotic spindle, and as a key cell-cycle regulator, this kinase regulates G2/M transition.

**Figure 7:**
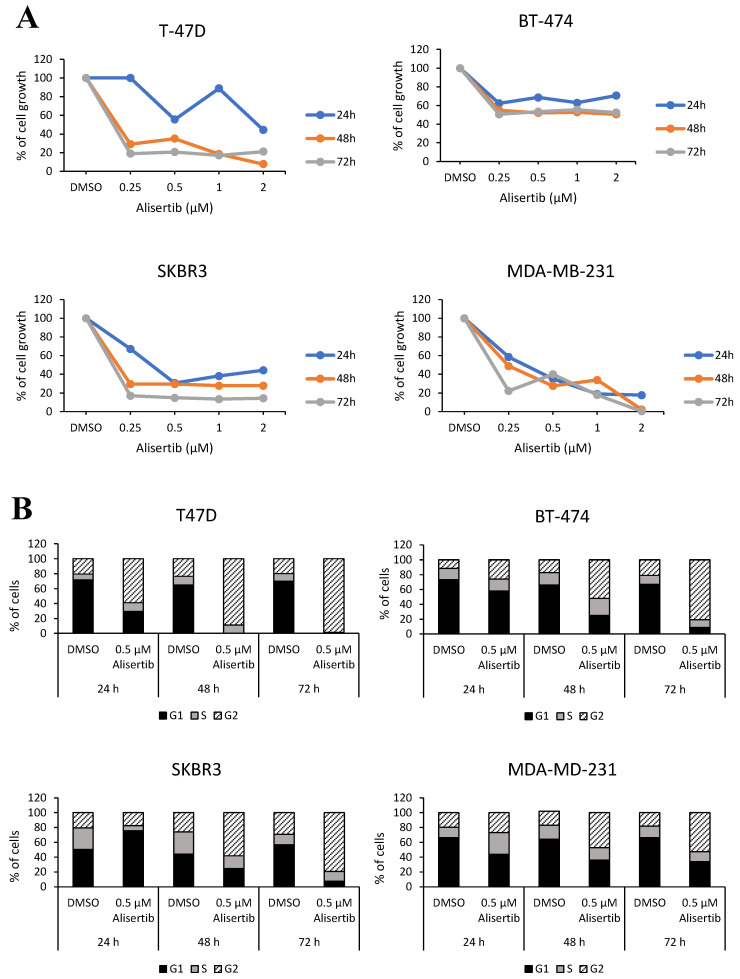
Effect of Alisertib treatment on BC cell lines. (**A**) Alisertib effects were assessed on cell growth: T-47D, BT-474, SKBR3, and MDA-MB-231 cell lines were treated with 0.25, 0.5, 1 and 2 µM of the drug for 24, 48, and 72 h and DMSO treatment was used as a negative control. (**B**) Alisertib effects were assessed on cell cycle: T-47D, BT-474, SKBR3, and MDA-MB-231 cell lines were treated with 0.5 µM of the drug for 24, 48, and 72 h and DMSO treatment was used as a negative control.

To evaluate its inhibition on cell viability, T47D, BT-474, SKBR3, and MDA-MB231 cell lines, each representing one BC subtype, were treated with alisertib. Cell viability assay indicated 0.5 µM as the minimum dose of alisertib able to inhibit the cell growth of all cell lines up to three days of treatment (Figure 7A). We further evaluated the effect of alisertib treatment on cell-cycle arrest. Compared to untreated (DMSO) cells, the percentage of all the indicated cell lines in G2/M phase clearly increased in a time-dependent manner reaching the maximum cell-cycle arrest after 72 h of treatment (Figure 7B). Taken together these results indicated that the inhibition of AURKA slowed cell division similarly in all four BC subtypes, suggesting that this hub gene could represent an important regulatory molecule that does not seem to suffer the effect of the pathology’s heterogeneity.

## 3. Discussion

BC presents a high inter-tumor and intra-tumor heterogeneity that complicates its management. The new paradigm of Network Medicine offers a very promising landscape to capture the human disease complexity to better understand the common disease modules underlying seemly various phenotypes as in the case of BC.

In this study, to identify putative disease genes (switch genes) shared by BC subtypes, we applied network-based approach (SWIM algorithm) on TCGA-BRCA patient collection stratified according to both clinical (IHC) and genetic (PAM50) classifications. We demonstrated those switch genes associated with each analyzed BC subtypes formed disease modules in the human interactome and that these modules showed a considerable and statistically significant molecular-level overlap each other. These findings support the idea that the BC subtypes, although different from a clinicopathological point of view, may have mechanistic links based on hidden common pathomechanisms.

Based on our results, we could speculate that a protein network of 28 switches could be a signature of the BC phenotype independently from both tumor subtype and the type of classification. Among the 28 switches, we found that 11 of them were functionally connected in a very strong way (AURKA, CCNB1, CCNB2, CDC20, CDC45, CDK1, ESPL1, NEK2, PLK1, PTTG1, and RAD54L). To validate these computational results, we performed in vitro analyses using 10 BC cell lines, each of them subtype-specific, and MCF10a cell line as a negative control. In the same way, ex vivo analyses were performed on tissue specimens of 48 affected and unaffected subjects non-TCGA dependent. We found a disease module of four switches (AURKA, CDC45, ESPL1 and RAD54L) that was deregulated in all BC affected patients, beyond subtype classification. These four switches are disease genes involved in disease-associated pathways. AURKA protein plays a role in tumor development and progression of various cancers including BC [23,24,25,26,27,28]. CDC45 is an essential DNA replication factor [29]. It is required for DNA synthesis during genome duplication, as a component of the Cdc45-MCM-GINS (CMG) helicase [30] and implicated in diverse human cancers [31,32,33] including BC [34]. ESPL1 is involved in sister chromatids separation during anaphase and its oncogenic activity was found in BC [35,36] and endometrial cancer [37]. RAD54L was shown to play an important role in homologous recombination related to repair or DNA double-strand breaks and was found associated with progression and prognosis in diverse human cancers [38,39] and correlated with local recurrence and survival in BC [40].

To verify if the inhibition, at least of one of these hub genes, could affect all BC subtypes we performed functional studies choosing AURKA as a reference target switch for its known involvements in cancer development [18]. Before that, we confirmed deregulation of AURKA protein expression both in BC cell lines and in additional 27 tissues specimens of enrolled subjects. Functional studies were performed using a specific AURKA inhibitor named MLN8054 that works as an ATP-competitive molecule. The results obtained performing cell viability assays and cell-cycle analyses, treating cells with a specific AURKA’s inhibitor (alisertib), demonstrated that its inhibition reduced growth of cell lines tested similarly in all four cell subtypes.

However, this study has several limitations: (1) The SWIM methodology exploited in this study to identify BC-causing genes leverages phenotype-specific gene expression data in the network construction by calculating correlations between the expression profiles of each gene pairs. Even though “correlation is not causation”, co-expressed genes are functionally coordinated in response to an external stimulus meaning that they may be part of the same complexes or pathways, may influence each other or may be influenced by the same mechanisms; (2) despite we carried out validation on internal cohort, the sample size used for IHC and real-time experiments could be a limit. Certainly, data assessment on a larger cohort may considerably improve the robustness of these results. Nevertheless, our experimental results, using BC tissue arrays and tissues specimens have clearly shown that the selected switches are up-regulated in all four BC subtypes in agreement with in silico findings.

## 4. Materials and Methods

### 4.1. Dataset

Tumor and normal samples expression data from high-throughput RNA-sequencing and clinical metadata for breast invasive carcinoma were downloaded from TCGA data portal [14,41]. High-throughput RNA-sequencing data correspond to level 3 data (i.e., normalized expression data) given in terms of FPKM (i.e., fragments per kilobase of exon per million fragments mapped) and include 1182 samples (1069 tumor samples, 113 normal samples). Clinical metadata was available for a total of 1081 patients. Male samples, as well as samples undergoing a neoadjuvant treatment, were removed from the cohort under study.

### 4.2. Subtype Stratification

BC subtypes have been determined by using two classifications: intrinsic subtype, based on gene expression PAM50 [4,5] and clinical subtype based on immunochemistry (IHC) [3]. The TCGA-BRCA includes general pathologic and prognostic information and genetic subtypes identified by PAM50. Specifically, starting from 1081 BC patients, we selected 887 subjects as eligible for defining IHC subtypes and 502 subjects for which PAM50 subtypes were provided. The accurate definition of IHC subtypes requires both clinical data of immunoprofile (ER, PR and HER2 status) and Ki67 index (Table 2, first column), but TCGA-BRCA database lacks Ki67 index, needed to discriminate “luminal A-like” from “luminal B-like (HER2 negative)” subtypes. To overcome this limit, providing an IHC clinical stratification for the TCGA-BRCA dataset, samples ER positive, PR positive and HER2 negative were included in a modified subtype named “Luminal HER2 negative” (Table 2, second column), as a result of “luminal A-like” and “luminal B-like (HER2 negative)” subtypes grouping. “Luminal B-like (HER2 positive)”, “HER2 positive” and “Triple-negative” subtypes remained unchanged (Table 2). According to our processing, for each patient extracted from the TCGA database, the IHC subtype has been determined and reported in Table S9.

### 4.3. Gene Expression Data Analysis

To identify switch genes associated with the transition between normal condition and each breast subtype of both classifications, we exploited the software SWItch Miner (SWIM) [8]. A fully comprehensive description of SWIM algorithm is provided in [8]. SWIM was complemented with a statistical analysis to address the batch effect due to the age and to other unknown surrogate variables. In particular, a linear model was used to fit the expression data for each gene (EXP) to detect the association with the variable of interest representing the tumor/control condition (BC) by using the least squares regression model (EXP ~ BC + age + surrogate variables).

### 4.4. Network-Based Separation Measure

To test if the switch genes modules identified for each BC subtype overlap and thus if they are located in the same neighborhood of the human interactome network, we exploited the *network-based separation* measure, which quantifies the modules’ overlap as follows [42]:

$$s\left(A,B\right)=\langle d_{\mathrm{AB}}\rangle -\frac{\langle d_{\mathrm{AA}}\rangle +\langle d_{\mathrm{BB}}\rangle }{2}$$

where 〈d(A, B)〉 is defined as:

$$\langle d\left(A,\;B\right)\rangle =\frac{1}{\left|A\right|+\left|B\right|}\left[\underset{a\epsilon A}{\sum }\underset{b\epsilon B}{\min\;}d(a,b)+\underset{b\epsilon B}{\sum }\underset{a\epsilon A}{\min\;}d(b,a)\right]$$

and d(a, b) is the shortest distance between switch gene a of subtype module A and switch gene b of subtype module B. A positive separation value signals a topological separation between the two subtype modules in the human interactome (i.e., the two modules are located in well-separated neighborhoods), whereas a negative separation value signals a topological overlap (i.e., the two modules are located in the same neighborhood). To complement the separation measure with a statistical significance, we built a reference distribution corresponding to the expected distance between two randomly selected groups of proteins with the same size and degree distribution of the original two lists of switch genes in the human interactome. To build the reference distance distribution, we repeated the random section 1000 times. Thus, we used the mean and the standard deviation of the reference distribution to Z-score normalize the module separation of the two original lists of switch genes. Finally, we calculated the *p*-value for the given z statistic. A *p*-value < 0.05 indicates that the module separation in the human interactome of the two lists of switch genes is greater or lower than expected by chance. The human interactome exploited for this analysis was retrieved from [43].

### 4.5. Integrated Analyses for Identifying Key Regulatory Switches

To identify IHC and PAM50 key regulatory switches, we followed a step-by-step integrated functional analysis, using the software Ingenuity Pathway Analysis (IPA, QIAGEN, Inc. Redwood City, CA. Release 2019) [16], CancerMine database (accessed on April 2019, Vancouver, Canada) [17], and user-interface Metascape (accessed on 30 April 2019, CA, USA) [44]. According to the workflow step 1B, we uploaded shared switch genes (S-switch) and BC Subtype-Specific Switch genes (SS-switch) for each classification (IHC and PAM50) to perform comparison analyses. IPA canonical pathways most statistically significant enriched, for each classification, were filtered based on enrichment score ≥ 2 (Fisher’s Exact right-tailed test) [16]. The reference set for *p*-value calculations was Ingenuity Knowledge Base with direct and indirect relationships and experimentally observed settings for confidence. Moreover, we made predictions of disease functions using information about the direction of gene regulation (DEGs) of Shared Switches for each classification to compute the activation Z-score. This is achieved by assessing the consistency of the pattern match between the up/down gene-regulation pattern and the activation/inhibition pattern given by the network relative to a random pattern [16]. We sorted disease function predictions by Z-score with threshold 3 (absolute value). Moreover, we evaluated the Intersected SS genes (ISS) for luminal subtype IHC and PAM50 (Figure 1C right), merging all luminal subtypes in one group, since IHC and PAM50 differ each other for luminal subtypes stratification (see Table 2). Moreover, we investigated for 22 out of the 28 unique Shared switches between IHC and PAM50 classification, literature annotations as driver, oncogenes or tumor suppressor using CancerMine database [17]. To perform interactome analysis for the IHC - PAM50 28 unique Shared switches and protein–protein interaction network we used the user-interface Metascape with default settings as suggested by [44]. Protein–protein interaction network was exploited to investigate functional relationships among switch genes and to verify if they localized in densely connected protein complex using the MCODE algorithm implemented with BioGrid, InWeb_IM, OmniPath annotations.

### 4.6. Cell Culture and Treatment

Human normal breast cell line, MCF10A (ATCC^®^ CRL-10317, American Type Culture Collection 10801 University Boulevard Manassas, VA 20110 USA) and breast cancer cell lines, MCF-7 (DSMZ # ACC 115, Inhoffenstraße 7B, 38124 Braunschweig, Germany), T47D (DSMZ # ACC 739), BT-474 (DSMZ # ACC 64), MDA-MB-361 (ATCC^®^ HTB-27), MDA-MB-453 (DSMZ # ACC 65), UACC-893 (ATCC^®^ CRL-1902), SK-BR3 (DSMZ # ACC 736), MDA-MB-231 (DSMZ # ACC 731), BT549 (ATCC^®^ HTB-122), Hs-578T (DSMZ # ACC 781) were cultured according to manufacture recommendations. Briefly, MCF10A were cultured in MEGM (Mammary Epithelial Cell Growth Medium; MEBM^®^) supplemented with BPE, hEGF, insulin, and hydrocortisone (MEGM^®^SingleQuots, Lonza, Walkersville, MD, USA). MCF-7 cells were grown in RPMI (Roswell Park Memorial Institute, Life Technologies, USA) medium supplemented with 10% fetal bovine serum (FBS), with insulin (0.01 mg/mL), 2 mM L-glutamine, and 1% of non-essential amino acids. T-47D were maintained in RPMI supplemented with 10% FBS, insulin (0.01 mg/mL) and 2 mM L-glutamine. BT-474 were grown in RPMI supplemented with 20% FBS, with insulin (0.01 mg/mL) and 2 mM L-glutamine. MDA-MB-361 were maintained in Leibovitz’s L-15 medium supplemented with 20% FBS and 2 mM L-glutamine. MDA-MB-453, UACC-893, and MDA-MB-231 were grown in Leibovitz’s L-15 medium supplemented with 10% FBS and 2 mM L-glutamine. SK-BR3 were cultured in McCoy’s 5a Medium completed by 20% FBS and 2 mM L-glutamine. Hs-578T was maintained in DMEM (Dulbecco’s Modified Eagle’s medium, Life Technologies, USA) supplemented by 10% FBS and 2 mM L-glutamine. BT549 were cultured in RPMI supplemented with 10% FBS, insulin (0.01 mg/mL) and 2 mM L-glutamine. All cell lines were cultured in a humidified 5% CO_2_ atmosphere at 37 °C, except to MDA-MB-361, MDA-MB-453, UACC-893, and MDA-MB-231, because cultured in Leibovitz’s L-15 medium.

Alisertib (MLN8237) was purchased from Selleckchem Inc. (Houston, TX, USA). The drug was dissolved in dimethyl sulfoxide according to the manufacturer’s instructions and stored at −80°C. It was used to treat cells at 0.25, 0.5, 1 and 2 µM final concentrations for 24, 48, and 72 h.

### 4.7. RNA Extraction, Real-Time PCR, and Tissues QPCR Array

Total RNA was isolated using TRIzol reagent (Invitrogen, USA). The RNA concentration was determined using the Qubit RNA HS Assay Kit (Thermo Fisher Scientific, USA) and 1 μg total RNA was reverse transcribed and used for real-time PCR experiments. For cultured cells, expression value of AURKA, CCNB1, CCNB2, CDC20, CDC45, CDK1, ESPL1, NEK2, PTTG1, and RAD54L was determined by real-time PCR using specific primers (Qiagen), and QuantiTect SYBR Green PCR Kit (Qiagen, Germany). RT-PCR conditions were 95 °C for 15 min, 40 cycles of 94 °C for 15 s, 55° C for 30 s and 72 °C 30 s. The maximum cycle threshold (Ct) value was set at 40. GAPDH and B2M were used as housekeeping control genes as suggested [45], using the CFX Maestro Software (Biorad, USA). Experiments were carried out in triplicate for each data point, and data analysis was done by using CFX Maestro Software (Biorad, USA).

The expression value of AURKA, ESPL1, RAD54L and CDC45 in human breast tissue was quantified by real-time PCR using cDNA panels of 43 breast tumor and 5 normal breast tissue samples arrayed onto a single 96-well reaction plate by OriGene (TissueScan, Breast Cancer cDNA Array II-BCRT502, OriGene Technologies, Rockville, USA). For RT-PCR were used the aforementioned condition. Data were expressed as mean fold-change using the comparative 2−ΔΔCq method (compared to non-malignant control tissue). β-Actin was used as the housekeeping gene.

### 4.8. Immunohistochemistry

A total of 27 formalin-fixed and paraffin-embedded breast specimens (FFPE) (4 normal and 23 tumor tissues divided as follow: 6 Luminal A, 5 Luminal B, 8 Her2 positive and 4 Triple negative) were collected at the Ospedale Evangelico Betania, (Naples, Italy) and preserved at SDN Biobank [46] (Naples, Italy). Clinicopathological characteristics of the study subjects are included in Table 4.

This study was approved by the Ethics Committee of IRCCS Pascale (Naples, Italy) (Protocol n. 1/16 OSS SDN). Written informed consent was obtained from all subjects. The study was conducted anonymously and conforms to the principles of the Helsinki Declaration. The hematoxylin and eosin and IHC reports were used to characterize the BC tumor subtypes of each specimen analyzed. For each case, we selected a block of tissue of the tumor and used it to obtain 4 µm thick sections mounted on Matsunami TOMO^®^ hydrophilic adhesion slides for immunohistochemistry assay. The anti-AURKA staining and assay were performed with a Ventana BenchMark ULTRA immunostainer (Roche Diagnostics, Basel, Switzerland) using diaminobenzidine as chromogen and with reagents registered according to the manufacturer’s protocol (Ultra View Universal DAB detection kit; Ventana, Tucson, AZ). The manual incubation procedure was performed using antibody anti-AURKA (D3V7T) XP Rabbit mAb (Cell Signaling: #91590; dilution 1:400. Cell Signaling Technology, Inc. USA). Antigen expression was evaluated by an expert pathologist using a direct light microscope in a bright field at 20× and 40× magnification.

### 4.9. Protein Isolation and Western Blotting

Cells were washed twice in ice-cold PBS and lysed in JS buffer (50 mmol/L HEPES (pH 7.5) containing 150 mmol/L NaCl, 1% glycerol, 1% Triton X100, 1.5 mmol/L MgCl2, 5 mmol/L EGTA, 1 mmol/L Na3VO4, and 1× protease inhibitor cocktail). Protein concentration was determined by the Bradford assay (Biorad, USA) using bovine serum albumin as the standard, and equal amounts of proteins were analyzed by SDS-PAGE (TGX Stain-Free Precast Gels (8/16%), Biorad, USA). Gels were electroblotted onto polyvinylidene difluoride membranes (Millipore). For immunoblot experiments, membranes were blocked for 1 h with 5% nonfat dry milk in TBS containing 0.1% Tween 20 and incubated at 4 °C overnight with primary antibody. Subsequently, the membranes were incubated with an HRP-conjugated secondary antibody (Biorad, USA) at room temperature for 1 h and were visualized at ChemiDoc Imaging System (Biorad, USA) using enhanced chemiluminescence reagents (Clarity Max Western ECL Substrate, Biorad, USA) according to the manufacturer’s instructions. The antibodies used were as follows: anti-AURKA (Cell Signaling: #91590; 1:1000, Cell Signaling Technology, Inc. USA), and anti-β-actin (Sigma; 1:4000. Merck KGaA, Germany).

### 4.10. Cell Death Assessment by Annexin V Staining and Cell-Cycle Analysis

Apoptotic cells were identified using the Annexin V-FITC/AAD Kit (Beckman–Coulter). The PBS-washed cells were resuspended in ice-cold 1X Binding Buffer and incubated with Annexin V-FITC and 7-amino-actinomycin D (7-AAD) on ice, for 15 minutes in the dark according to manufacturer’s instructions. All samples were analyzed within 30 minutes by flow cytometry using the Cytoflex V2-B4-R2 (Beckman–Coulter, CA, USA) instrument. Twenty thousand events were acquired per sample.

The COULTER DNA (Beckman–Coulter) Prep kit was used to assess the cell cycle of human breast cancer cells. The DNA PREP LPR reagent was used to permeabilize the cells and DNA staining solution containing propidium iodide (PI) to stain DNA content. After PI staining, the quantification of the cell-cycle distribution was carried out using the Cytoflex flow cytometer. Cell-cycle analysis was performed using the J.V. Watson algorithm. All data were analyzed using Kaluza analysis software (Beckman–Coulter, CA, USA). The antibodies used were as follows: anti-AURKA (Cell Signaling: #91590; 1:1000), and anti-β-actin (Sigma; 1:4000).

## 5. Conclusions

Overall, we identified a disease module composed of four hub genes (switch genes) functionally interconnected and over-expressed in BC in a subtype-independent manner. This pathological molecular signature, as a part of a common perturbated disease module, could bypass the limit of BC heterogeneity paving the way for a more accurate discovery of potential treatment targets for BC patients regardless of tumor subtype. This is in full accordance with the vision of Network Medicine that proposes previously unrecognized molecular definitions of disease phenotypes, overcoming the most canonical organ- or symptom-based definitions, and, at the same time, suggests putative targets to identify, prevent, and treat diseases.

## Figures and Tables

**Table 1:** This table reports the parameters and the results for each comparison *subtype vs normal*.

	IHC Classification				PAM50 Classification			
	Lum HER2−	Lum B (HER2+)	HER2+ (Non-Luminal)	Triple Negative	Luminal A	Luminal B	HER2 Enriched	Basal-Like
**Samples**								
# normal	111	111	111	111	111	111	111	111
# tumor	574	123	37	153	229	120	58	98
**Thresholds**								
FC threshold	4	4	4	4	4	4	4	4
FDR threshold	0.05	0.05	0.05	0.05	0.05	0.05	0.05	0.05
PC threshold	0.6	0.72	0.75	0.69	0.64	0.63	0.68	0.66
**DEGs**								
# total	1365	1603	1862	1714	1338	2062	1954	1829
# up	363 (27%)	448 (28%)	496 (27%)	515 (30%)	373 (28%)	468 (23%)	540 (28%)	510 (28%)
# down	1002 (73%)	1155 (72%)	1366 (73%)	1199 (70%)	965 (72%)	1594 (77%)	1414 (72%)	1319 (72%)
**Switch**								
# total	84	261	278	251	222	358	363	343
# up	84 (100%)	261 (100%)	272 (98%)	217 (86%)	221 (99.5%)	356 (99%)	363 (100%)	329 (96%)
# down	0	0	6 (2%)	34 (14%)	1 (0.5%)	2 (1%)	0	14 (4%)

Abbreviations: DEGs (Differentially expressed genes); FC (Fold-change); FDR (False Discovery Rate); PC (Pearson Correlation); # (number).

**Table 2:** Definition of clinical IHC and The Cancer Genome Atlas (TCGA)-BRCA breast cancer subtypes.

IHC Clinicopathologic Surrogate Definition	TCGA-BRCA Clinicopathologic Surrogate Definition
**‘Luminal A-like’** all: ER and PgR positive, HER2 negative, Ki-67 ‘low’.	**‘Luminal HER2 negative’** all: ER and PgR positive, HER2 negative.
**‘Luminal B-like (HER2 negative)’** ER positive HER2 negative and at least one of: Ki-67 ‘high’, PgR ‘negative or low’.	
**‘Luminal B-like (HER2 positive)’** ER positive, HER2 over-expressed or amplified, Any Ki-67 and Any PgR.	**‘Luminal B-like (HER2 positive)’** ER positive, HER2 over-expressed or amplified, Any PgR.
**‘HER2 positive (non-luminal)’** HER2 over-expressed or amplified, ER and PgR absent.	**‘HER2 positive (non-luminal)’** HER2 over-expressed or amplified, ER and PgR absent.
**‘Triple negative’** ER and PgR absent, HER2 negative.	**‘Triple negative’** ER and PgR absent, HER2 negative.

**Table 3:** List of 11 S switches intersected between IHC and PAM50 classifications.

Gene Name	Gene Description	Location	Type(s)	Gene Stable ID	Drug(s)
*AURKA*	aurora kinase A	Nucleus	kinase	ENSG00000087586	ilorasertib, MK 5108, SNS 314, AT-9283, alisertib, MLN8054, TTP607, CYC 116, TAS-119, tozasertib, AMG 900, danusertib
*CCNB1*	cyclin B1	Cytoplasm	kinase	ENSG00000134057	bertilimumab
*CCNB2*	cyclin B2	Cytoplasm	other	ENSG00000157456	-
*CDC20*	cell division cycle 20	Nucleus	other	ENSG00000117399	-
*CDC45*	cell division cycle 45	Nucleus	other	ENSG00000093009	-
*CDK1*	cyclin dependent kinase 1	Nucleus	kinase	ENSG00000170312	AZD5438, SB-1317, alvocidib, AG 024322, milciclib, riviciclib, roniciclib, dinaciclib
*ESPL1*	extra spindle pole bodies like 1, separase	Nucleus	peptidase	ENSG00000135476	-
*NEK2*	NIMA related kinase 2	Cytoplasm	kinase	ENSG00000117650	-
*PLK1*	polo like kinase 1	Nucleus	kinase	ENSG00000166851	lipid encapsulated anti-PLK1 siRNA TKM-080301, onvansertib, GSK461364, TAK-960, MK1496, rigosertib, volasertib, BI 2536
*PTTG1*	PTTG1 regulator of sister chromatid separation, securin	Nucleus	transcription regulator	ENSG00000164611	-
*RAD54L*	RAD54 like	Nucleus	enzyme	ENSG00000085999	-

**Table 4:** Clinicopathological characteristics of the study subjects.

**N° Healthy Control**	4
Age 40.75 (39–55 years)	
**N° Breast Cancer Patients**	23
Age 63.39 (41–82 years)	
**Histological Type**	
Invasive Ductal carcinoma	23
Hyperplasia	2
Gynecomastia	2
**Subtype**	
Luminal A	6
Luminal B	5
Her2+	8
Triple negative	4
**Ki67**	
Low (0–29%)	15
High (30–100%)	8
**Grade**	
G1	3
G2	12
G3	8
**Tumor Size (cm)**	
0.1–2	15
2–5	6
>5	2

## Supplementary Materials

The following are available online at https://www.mdpi.com/1422-0067/21/18/6690/s1, **Figure S1:** The probability distribution of APCC for each breast subtype; **Figure S2:** Venn diagrams of switch genes identified by the SWIM tool; **Figure S3:** Heatmaps for switch genes of each breast subtype. Heatmaps representing the expression profiles of switch genes (rows) identified for each breast subtype of IHC classification (upper panel) and PAM50 classification (bottom panel) across all tumor samples (columns). The expression profiles of switch genes were clustered according to rows by using Pearson correlation distance as metrics and the tumor samples were grouped and colored according to the specific breast subtype they belong. Heatmap colors represent different expression levels (Z-score normalized) that increase from blue to yellow; **Figure S4:** Match between IHC and PAM50 classification switch genes defining Intersected Specific-Subtype (ISS) switches; **Figure S5:** Protein–protein interaction (PPI) network of the 12 Intersected IHC–PAM50 switches (IS switches); **Figure S6:** Switch gene expression in BC cell lines vs control cell; **Figure S7:** Distribution of switch genes among luminal and not luminal BC subtypes; **Figure S8:** Distribution of switch genes among G1-G2 and G3; **Figure S9:** Distribution of switch genes among disease Stages. **Table S1**. Network-based separation values and their corresponding *p*-values computed between each pair of switch genes’ modules of PAM50 classification and IHC classification, related to Figure 3B,C; **Table S2:** Ingenuity Pathway Analysis (IPA) heatmap for IHC stratification switch genes, related to Figure 4A; **Table S3:** Ingenuity Pathway Analysis (IPA) heatmap for PAM50 stratification switch genes, related to Figure 4B; **Table S4:** List of IHC shared switch enriched in statistically significant pathways and their IPA knowledge base annotations, related to Figure 4A; **Table S5:** List of IHC subtype-specific switch enriched in statistically significant pathways and their IPA knowledge base annotations, related to Figure 4A; **Table S6:** List of PAM50 shared switch enriched in statistically significant pathways and their IPA knowledge base annotations, related to Figure 4B; **Table S7:** List of PAM50 subtype-specific switch enriched in statistically significant pathways and their IPA knowledge base annotations, related to Figure 4B; **Table S8:** Unique S-switches between IHC and PAM50 classification, annotations, and CancerMine database citations. **Table S9:** TCGA-BRCA clinicopathologic surrogate definition.
