# Supplementary material for: The New Paradigm of Network Medicine to Analyze Breast Cancer Phenotypes
Source: Int J Mol Sci. 2020 Sep 12;21(18):6690. doi: 10.3390/ijms21186690 (PMC7555916; doi:10.3390/ijms21186690)
Supplement: Supplementary file 1 [file ijms-21-06690-s001.zip › S-Figures_IJMS.pptx]

## Slide 1
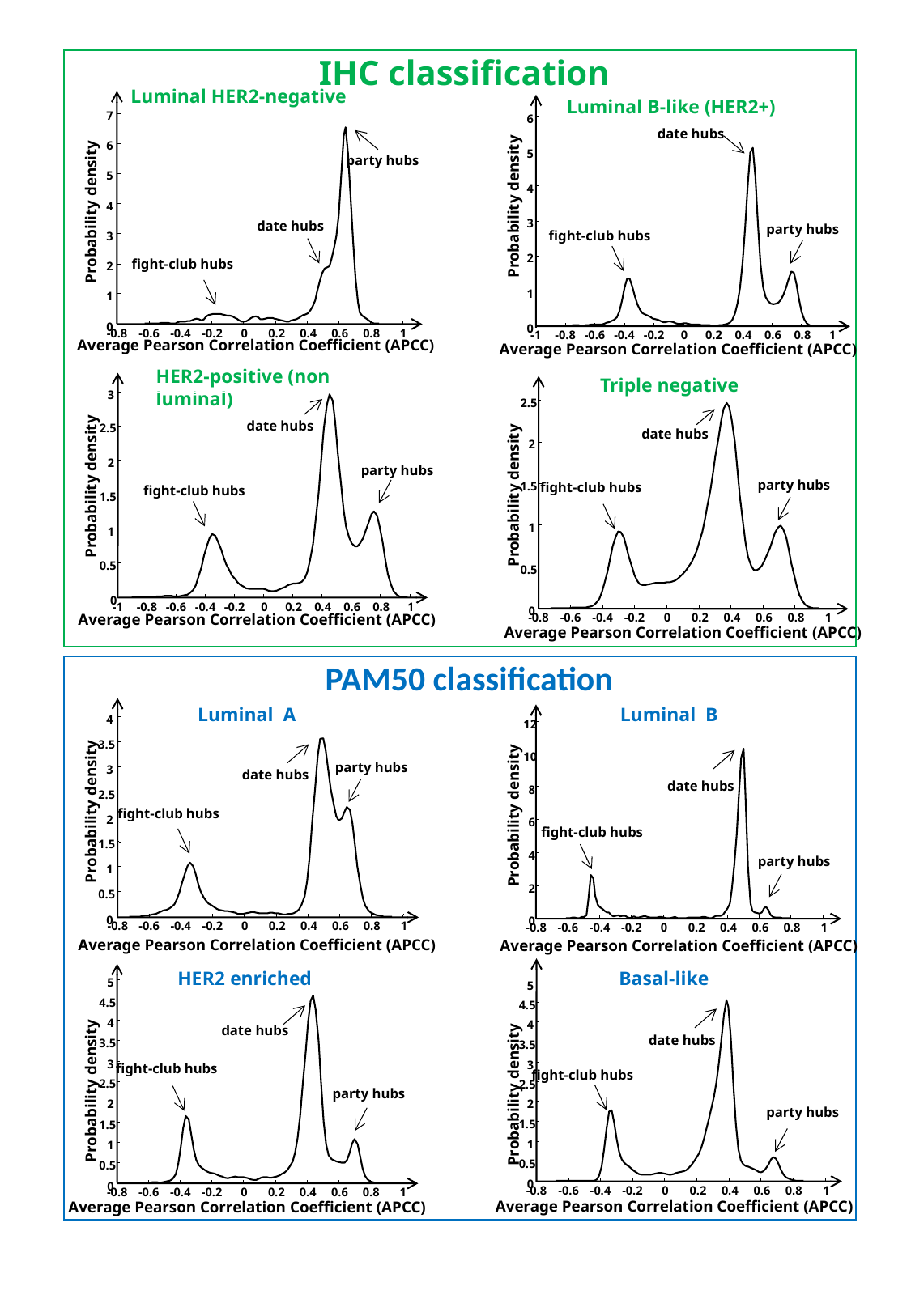

IHC classification
Luminal HER2-negative
7
6
5
4
3
2
1
0
-0.8
-0.6
-0.4
-0.2
0
0.2
0.4
0.6
0.8
1
Probability density
party hubs
date hubs
fight-club hubs
Average Pearson Correlation Coefficient (APCC)
Luminal B-like (HER2+)
6
5
4
3
2
1
0
-1
-0.8
-0.6
-0.4
-0.2
0
0.2
0.4
0.6
0.8
1
Probability density
date hubs
party hubs
fight-club hubs
Average Pearson Correlation Coefficient (APCC)
HER2-positive (non luminal)
3
2.5
2
1.5
1
0.5
0
-1
-0.8
-0.6
-0.4
-0.2
0
0.2
0.4
0.6
0.8
1
date hubs
party hubs
Probability density
fight-club hubs
Average Pearson Correlation Coefficient (APCC)
Triple negative
2.5
2
1.5
1
0.5
0
-0.8
-0.6
-0.4
-0.2
0
0.2
0.4
0.6
0.8
1
date hubs
party hubs
fight-club hubs
Probability density
Average Pearson Correlation Coefficient (APCC)
PAM50 classification
Luminal A
Luminal B
4
3.5
3
2.5
2
1.5
1
0.5
0
-0.8
-0.6
-0.4
-0.2
0
0.2
0.4
0.6
0.8
1
12
10
8
6
4
2
0
-0.8
-0.6
-0.4
-0.2
0
0.2
0.4
0.6
0.8
1
party hubs
date hubs
date hubs
Probability density
Probability density
fight-club hubs
fight-club hubs
party hubs
Average Pearson Correlation Coefficient (APCC)
Average Pearson Correlation Coefficient (APCC)
5
4.5
4
3.5
3
2.5
2
1.5
1
0.5
0
-0.8
-0.6
-0.4
-0.2
0
0.2
0.4
0.6
0.8
1
HER2 enriched
Basal-like
5
4.5
4
3.5
3
2.5
2
1.5
1
0.5
0
-0.8
-0.6
-0.4
-0.2
0
0.2
0.4
0.6
0.8
1
date hubs
date hubs
fight-club hubs
fight-club hubs
Probability density
Probability density
party hubs
party hubs
Average Pearson Correlation Coefficient (APCC)
Average Pearson Correlation Coefficient (APCC)

## Slide 2
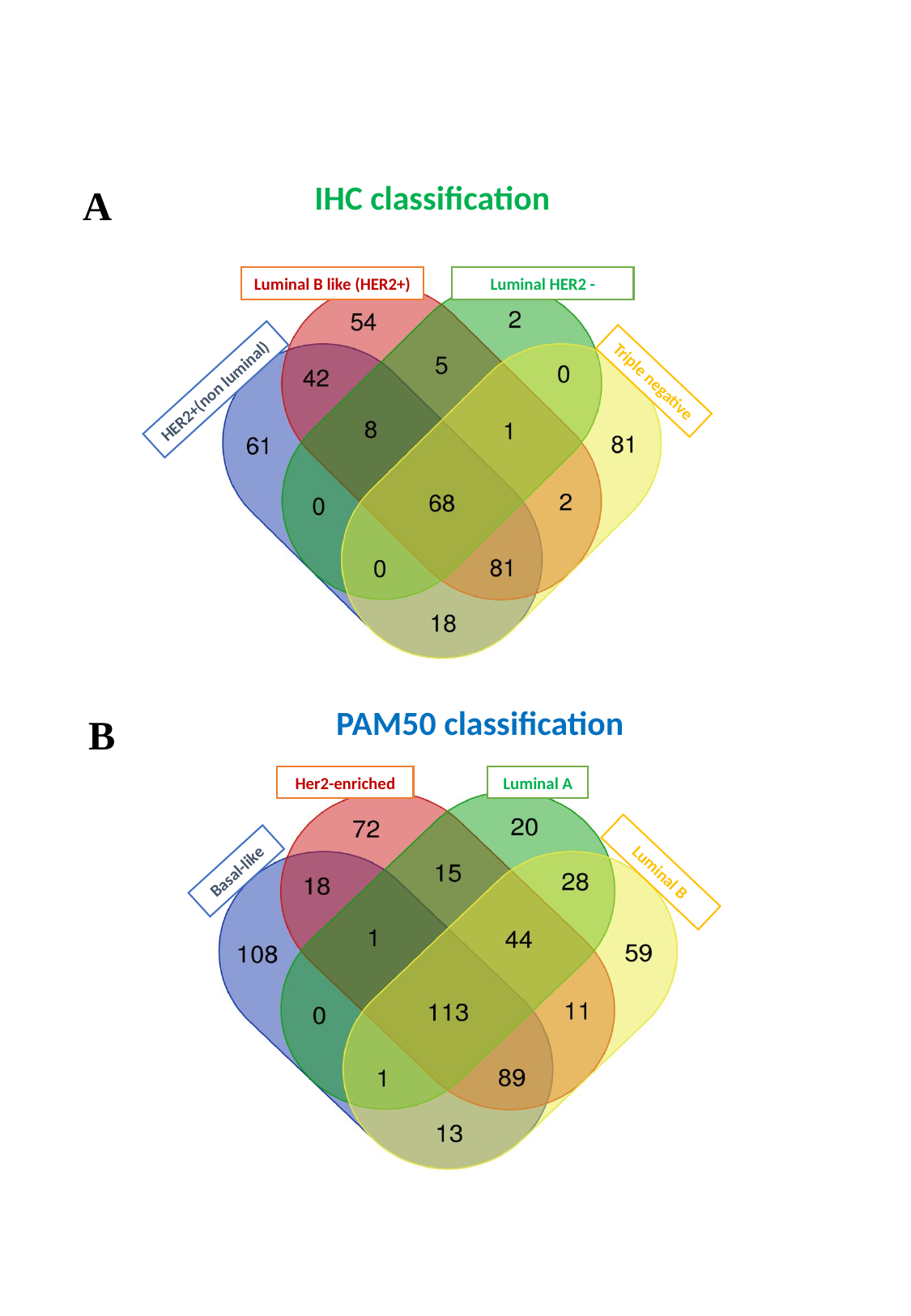

IHC classification
A
Luminal B like (HER2+)
Luminal HER2 -
Triple negative
HER2+(non luminal)
PAM50 classification
B
Her2-enriched
Luminal A
Basal-like
Luminal B

## Slide 3
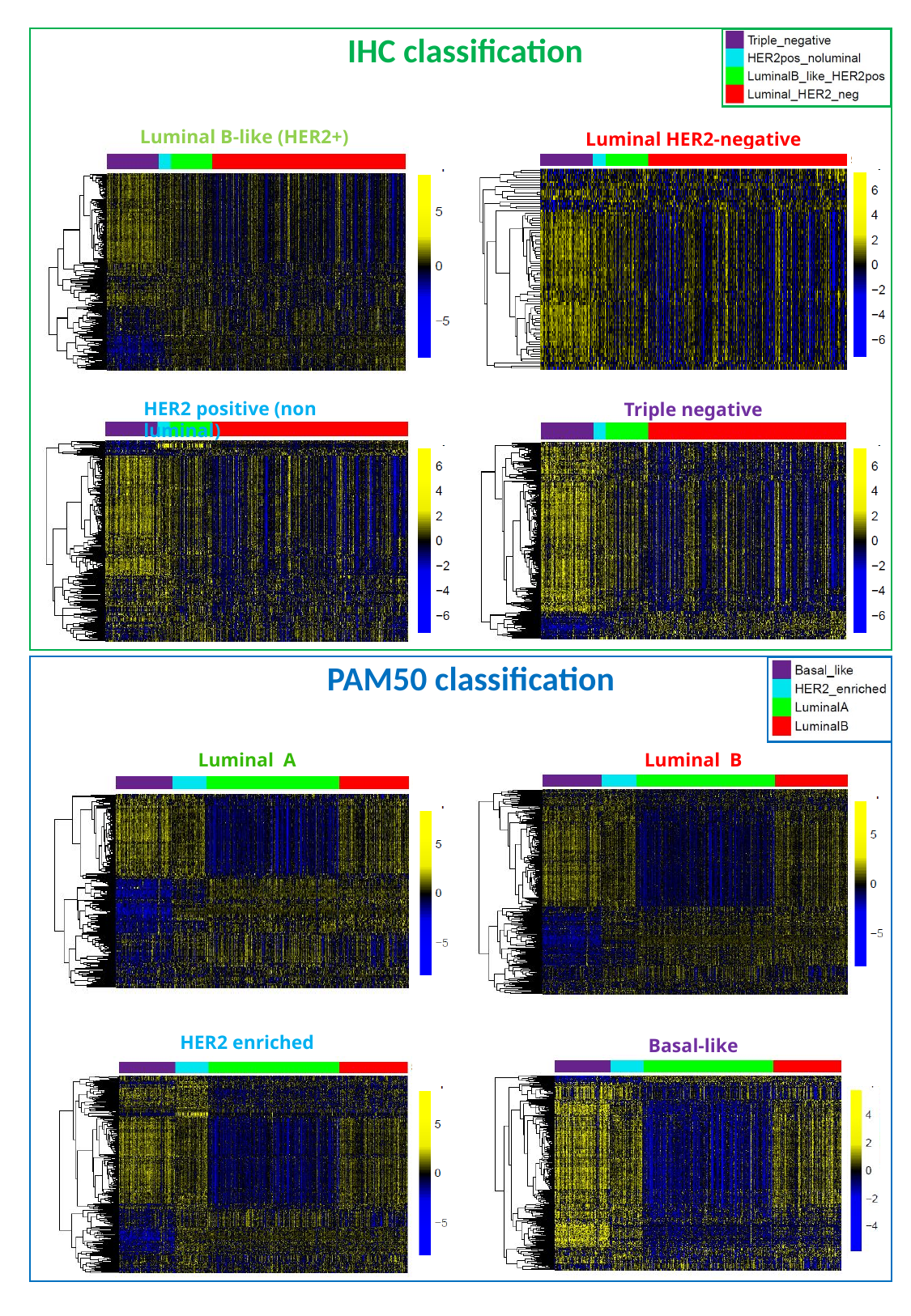

IHC classification
Luminal B-like (HER2+)
Luminal HER2-negative
HER2 positive (non luminal)
Triple negative
PAM50 classification
Luminal A
Luminal B
HER2 enriched
Basal-like

## Slide 4
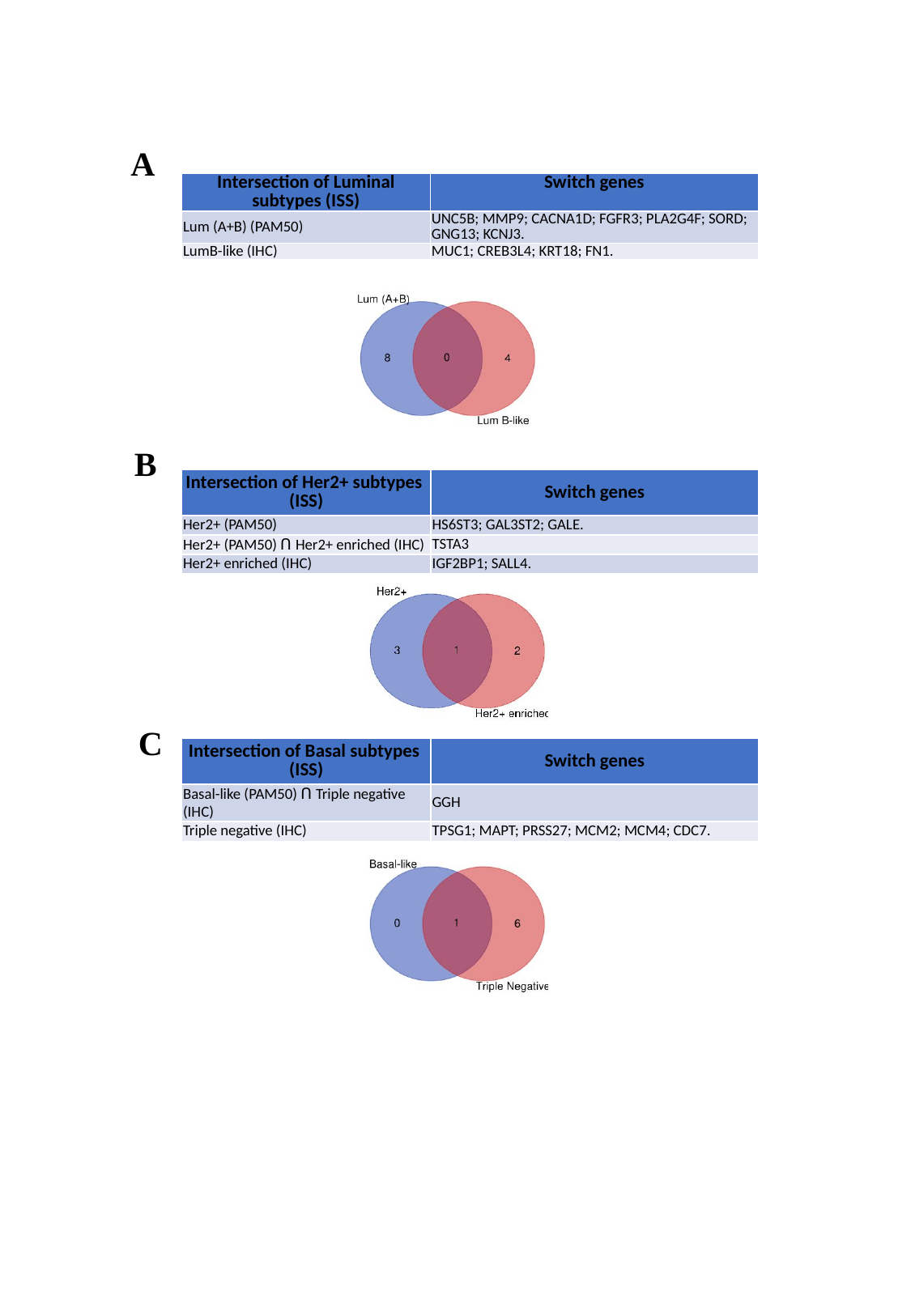

A
| Intersection of Luminal subtypes (ISS) | Switch genes |
| --- | --- |
| Lum (A+B) (PAM50) | UNC5B; MMP9; CACNA1D; FGFR3; PLA2G4F; SORD; GNG13; KCNJ3. |
| LumB-like (IHC) | MUC1; CREB3L4; KRT18; FN1. |
B
| Intersection of Her2+ subtypes (ISS) | Switch genes |
| --- | --- |
| Her2+ (PAM50) | HS6ST3; GAL3ST2; GALE. |
| Her2+ (PAM50) ꓵ Her2+ enriched (IHC) | TSTA3 |
| Her2+ enriched (IHC) | IGF2BP1; SALL4. |
C
| Intersection of Basal subtypes (ISS) | Switch genes |
| --- | --- |
| Basal-like (PAM50) ꓵ Triple negative (IHC) | GGH |
| Triple negative (IHC) | TPSG1; MAPT; PRSS27; MCM2; MCM4; CDC7. |

## Slide 5
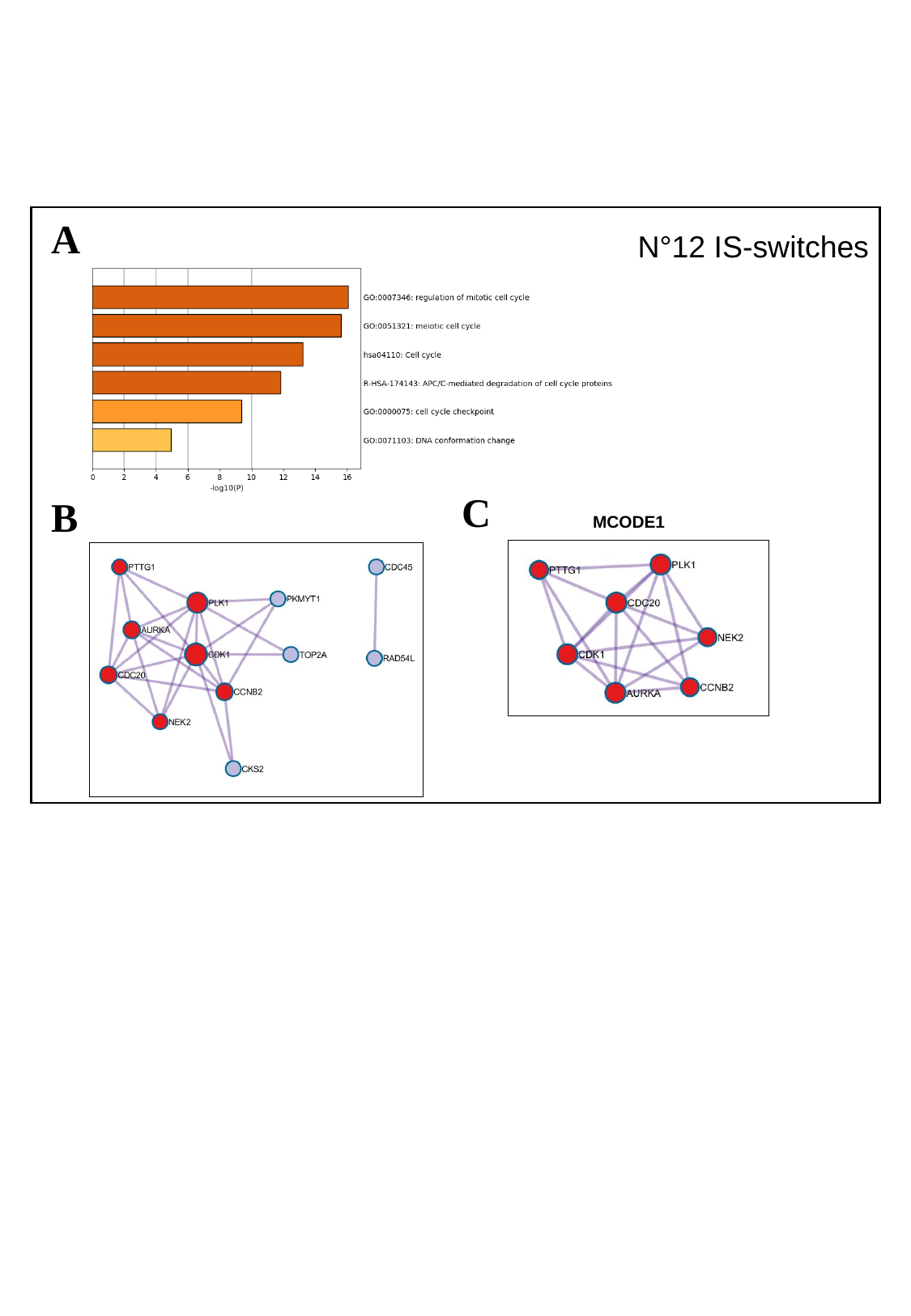

A
N°12 IS-switches
C
B
MCODE1

## Slide 6
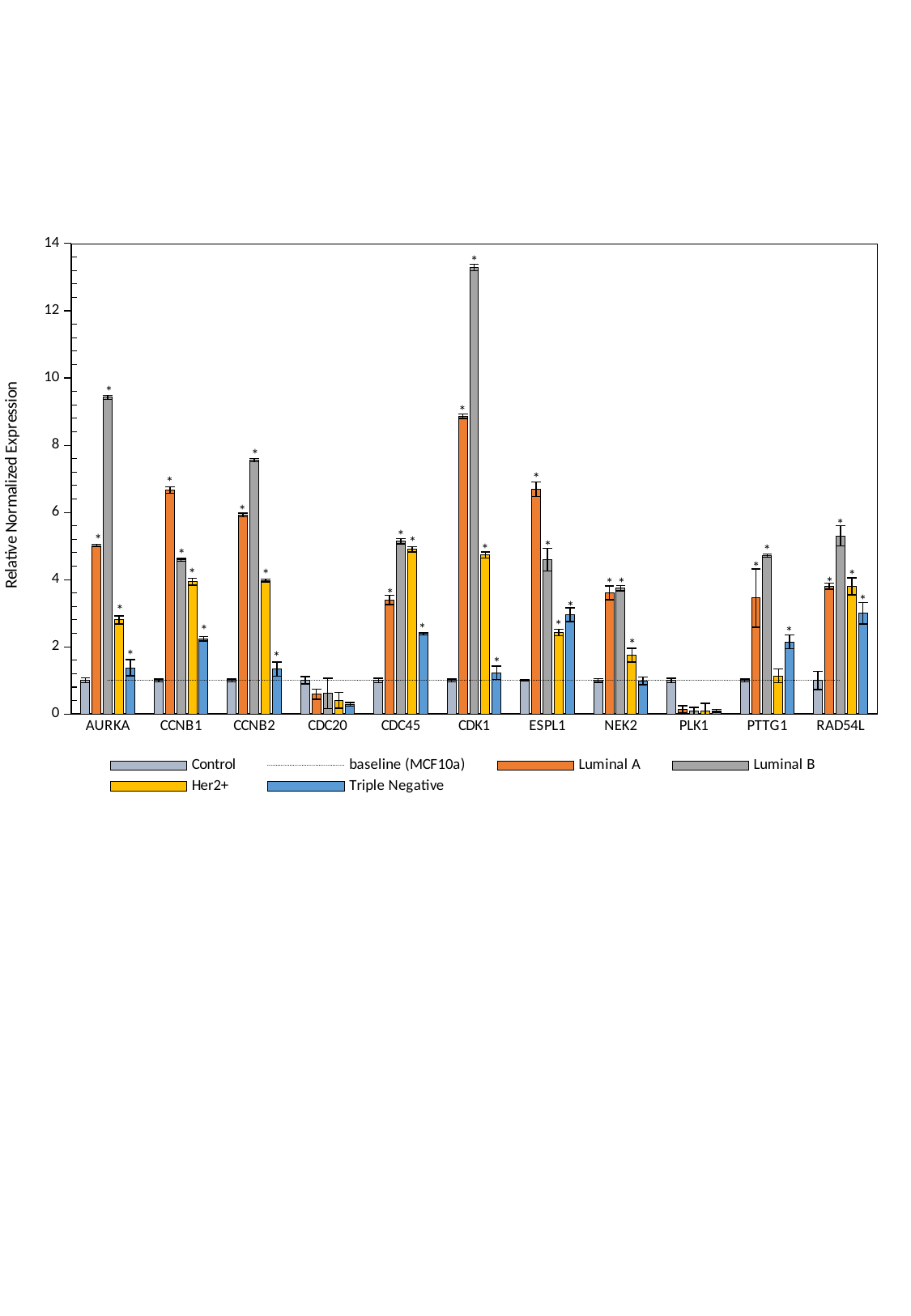

### Chart
| Category | Control | Luminal A | Luminal B | Her2+ | Triple Negative |
|---|---|---|---|---|---|
| AURKA | 1.0 | 5.011972731488303 | 9.42692219997308 | 2.7997273319682807 | 1.3756205925976168 |
| CCNB1 | 1.0 | 6.669454679226181 | 4.581575113585733 | 3.932664221587123 | 2.2381842893391446 |
| CCNB2 | 1.0 | 5.92637919341286 | 7.556966433024325 | 3.9766455824028273 | 1.3306071909275474 |
| CDC20 | 1.0 | 0.5941434447309019 | 0.6104073730975299 | 0.40743388904465067 | 0.2862626293040964 |
| CDC45 | 1.0 | 3.3899801044775577 | 5.141352991552056 | 4.8937918030782575 | 2.3926290729576247 |
| CDK1 | 1.0 | 8.860378714275168 | 13.286303737858125 | 4.728974022198735 | 1.2267868769254526 |
| ESPL1 | 1.0 | 6.691626784940119 | 4.5929078831380545 | 2.4255768058927782 | 2.9538227394419674 |
| NEK2 | 1.0 | 3.605917281186359 | 3.7420267055871674 | 1.750963882635603 | 0.985414369627132 |
| PLK1 | 1.0 | 0.1411328821963939 | 0.09766573813301763 | 0.07893002911239771 | 0.08975253232170473 |
| PTTG1 | 1.0 | 3.4530449481514394 | 4.713920383552296 | 1.133637947344591 | 2.147024972783559 |
| RAD54L | 1.0 | 3.8 | 5.3 | 3.8 | 3.0 |*
*
*
*
*
*
*
*
*
*
*
*
*
*
*
*
*
*
*
*
*
*
*
*
*
*
*
*
*
*
*
*
*
*

## Slide 7
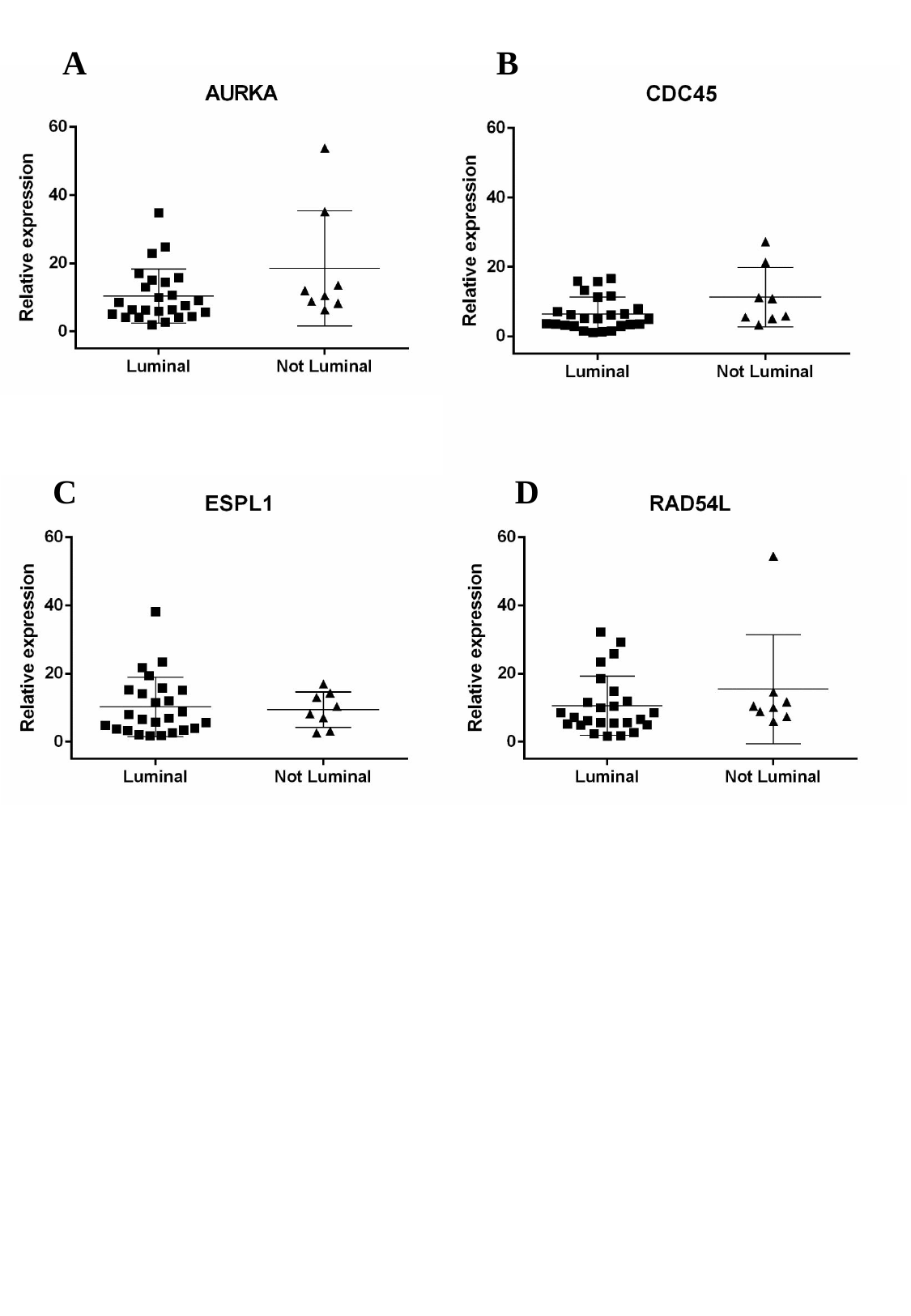

A
B
C
D

## Slide 8
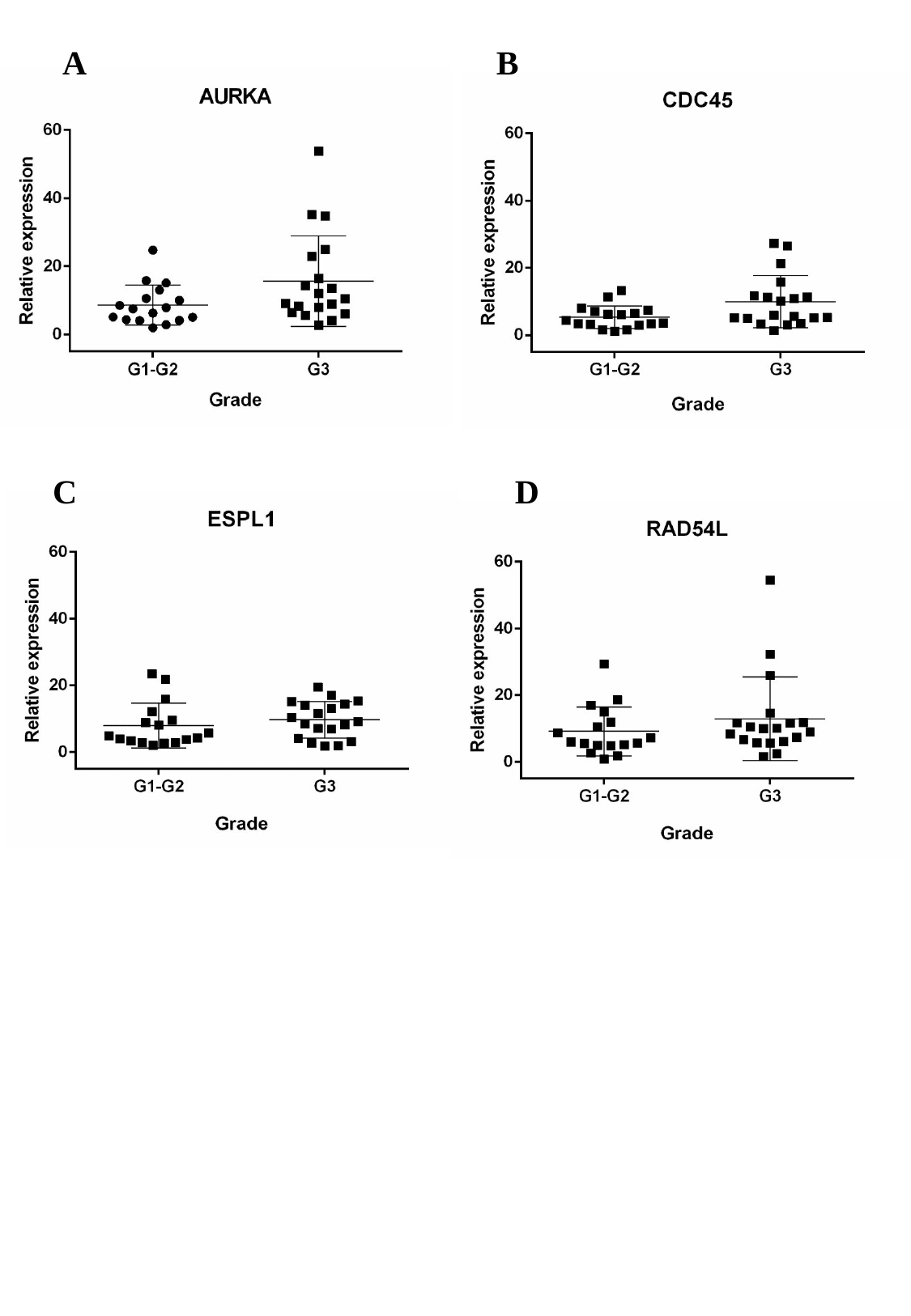

A
B
C
D

## Slide 9
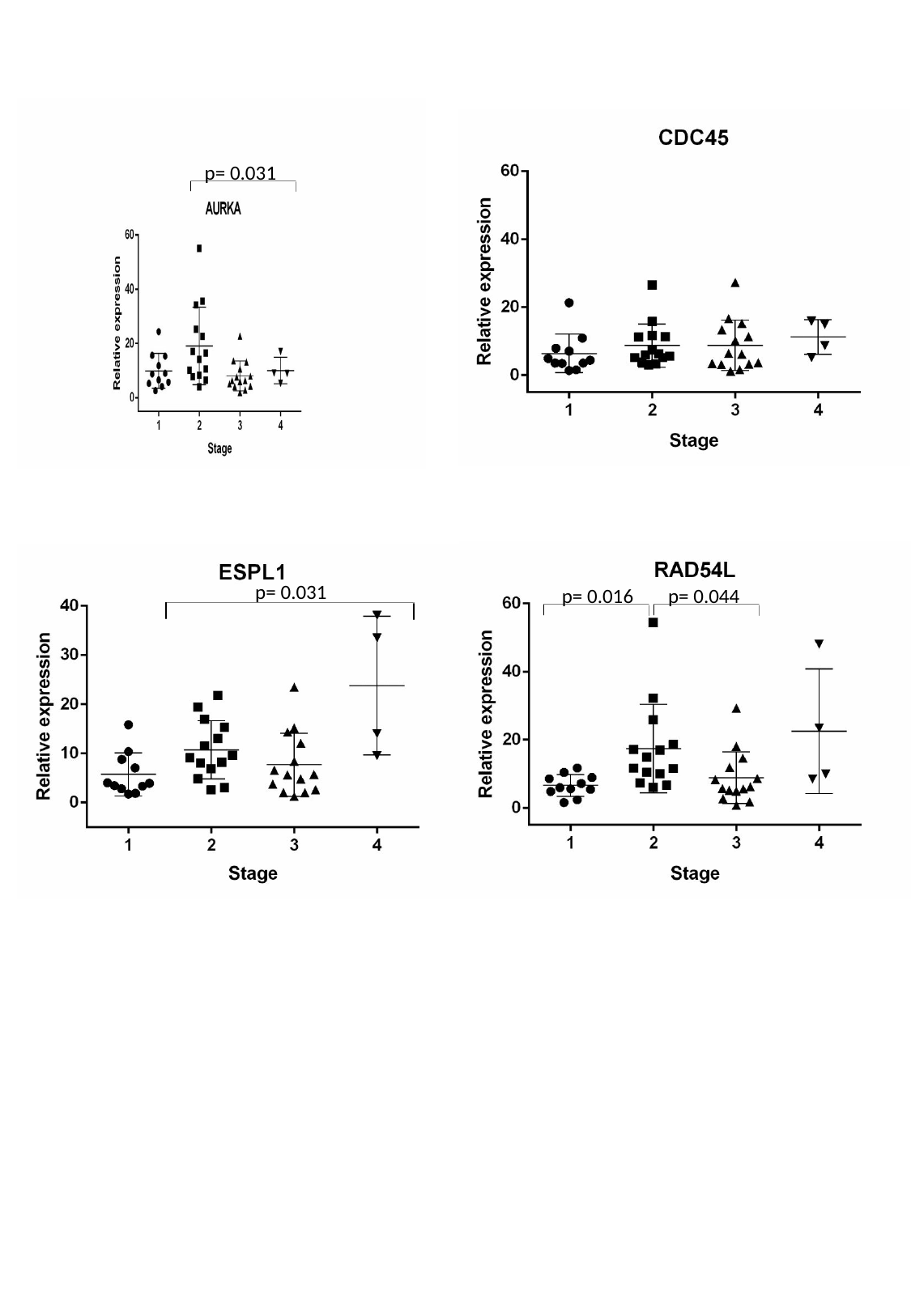

p= 0.031
p= 0.031
p= 0.016
p= 0.044
