# Supplementary material for: The New Paradigm of Network Medicine to Analyze Breast Cancer Phenotypes
Source: Int J Mol Sci. 2020 Sep 12;21(18):6690. doi: 10.3390/ijms21186690 (PMC7555916; doi:10.3390/ijms21186690)
Supplement: Supplementary file 1 [file ijms-21-06690-s001.zip › Table S1.docx]

**Table S1.** Network-based separation values and their corresponding p-values computed between each pair of switch genes’ modules of PAM50 classification and IHC classification, related to Figure 3B-C.

| **PAM50** | | | | |
| --- | --- | --- | --- | --- |
| **separation** | **Luminal A** | **Luminal B** | **HER2 enriched** | **Basal-like** |
| Luminal A | 0.00 | -0.95 | -0.86 | -0.58 |
| Luminal B | -0.95 | 0.00 | -1.07 | -0.91 |
| HER2 enriched | -0.86 | -1.07 | 0.00 | -0.91 |
| Basal-like | -0.58 | -0.91 | -0.91 | 0.00 |
|  |  |  |  |  |
| **p-values** | **Luminal A** | **Luminal B** | **HER2 enriched** | **Basal-like** |
| Luminal A | 0 | 6.77E-143 | 1.07E-160 | 5.51E-57 |
| Luminal B | 6.77E-143 | 0 | 5.65E-305 | 2.40E-185 |
| HER2-enriched | 1.07E-160 | 5.65E-305 | 0 | 6.71E-178 |
| Basal-like | 5.51E-57 | 2.40E-185 | 6.71E-178 | 0 |

| **IHC** | | | | |
| --- | --- | --- | --- | --- |
| **separation** | **Luminal HER2-** | **Luminal B like (HER2+)** | **HER2+(non-luminal)** | **Triple Negative** |
| Luminal HER2- | 0.00 | -0.61 | -0.52 | -0.47 |
| Luminal B like (HER2+) | -0.61 | 0.00 | -1.11 | -0.81 |
| HER2+(non-luminal) | -0.52 | -1.11 | 0.00 | -0.88 |
| Triple Negative | -0.47 | -0.81 | -0.88 | 0.00 |
|  |  |  |  |  |
| **p-values** | **Luminal HER2-** | **Luminal B like (HER2+)** | **HER2+ (non-luminal)** | **Triple Negative** |
| Luminal HER2- | 0 | 7.62E-63 | 1.31E-28 | 2.47E-32 |
| Luminal B like (HER2+) | 7.62E-63 | 0 | 5.44E-275 | 1.03E-147 |
| HER2+ (non-luminal) | 1.31E-28 | 5.44E-275 | 0 | 1.43E-173 |
| Triple Negative | 2.47E-32 | 1.03E-147 | 1.43E-173 | 0 |
