# Supplementary material for: The New Paradigm of Network Medicine to Analyze Breast Cancer Phenotypes
Source: Int J Mol Sci. 2020 Sep 12;21(18):6690. doi: 10.3390/ijms21186690 (PMC7555916; doi:10.3390/ijms21186690)
Supplement: Supplementary file 1 [file ijms-21-06690-s001.zip › Table S2.docx]

**Table S2.** Ingenuity Pathway analysis (IPA) heatmap for IHC stratification switch genes, related to Figure 4A.

| **IHC STRATIFICATION CANONICAL PATHWAYS** | **SHARED TO ALL SUBTYPES (S)^[[1]](#footnote-1)^** | **SUBTYPE-SPECIFIC (SS)^[[2]](#footnote-2)^** | | | |
| --- | --- | --- | --- | --- | --- |
|  |  | **Lum HER 2 negative** | **Lum B like** | **HER 2+** | **Triple negative** |
| Cell Cycle: G2/M DNA Damage Checkpoint Regulation | 13.63593461 | 0 | 0 | 0 | 0.797923944 |
| Mitotic Roles of Polo-Like Kinase | 10.63490285 | 0 | 0 | 0 | 1.641927515 |
| Cell Cycle Control of Chromosomal Replication | 4.598994368 | 0 | 0 | 0 | 2.989217105 |
| ATM Signaling | 3.664811578 | 0 | 0.681524039 | 0 | 1.335613777 |
| DNA damage-induced 14-3-3σ Signaling | 4.603185442 | 0 | 0 | 0 | 0 |
| Pyrimidine Ribonucleotides Interconversion | 2.10278887 | 0 | 0 | 0.983169697 | 0.840974819 |
| Role of CHK Proteins in Cell Cycle Checkpoint Control | 3.158990432 | 0 | 0 | 0 | 0.738131358 |
| Pyrimidine Ribonucleotides De Novo Biosynthesis | 2.065429271 | 0 | 0 | 0.964909048 | 0.823147936 |
| ILK Signaling | 0 | 0 | 2.830793311 | 0 | 0.786685681 |
| Role of Oct4 in Mammalian Embryonic Stem Cell Pluripotency | 0 | 0 | 0.980125389 | 2.231422528 | 0 |
| Salvage Pathways of Pyrimidine Ribonucleotides | 2.494118621 | 0 | 0 | 0.66725691 | 0 |
| Pyridoxal 5'-phosphate Salvage Pathway | 2.992489941 | 0 | 0 | 0 | 0 |
| GADD45 Signaling | 2.824501453 | 0 | 0 | 0 | 0 |
| Cyclins and Cell Cycle Regulation | 2.716716481 | 0 | 0 | 0 | 0 |
| Neuroprotective Role of THOP1 in Alzheimer's Disease | 0 | 0 | 0.600415718 | 0 | 2.053569475 |
| Glutamate Removal from Folates | 0 | 0 | 0 | 0 | 2.452238713 |
| Sonic Hedgehog Signaling | 2.428342739 | 0 | 0 | 0 | 0 |
| GDP-L-fucose Biosynthesis I (from GDP-D-mannose) | 0 | 0 | 0 | 2.303471637 | 0 |
| **N. PATHWAY CUT OFF ENRICHMENT SCORE [-Log(p value)] ≥ 2** | 13 | 0 | 1 | 2 | 3 |

1. SHARED TO ALL SUBTYPES (S) switch pathways enrichment score p value range 2.3E-14 – 8E-3 [↑](#footnote-ref-1)
2. SUBTYPE-SPECIFIC (SS) switch pathways enrichment score p value range 1E-3 – 5E-3 [↑](#footnote-ref-2)
