# Supplementary material for: The New Paradigm of Network Medicine to Analyze Breast Cancer Phenotypes
Source: Int J Mol Sci. 2020 Sep 12;21(18):6690. doi: 10.3390/ijms21186690 (PMC7555916; doi:10.3390/ijms21186690)
Supplement: Supplementary file 1 [file ijms-21-06690-s001.zip › Table S3.docx]

**Table S3.** Ingenuity Pathway analysis (IPA) heatmap for PAM50 stratification switch genes, related to Figure 4B.

| **PAM50 STRATIFICATION CANONICAL PATHWAYS** | **SHARED TO ALL SUBTYPES (S)^[[1]](#footnote-1)^** | **SUBTYPE-SPECIFIC (SS)^[[2]](#footnote-2)^** | | | |
| --- | --- | --- | --- | --- | --- |
|  |  | **Luminal A** | **Luminal B** | **HER2-enriched** | **Basal like** |
| Mitotic Roles of Polo-Like Kinase | 11.98761906 | 0 | 0 | 0 | 0.575631614 |
| Cell Cycle: G2/M DNA Damage Checkpoint Regulation | 9.955188982 | 0 | 0 | 0 | 0.688629574 |
| Cell Cycle Control of Chromosomal Replication | 2.561201605 | 0 | 0 | 0 | 1.550595394 |
| Atherosclerosis Signaling | 2.412953514 | 0 | 1.350846552 | 0 | 0.345765631 |
| DNA damage-induced 14-3-3σ Signaling | 3.959619869 | 0 | 0 | 0 | 0 |
| Salvage Pathways of Pyrimidine Ribonucleotides | 2.850316137 | 0 | 0 | 0.592407456 | 0.437662364 |
| Pyridoxal 5'-phosphate Salvage Pathway | 3.506468556 | 0 | 0 | 0 | 0 |
| Granulocyte Adhesion and Diapedesis | 2.691699841 | 0 | 0.424266414 | 0 | 0.245525436 |
| Estrogen-mediated S-phase Entry | 2.131817275 | 0 | 1.179877686 | 0 | 0 |
| CCR3 Signaling in Eosinophils | 0.297697475 | 0 | 2.225073542 | 0.459422534 | 0.3177039 |
| Agranulocyte Adhesion and Diapedesis | 2.559850423 | 0 | 0.400563327 | 0 | 0.22634362 |
| Role of CHK Proteins in Cell Cycle Checkpoint Control | 2.539282949 | 0 | 0 | 0 | 0.630671775 |
| Colanic Acid Building Blocks Biosynthesis | 0 | 0 | 0 | 3.094205938 | 0 |
| Pathogenesis of Multiple Sclerosis | 3.063436792 | 0 | 0 | 0 | 0 |
| Neuroinflammation Signaling Pathway | 0.33423809 | 0 | 2.036432368 | 0.610256642 | 0 |
| Cyclins and Cell Cycle Regulation | 2.111899838 | 0 | 0.716550474 | 0 | 0 |
| Netrin Signaling | 0 | 2.805648037 | 0 | 0 | 0 |
| Sorbitol Degradation I | 0 | 0 | 2.580905322 | 0 | 0 |
| UDP-N-acetyl-D-galactosamine Biosynthesis I | 0 | 0 | 0 | 2.518258513 | 0 |
| Relaxin Signaling | 0 | 0 | 2.043974121 | 0.40723545 | 0 |
| GADD45 Signaling | 2.400856959 | 0 | 0 | 0 | 0 |
| Glutamate Removal from Folates | 0 | 0 | 0 | 0 | 2.331496091 |
| GDP-L-fucose Biosynthesis I (from GDP-D-mannose) | 0 | 0 | 0 | 2.217877614 | 0 |
| IL-17A Signaling in Gastric Cells | 2.16517421 | 0 | 0 | 0 | 0 |
| Dermatan Sulfate Biosynthesis (Late Stages) | 0 | 0 | 0 | 2.065429271 | 0 |
| Chondroitin Sulfate Biosynthesis (Late Stages) | 0 | 0 | 0 | 2.012495329 | 0 |
| **N. PATHWAY CUT OFF ENRICHMENT SCORE [-Log(p value)] ≥ 2** | 15 | 1 | 4 | 5 | 1 |

1. SHARED TO ALL SUBTYPES switch pathways enrichment score p value range 1.03E-12 - 6.84E-3 [↑](#footnote-ref-1)
2. SUBTYPE-SPECIFIC (SS) switch pathways enrichment score p value range 8.05E-4 - 9.72E-3 [↑](#footnote-ref-2)
