# Supplementary material for: The New Paradigm of Network Medicine to Analyze Breast Cancer Phenotypes
Source: Int J Mol Sci. 2020 Sep 12;21(18):6690. doi: 10.3390/ijms21186690 (PMC7555916; doi:10.3390/ijms21186690)
Supplement: Supplementary file 1 [file ijms-21-06690-s001.zip › Table S4.docx]

**Table S4.** List of IHC shared switch enriched in statistically significant pathways and their IPA knowledge base annotations, related to figure 4A.

| **IHC shared switch (S) pathways** | **IHC shared switch genes^[[1]](#endnote-1)^** | **Gene stable ID** | **Gene description** | **HGNC ID** | **Location** | **Type(s)** |
| --- | --- | --- | --- | --- | --- | --- |
| Cell Cycle: G2/M DNA Damage Checkpoint Regulation | ***AURKA*** | ENSG00000087586 | aurora kinase A | 11393 | Nucleus | kinase |
|  | *CCNB1* | ENSG00000134057 | cyclin B1 | 1579 | Cytoplasm | kinase |
|  | *CCNB2* | ENSG00000157456 | cyclin B2 | 1580 | Cytoplasm | kinase |
|  | *CDC25C* | ENSG00000158402 | cell division cycle 25C | 1727 | Nucleus | phosphatase |
|  | *CDK1* | ENSG00000170312 | cyclin dependent kinase 1 | 1722 | Nucleus | kinase |
|  | ***CKS2*** | ENSG00000123975 | CDC28 protein kinase regulatory subunit 2 | 2000 | Other | kinase |
|  | *PKMYT1* | ENSG00000127564 | protein kinase, membrane associated tyrosine/threonine 1 | 29650 | Cytoplasm | kinase |
|  | *PLK1* | ENSG00000166851 | polo like kinase 1 | 9077 | Nucleus | kinase |
|  | *TOP2A* | ENSG00000131747 | DNA topoisomerase II alpha | 11989 | Nucleus | enzyme |
| Mitotic Roles of Polo-Like Kinase | *CCNB1* | ENSG00000134057 | cyclin B1 | 1579 | Cytoplasm | kinase |
|  | *CCNB2* | ENSG00000157456 | cyclin B2 | 1580 | Cytoplasm | kinase |
|  | ***CDC20*** | ENSG00000117399 | cell division cycle 20 | 1723 | Nucleus | other |
|  | *CDC25C* | ENSG00000158402 | cell division cycle 25C | 1727 | Nucleus | phosphatase |
|  | *CDK1* | ENSG00000170312 | cyclin dependent kinase 1 | 1722 | Nucleus | kinase |
|  | *PKMYT1* | ENSG00000127564 | protein kinase, membrane associated tyrosine/threonine 1 | 29650 | Cytoplasm | kinase |
|  | *PLK1* | ENSG00000166851 | polo like kinase 1 | 9077 | Nucleus | kinase |
|  | ***PTTG1*** | ENSG00000164611 | pituitary tumor-transforming 1 | 9690 | Nucleus | transcription regulator |
| Cell Cycle Control of Chromosomal Replication | ***CDC45*** | ENSG00000093009 | cell division cycle 45 | 1739 | Nucleus | other |
|  | *CDK1* | ENSG00000170312 | cyclin dependent kinase 1 | 1722 | Nucleus | kinase |
|  | ***CDT1*** | ENSG00000167513 | chromatin licensing and DNA replication factor 1 | 24576 | Nucleus | other |
|  | *TOP2A* | ENSG00000131747 | DNA topoisomerase II alpha | 11989 | Nucleus | enzyme |
| ATM Signaling | *CCNB1* | ENSG00000134057 | cyclin B1 | 1579 | Cytoplasm | kinase |
|  | *CCNB2* | ENSG00000157456 | cyclin B2 | 1580 | Cytoplasm | kinase |
|  | *CDC25C* | ENSG00000158402 | cell division cycle 25C | 1727 | Nucleus | phosphatase |
|  | *CDK1* | ENSG00000170312 | cyclin dependent kinase 1 | 1722 | Nucleus | kinase |
| DNA damage-induced 14-3-3σ Signaling | *CCNB1* | ENSG00000134057 | cyclin B1 | 1579 | Cytoplasm | kinase |
|  | *CCNB2* | ENSG00000157456 | cyclin B2 | 1580 | Cytoplasm | kinase |
|  | *CDK1* | ENSG00000170312 | cyclin dependent kinase 1 | 1722 | Nucleus | kinase |
| Pyrimidine Ribonucleotides Interconversion | *RAD54L* | ENSG00000085999 | RAD54 like | 9826 | Nucleus | enzyme |
|  | *RECQL4* | ENSG00000160957 | RecQ like helicase 4 | 9949 | Nucleus | enzyme |
| Role of CHK Proteins in Cell Cycle Checkpoint Control | *CDC25C* | ENSG00000158402 | cell division cycle 25C | 1727 | Nucleus | phosphatase |
|  | *CDK1* | ENSG00000170312 | cyclin dependent kinase 1 | 1722 | Nucleus | kinase |
|  | *PLK1* | ENSG00000166851 | polo like kinase 1 | 9077 | Nucleus | kinase |
| Pyrimidine Ribonucleotides De Novo Biosynthesis | *RAD54L* | ENSG00000085999 | RAD54 like | 9826 | Nucleus | enzyme |
|  | *RECQL4* | ENSG00000160957 | RecQ like helicase 4 | 9949 | Nucleus | enzyme |
| Salvage Pathways of Pyrimidine Ribonucleotides | *CDK1* | ENSG00000170312 | cyclin dependent kinase 1 | 1722 | Nucleus | kinase |
|  | *NEK2* | ENSG00000117650 | NIMA related kinase 2 | 7745 | Cytoplasm | kinase |
|  | *PLK1* | ENSG00000166851 | polo like kinase 1 | 9077 | Nucleus | kinase |
| Pyridoxal 5'-phosphate Salvage Pathway | *CDK1* | ENSG00000170312 | cyclin dependent kinase 1 | 1722 | Nucleus | kinase |
|  | *NEK2* | ENSG00000117650 | NIMA related kinase 2 | 7745 | Cytoplasm | kinase |
|  | *PLK1* | ENSG00000166851 | polo like kinase 1 | 9077 | Nucleus | kinase |
| GADD45 Signaling | *CCNB1* | ENSG00000134057 | cyclin B1 | 1579 | Cytoplasm | kinase |
|  | *CDK1* | ENSG00000170312 | cyclin dependent kinase 1 | 1722 | Nucleus | kinase |
| Cyclins and Cell Cycle Regulation | *CCNB1* | ENSG00000134057 | cyclin B1 | 1579 | Cytoplasm | kinase |
|  | *CCNB2* | ENSG00000157456 | cyclin B2 | 1580 | Cytoplasm | kinase |
|  | *CDK1* | ENSG00000170312 | cyclin dependent kinase 1 | 1722 | Nucleus | kinase |
| Sonic Hedgehog Signaling | *CCNB1* | ENSG00000134057 | cyclin B1 | 1579 | Cytoplasm | kinase |
|  | *CDK1* | ENSG00000170312 | cyclin dependent kinase 1 | 1722 | Nucleus | kinase |

1. Bold genes are involved in one pathway, all others are involved in more pathways. [↑](#endnote-ref-1)
