# Supplementary material for: The New Paradigm of Network Medicine to Analyze Breast Cancer Phenotypes
Source: Int J Mol Sci. 2020 Sep 12;21(18):6690. doi: 10.3390/ijms21186690 (PMC7555916; doi:10.3390/ijms21186690)
Supplement: Supplementary file 1 [file ijms-21-06690-s001.zip › Table S5.docx]

**Table S5*.*** List of IHC subtype specific switch enriched in statistically significant pathways and their IPA knowledge base annotations, related to Figure 4A.

| **IHC Subtypes** | **IHC subtype-specific (SS) switch pathways** | **IHC specific switch genes** | **Gene stable ID** | **Gene description** | **HGNC ID** | **Location** | **Type(s)** |
| --- | --- | --- | --- | --- | --- | --- | --- |
| Luminal HER2 negative | No statistically significant pathways^[[1]](#footnote-1)^ | ***-*** | **-** | **-** | **-** | **-** | **-** |
| Luminal B-like HER2 positive | ILK Signaling | *CREB3L4* | ENSG00000143578 | cAMP responsive element binding protein 3 like 4 | 18854 | Nucleus | transcription regulator |
|  |  | *FN1* | ENSG00000115414 | fibronectin 1 | 3778 | Extracellular Space | enzyme |
|  |  | *KRT18* | ENSG00000111057 | keratin 18 | 6430 | Cytoplasm | other |
|  |  | *MUC1* | ENSG00000185499 | mucin 1, cell surface associated | 7508 | Plasma Membrane | other |
| HER2 positive | Role of Oct4 in Mammalian Embryonic Stem Cell Pluripotency | *IGF2BP1* | ENSG00000159217 | insulin like growth factor 2 mRNA binding protein 1 | 28866 | Cytoplasm | translation regulator |
|  |  | *SALL4* | ENSG00000101115 | spalt like transcription factor 4 | 15924 | Nucleus | transcription regulator |
|  | GDP-L-fucose Biosynthesis I (from GDP-D-mannose) | *TSTA3* | ENSG00000278243 | tissue specific transplantation antigen P35B | 12390 | Plasma Membrane | enzyme |
| Triple negative | Cell Cycle Control of Chromosomal Replication^[[2]](#footnote-2)^ | *CDC7* | ENSG00000097046 | cell division cycle 7 | 1745 | Nucleus | kinase |
|  |  | *MCM2* | ENSG00000073111 | minichromosome maintenance complex component 2 | 6944 | Nucleus | enzyme |
|  |  | *MCM4* | ENSG00000104738 | minichromosome maintenance complex component 4 | 6947 | Nucleus | enzyme |
|  | Neuroprotective Role of THOP1 in Alzheimer's Disease | *MAPT* | ENSG00000277956 | microtubule associated protein tau | 6893 | Plasma Membrane | other |
|  |  | *PRSS27* | ENSG00000172382 | serine protease 27 | 15475 | Extracellular Space | peptidase |
|  |  | *TPSG1* | ENSG00000116176 | tryptase gamma 1 | 14134 | Extracellular Space | peptidase |
|  | Glutamate Removal from Folates | *GGH* | ENSG00000137563 | gamma-glutamyl hydrolase | 4248 | Cytoplasm | peptidase |

1. There are no statistically significant pathway to display because two switch genes for Lum HER 2 negative [↑](#footnote-ref-1)
2. The pathway “Cell cycle control of chromosal replication" has been found also for IHC shared switch genes. [↑](#footnote-ref-2)
