# Supplementary material for: The New Paradigm of Network Medicine to Analyze Breast Cancer Phenotypes
Source: Int J Mol Sci. 2020 Sep 12;21(18):6690. doi: 10.3390/ijms21186690 (PMC7555916; doi:10.3390/ijms21186690)
Supplement: Supplementary file 1 [file ijms-21-06690-s001.zip › Table S6.docx]

**Table S6.** List of PAM50 shared switch enriched in statistically significant pathways and their IPA knowledge base annotations, related to figure 4B.

| **PAM50 shared switch (S) pathways** | **PAM50 shared switch genes^[[1]](#endnote-1)^** | **Gene stable ID** | **Gene description** | **HGNC ID** | **Location** | **Type(s)** |
| --- | --- | --- | --- | --- | --- | --- |
| Mitotic Roles of Polo-Like Kinase | *CCNB2* | ENSG00000157456 | cyclin B2 | 1580 | Cytoplasm | kinase |
|  | ***CDC20*** | ENSG00000117399 | cell division cycle 20 | 1723 | Nucleus | other |
|  | *CDC25C* | ENSG00000158402 | cell division cycle 25C | 1727 | Nucleus | phosphatase |
|  | *CDK1* | ENSG00000170312 | cyclin dependent kinase 1 | 1722 | Nucleus | kinase |
|  | ***ESPL1*** | ENSG00000135476 | extra spindle pole bodies like 1, separase | 16856 | Nucleus | peptidase |
|  | ***KIF11*** | ENSG00000138160 | kinesin family member 11 | 6388 | Nucleus | other |
|  | ***KIF23*** | ENSG00000137807 | kinesin family member 23 | 6392 | Cytoplasm | other |
|  | *PKMYT1* | ENSG00000127564 | protein kinase, membrane associated tyrosine/threonine 1 | 29650 | Cytoplasm | kinase |
|  | *PLK1* | ENSG00000166851 | polo like kinase 1 | 9077 | Nucleus | kinase |
|  | ***PTTG1*** | ENSG00000164611 | pituitary tumor-transforming 1 | 9690 | Nucleus | transcription regulator |
| Cell Cycle: G2/M DNA Damage Checkpoint Regulation | ***AURKA*** | ENSG00000087586 | aurora kinase A | 11393 | Nucleus | kinase |
|  | *CCNB2* | ENSG00000157456 | cyclin B2 | 1580 | Cytoplasm | kinase |
|  | *CDC25C* | ENSG00000158402 | cell division cycle 25C | 1727 | Nucleus | phosphatase |
|  | *CDK1* | ENSG00000170312 | cyclin dependent kinase 1 | 1722 | Nucleus | kinase |
|  | ***CKS2*** | ENSG00000123975 | CDC28 protein kinase regulatory subunit 2 | 2000 | Other | kinase |
|  | *PKMYT1* | ENSG00000127564 | protein kinase, membrane associated tyrosine/threonine 1 | 29650 | Cytoplasm | kinase |
|  | *PLK1* | ENSG00000166851 | polo like kinase 1 | 9077 | Nucleus | kinase |
|  | *TOP2A* | ENSG00000131747 | DNA topoisomerase II alpha | 11989 | Nucleus | enzyme |
| Cell Cycle Control of Chromosomal Replication | ***CDC45*** | ENSG00000093009 | cell division cycle 45 | 1739 | Nucleus | other |
|  | *CDK1* | ENSG00000170312 | cyclin dependent kinase 1 | 1722 | Nucleus | kinase |
|  | *TOP2A* | ENSG00000131747 | DNA topoisomerase II alpha | 11989 | Nucleus | enzyme |
| Atherosclerosis Signaling | *CCL11* | ENSG00000172156 | C-C motif chemokine ligand 11 | 10610 | Extracellular Space | cytokine |
|  | ***COL10A1*** | ENSG00000123500 | collagen type X alpha 1 chain | 2185 | Extracellular Space | other |
|  | *MMP1* | ENSG00000196611 | matrix metallopeptidase 1 | 7155 | Extracellular Space | peptidase |
|  | ***PAFAH1B3*** | ENSG00000079462 | platelet activating factor acetylhydrolase 1b catalytic subunit 3 | 8576 | Cytoplasm | enzyme |
| DNA damage-induced 14-3-3σ Signaling | *CCNB2* | ENSG00000157456 | cyclin B2 | 1580 | Cytoplasm | kinase |
|  | *CCNE2* | ENSG00000175305 | cyclin E2 | 1590 | Nucleus | kinase |
|  | *CDK1* | ENSG00000170312 | cyclin dependent kinase 1 | 1722 | Nucleus | kinase |
| Salvage Pathways of Pyrimidine Ribonucleotides | *CDK1* | ENSG00000170312 | cyclin dependent kinase 1 | 1722 | Nucleus | kinase |
|  | *NEK2* | ENSG00000117650 | NIMA related kinase 2 | 7745 | Cytoplasm | kinase |
|  | *PLK1* | ENSG00000166851 | polo like kinase 1 | 9077 | Nucleus | kinase |
|  | *TTK* | ENSG00000112742 | TTK protein kinase | 12401 | Nucleus | kinase |
| Pyridoxal 5'-phosphate Salvage Pathway | *CDK1* | ENSG00000170312 | cyclin dependent kinase 1 | 1722 | Nucleus | kinase |
|  | *NEK2* | ENSG00000117650 | NIMA related kinase 2 | 7745 | Cytoplasm | kinase |
|  | *PLK1* | ENSG00000166851 | polo like kinase 1 | 9077 | Nucleus | kinase |
|  | *TTK* | ENSG00000112742 | TTK protein kinase | 12401 | Nucleus | kinase |
| Granulocyte Adhesion and Diapedesis | *CCL11* | ENSG00000172156 | C-C motif chemokine ligand 11 | 10610 | Extracellular Space | cytokine |
|  | *CXCL10* | ENSG00000169245 | C-X-C motif chemokine ligand 10 | 10637 | Extracellular Space | cytokine |
|  | *CXCL11* | ENSG00000169248 | C-X-C motif chemokine ligand 11 | 10638 | Extracellular Space | cytokine |
|  | *MMP1* | ENSG00000169245 | C-X-C motif chemokine ligand 10 | 10637 | Extracellular Space | cytokine |
|  | *MMP11* | ENSG00000169248 | C-X-C motif chemokine ligand 11 | 10638 | Extracellular Space | cytokine |
| Estrogen-mediated S-phase Entry | *CCNE2* | ENSG00000196611 | matrix metallopeptidase 1 | 7155 | Extracellular Space | peptidase |
|  | *CDK1* | ENSG00000099953 | matrix metallopeptidase 11 | 7157 | Extracellular Space | peptidase |
| Agranulocyte Adhesion and Diapedesis | *CCL11* | ENSG00000175305 | cyclin E2 | 1590 | Nucleus | kinase |
|  | *CXCL10* | ENSG00000170312 | cyclin dependent kinase 1 | 1722 | Nucleus | kinase |
|  | *CXCL11* | ENSG00000172156 | C-C motif chemokine ligand 11 | 10610 | Extracellular Space | cytokine |
|  | *MMP1* | ENSG00000169245 | C-X-C motif chemokine ligand 10 | 10637 | Extracellular Space | cytokine |
|  | *MMP11* | ENSG00000169248 | C-X-C motif chemokine ligand 11 | 10638 | Extracellular Space | cytokine |
| Role of CHK Proteins in Cell Cycle Checkpoint Control | *CDC25C* | ENSG00000196611 | matrix metallopeptidase 1 | 7155 | Extracellular Space | peptidase |
|  | *CDK1* | ENSG00000099953 | matrix metallopeptidase 11 | 7157 | Extracellular Space | peptidase |
|  | *PLK1* | ENSG00000158402 | cell division cycle 25C | 1727 | Nucleus | phosphatase |
| Pathogenesis of Multiple Sclerosis | *CXCL10* | ENSG00000170312 | cyclin dependent kinase 1 | 1722 | Nucleus | kinase |
|  | *CXCL11* | ENSG00000166851 | polo like kinase 1 | 9077 | Nucleus | kinase |
| Cyclins and Cell Cycle Regulation | *CCNB2* | ENSG00000169245 | C-X-C motif chemokine ligand 10 | 10637 | Extracellular Space | cytokine |
|  | *CCNE2* | ENSG00000169248 | C-X-C motif chemokine ligand 11 | 10638 | Extracellular Space | cytokine |
|  | *CDK1* | ENSG00000157456 | cyclin B2 | 1580 | Cytoplasm | kinase |
| GADD45 Signaling | *CCNE2* | ENSG00000175305 | cyclin E2 | 1590 | Nucleus | kinase |
|  | *CDK1* | ENSG00000170312 | cyclin dependent kinase 1 | 1722 | Nucleus | kinase |
| IL-17A Signaling in Gastric Cells | *CXCL10* | ENSG00000175305 | cyclin E2 | 1590 | Nucleus | kinase |
|  | *CXCL11* | ENSG00000170312 | cyclin dependent kinase 1 | 1722 | Nucleus | kinase |

1. Bold genes are involved in one pathway, all others are involved in more pathways. [↑](#endnote-ref-1)
