# Supplementary material for: The New Paradigm of Network Medicine to Analyze Breast Cancer Phenotypes
Source: Int J Mol Sci. 2020 Sep 12;21(18):6690. doi: 10.3390/ijms21186690 (PMC7555916; doi:10.3390/ijms21186690)
Supplement: Supplementary file 1 [file ijms-21-06690-s001.zip › Table S7.docx]

**Table S7.** List of PAM50 subtype specific switch enriched in statistically significant pathways and their IPA knowledge base annotations, related to Figure 4B.

| **PAM50 Subtypes** | **PAM50 subtype-specific (SS) switch pathways** | **PAM50 specific switch genes^[[1]](#footnote-1)^** | **Gene stable ID** | **Gene description** | **HGNC ID** | **Location** | **Type(s)** |
| --- | --- | --- | --- | --- | --- | --- | --- |
| Luminal A | Neutrin Signaling | ***CACNA1D*** | ENSG00000157388 | calcium voltage-gated channel subunit alpha1 D | 1391 | Plasma Membrane | ion channel |
|  |  | ***UNC5B*** | ENSG00000107731 | unc-5 netrin receptor B | 12568 | Plasma Membrane | transmembrane receptor |
| Luminal B | CCR3 Signaling in Eosinophils | *FGFR3* | ENSG00000068078 | fibroblast growth factor receptor 3 | 3690 | Plasma Membrane | kinase |
|  |  | *GNG13* | ENSG00000127588 | G protein subunit gamma 13 | 14131 | Plasma Membrane | enzyme |
|  |  | *PLA2G4F* | ENSG00000168907 | phospholipase A2 group IVF | 27396 | Cytoplasm | enzyme |
|  | Neuroinflammation Signaling Pathway | *FGFR3* | ENSG00000068078 | fibroblast growth factor receptor 3 | 3690 | Plasma Membrane | kinase |
|  |  | ***KCNJ3*** | ENSG00000162989 | potassium voltage-gated channel subfamily J member 3 | 6264 | Plasma Membrane | ion channel |
|  |  | *MMP9* | ENSG00000100985 | matrix metallopeptidase 9 | 7176 | Extracellular Space | peptidase |
|  |  | *PLA2G4F* | ENSG00000168907 | phospholipase A2 group IVF | 27396 | Cytoplasm | enzyme |
|  | Sorbitol Degradation I | ***SORD*** | ENSG00000140263 | sorbitol dehydrogenase | 11184 | Cytoplasm | enzyme |
|  | Relaxin Signaling | *FGFR3* | ENSG00000068078 | fibroblast growth factor receptor 3 | 3690 | Plasma Membrane | kinase |
|  |  | *GNG13* | ENSG00000127588 | G protein subunit gamma 13 | 14131 | Plasma Membrane | enzyme |
|  |  | *MMP9* | ENSG00000100985 | matrix metallopeptidase 9 | 7176 | Extracellular Space | peptidase |
| HER2 Positive | Colanic Acid Building Blocks Biosynthesis | *GALE* | ENSG00000117308 | UDP-galactose-4-epimerase | 4116 | Cytoplasm | enzyme |
|  |  | *TSTA3* | ENSG00000278243 | tissue specific transplantation antigen P35B | 12390 | Plasma Membrane | enzyme |
|  | UDP-N-acetyl-D-galactosamine Biosynthesis I | *GALE* | ENSG00000117308 | UDP-galactose-4-epimerase | 4116 | Cytoplasm | enzyme |
|  | GDP-L-fucose Biosynthesis I (from GDP-D-mannose) | *TSTA3* | ENSG00000278243 | tissue specific transplantation antigen P35B | 12390 | Plasma Membrane | enzyme |
|  | Dermatan Sulfate Biosynthesis (Late Stages) | *GAL3ST2* | ENSG00000154252 | galactose-3-O-sulfotransferase 2 | 24869 | Cytoplasm | enzyme |
|  |  | *HS6ST3* | ENSG00000185352 | heparan sulfate 6-O-sulfotransferase 3 | 19134 | Other | enzyme |
|  | Chondroitin Sulfate Biosynthesis (Late Stages) | *GAL3ST2* | ENSG00000154252 | galactose-3-O-sulfotransferase 2 | 24869 | Cytoplasm | enzyme |
|  |  | *HS6ST3* | ENSG00000185352 | heparan sulfate 6-O-sulfotransferase 3 | 19134 | Other | enzyme |
| Basal-like | Glutamate Removal from Folates | ***GGH*** | ENSG00000137563 | gamma-glutamyl hydrolase | 4248 | Cytoplasm | peptidase |

Bold genes are involved in one pathway, all others are involved in more pathways.

1. Bold genes are involved in one pathway, all others are involved in more pathways. [↑](#footnote-ref-1)
