# Supplementary material for: The New Paradigm of Network Medicine to Analyze Breast Cancer Phenotypes
Source: Int J Mol Sci. 2020 Sep 12;21(18):6690. doi: 10.3390/ijms21186690 (PMC7555916; doi:10.3390/ijms21186690)
Supplement: Supplementary file 1 [file ijms-21-06690-s001.zip › Table S8.docx]

**Table S8.** Unique S-switches between IHC and PAM50 classification, annotations and CancerMine database citations^[[1]](#footnote-1)^.

| **Gene stable ID** | **Gene name** | **Gene description** | **HGNC ID** | **driver_citations** | **oncogene_citations** | **tumor_suppressor_**  **citations** | **all_citations** |
| --- | --- | --- | --- | --- | --- | --- | --- |
| ENSG00000087586 | AURKA | aurora kinase A | 11393 | 2 | 20 | 2 | 24 |
| ENSG00000172156 | CCL11 | C-C motif chemokine ligand 11 | 10610 | #N/D | #N/D | #N/D | #N/D |
| ENSG00000134057 | CCNB1 | cyclin B1 | 1579 | 0 | 3 | 1 | 4 |
| ENSG00000157456 | CCNB2 | cyclin B2 | 1580 | 0 | 3 | 0 | 3 |
| ENSG00000175305 | CCNE2 | cyclin E2 | 1590 | 0 | 3 | 0 | 3 |
| ENSG00000117399 | CDC20 | cell division cycle 20 | 1723 | 2 | 16 | 0 | 18 |
| ENSG00000158402 | CDC25C | cell division cycle 25C | 1727 | #N/D | #N/D | #N/D | #N/D |
| ENSG00000093009 | CDC45 | cell division cycle 45 | 1739 | 0 | 1 | 0 | 1 |
| ENSG00000170312 | CDK1 | cyclin dependent kinase 1 | 1722 | 2 | 6 | 2 | 10 |
| ENSG00000167513 | CDT1 | chromatin licensing and DNA replication factor 1 | 24576 | #N/D | #N/D | #N/D | #N/D |
| ENSG00000123975 | CKS2 | CDC28 protein kinase regulatory subunit 2 | 2000 | 0 | 1 | 0 | 1 |
| ENSG00000123500 | COL10A1 | collagen type X alpha 1 chain | 2185 | #N/D | #N/D | #N/D | #N/D |
| ENSG00000169245 | CXCL10 | C-X-C motif chemokine ligand 10 | 10637 | 0 | 1 | 0 | 1 |
| ENSG00000169248 | CXCL11 | C-X-C motif chemokine ligand 11 | 10638 | 0 | 0 | 1 | 1 |
| ENSG00000135476 | ESPL1 | extra spindle pole bodies like 1, separase | 16856 | 0 | 3 | 0 | 3 |
| ENSG00000138160 | KIF11 | kinesin family member 11 | 6388 | 2 | 1 | 0 | 3 |
| ENSG00000137807 | KIF23 | kinesin family member 23 | 6392 | 0 | 1 | 0 | 1 |
| ENSG00000196611 | MMP1 | matrix metallopeptidase 1 | 7155 | 0 | 1 | 0 | 1 |
| ENSG00000275365 | MMP11 | matrix metallopeptidase 11 | 7157 | #N/D | #N/D | #N/D | #N/D |
| ENSG00000117650 | NEK2 | NIMA related kinase 2 | 7745 | 2 | 3 | 0 | 5 |
| ENSG00000079462 | PAFAH1B3 | platelet activating factor acetylhydrolase 1b catalytic subunit 3 | 8576 | 1 | 0 | 0 | 1 |
| ENSG00000127564 | PKMYT1 | protein kinase, membrane associated tyrosine/threonine 1 | 29650 | 0 | 1 | 0 | 1 |
| ENSG00000166851 | PLK1 | polo like kinase 1 | 9077 | 1 | 17 | 3 | 21 |
| ENSG00000164611 | PTTG1 | PTTG1 regulator of sister chromatid separation, securin | 9690 | 1 | 49 | 0 | 50 |
| ENSG00000085999 | RAD54L | RAD54 like | 9826 | #N/D | #N/D | #N/D | #N/D |
| ENSG00000160957 | RECQL4 | RecQ like helicase 4 | 9949 | 0 | 1 | 2 | 3 |
| ENSG00000131747 | TOP2A | DNA topoisomerase II alpha | 11989 | 1 | 6 | 0 | 7 |
| ENSG00000112742 | TTK | TTK protein kinase | 12401 | 1 | 3 | 0 | 4 |

1. #N/D: not defined in CancerMine Database [↑](#footnote-ref-1)
