# Supplementary material for: The New Paradigm of Network Medicine to Analyze Breast Cancer Phenotypes
Source: Int J Mol Sci. 2020 Sep 12;21(18):6690. doi: 10.3390/ijms21186690 (PMC7555916; doi:10.3390/ijms21186690)
Supplement: Supplementary file 1 [file ijms-21-06690-s001.zip › Table S9.docx]

**Table S9.** TCGA-BRCA clinicopathologic surrogate definition.

| *bcr_patient_barcode* | *er_status_by_ihc* | *er_status_ihc_Percent_Positive* | *pr_status_by_ihc* | *pr_status_ihc_percent_positive* | *her2_status_by_ihc* | *her2_ihc_percent_positive* | *her2_ihc_score* | *her2_fish_status* | *TCGA.BRCA.clinicopathologic.surrogate.definition.* |
| --- | --- | --- | --- | --- | --- | --- | --- | --- | --- |
| TCGA-3C-AAAU | Positive | 50-59% | Positive | 50-59% | Negative | [Not Available] | [Not Available] | [Not Evaluated] | Luminal-HER2 neg |
| TCGA-3C-AALI | Positive | <10% | Positive | <10% | Positive | [Not Available] | [Not Available] | [Not Evaluated] | Luminal B like (HER2 pos) |
| TCGA-3C-AALJ | Positive | 90-99% | Positive | 30-39% | Indeterminate | [Not Available] | [Not Available] | [Not Evaluated] | Undetermined |
| TCGA-3C-AALK | Positive | 70-79% | Positive | 80-89% | Positive | [Not Available] | [Not Available] | [Not Evaluated] | Luminal B like (HER2 pos) |
| TCGA-4H-AAAK | Positive | 60-69% | Positive | 70-79% | Equivocal | 10-19% | 2+ | [Not Evaluated] | Undetermined |
| TCGA-5L-AAT0 | Positive | 70-79% | Positive | 50-59% | Negative | [Not Available] | 1+ | [Not Evaluated] | Luminal-HER2 neg |
| TCGA-5L-AAT1 | Positive | 80-89% | Positive | 10-19% | Equivocal | [Not Available] | 2+ | [Not Evaluated] | Undetermined |
| TCGA-5T-A9QA | Positive | 70-79% | Negative | [Not Available] | Equivocal | 10-19% | 2+ | Negative | Luminal-HER2 neg |
| TCGA-A1-A0SB | Positive | 70-79% | Negative | [Not Available] | Negative | [Not Available] | [Not Available] | [Not Evaluated] | Luminal-HER2 neg |
| TCGA-A1-A0SD | Positive | 90-99% | Positive | 90-99% | Negative | [Not Available] | [Not Available] | [Not Evaluated] | Luminal-HER2 neg |
| TCGA-A1-A0SE | Positive | 80-89% | Positive | 90-99% | Negative | [Not Available] | 1+ | Negative | Luminal-HER2 neg |
| TCGA-A1-A0SF | Positive | 90-99% | Positive | 90-99% | Negative | [Not Available] | [Not Available] | [Not Evaluated] | Luminal-HER2 neg |
| TCGA-A1-A0SG | Positive | 90-99% | Positive | 90-99% | Negative | [Not Available] | [Not Available] | [Not Evaluated] | Luminal-HER2 neg |
| TCGA-A1-A0SH | Negative | [Not Available] | Positive | 90-99% | Equivocal | [Not Available] | 2+ | Negative | Luminal-HER2 neg |
| TCGA-A1-A0SI | Positive | 50-59% | Positive | 10-19% | Negative | [Not Available] | [Not Available] | [Not Evaluated] | Luminal-HER2 neg |
| TCGA-A1-A0SJ | Positive | 70-79% | Positive | 10-19% | Equivocal | [Not Available] | 2+ | Negative | Luminal-HER2 neg |
| TCGA-A1-A0SK | Negative | [Not Available] | Negative | [Not Available] | Negative | [Not Available] | 0 | [Not Evaluated] | Triple negative |
| TCGA-A1-A0SM | Positive | 20-29% | Negative | [Not Available] | Positive | [Not Available] | 3+ | Positive | Luminal B like (HER2 pos) |
| TCGA-A1-A0SN | Positive | 90-99% | Positive | 60-69% | Positive | [Not Available] | [Not Available] | [Not Evaluated] | Luminal B like (HER2 pos) |
| TCGA-A1-A0SO | Negative | [Not Available] | Negative | [Not Available] | Equivocal | [Not Available] | [Not Available] | Negative | Triple negative |
| TCGA-A1-A0SP | Negative | [Not Available] | Negative | [Not Available] | Negative | [Not Available] | 0 | [Not Evaluated] | Triple negative |
| TCGA-A1-A0SQ | Positive | 90-99% | Positive | 90-99% | Negative | [Not Available] | [Not Available] | [Not Evaluated] | Luminal-HER2 neg |
| TCGA-A2-A04N | Positive | [Not Available] | Positive | [Not Available] | [Not Evaluated] | [Not Available] | [Not Available] | Negative | Luminal-HER2 neg |
| TCGA-A2-A04P | Negative | <10% | Negative | <10% | [Not Evaluated] | [Not Available] | [Not Available] | Negative | Triple negative |
| TCGA-A2-A04Q | Negative | <10% | Negative | <10% | Equivocal | [Not Available] | 2+ | Negative | Triple negative |
| TCGA-A2-A04R | Positive | 60-69% | Positive | 80-89% | Equivocal | [Not Available] | 2+ | Negative | Luminal-HER2 neg |
| TCGA-A2-A04T | Negative | <10% | Negative | <10% | Equivocal | [Not Available] | 2+ | Negative | Triple negative |
| TCGA-A2-A04U | Negative | <10% | Negative | <10% | Negative | [Not Available] | 1+ | Positive | HER2-positive (non-luminal) |
| TCGA-A2-A04V | Positive | 90-99% | Positive | 10-19% | Equivocal | [Not Available] | 2+ | Negative | Luminal-HER2 neg |
| TCGA-A2-A04W | Negative | <10% | Negative | <10% | Positive | [Not Available] | [Not Available] | Positive | HER2-positive (non-luminal) |
| TCGA-A2-A04X | Positive | 80-89% | Positive | 50-59% | Positive | [Not Available] | 3+ | Positive | Luminal B like (HER2 pos) |
| TCGA-A2-A04Y | Positive | 90-99% | Positive | 10-19% | Negative | [Not Available] | 1+ | Negative | Luminal-HER2 neg |
| TCGA-A2-A0CK | Positive | 40-49% | Positive | 80-89% | Negative | [Not Available] | [Not Available] | Negative | Luminal-HER2 neg |
| TCGA-A2-A0CL | Positive | 20-29% | Positive | <10% | Negative | [Not Available] | 1+ | Negative | Luminal-HER2 neg |
| TCGA-A2-A0CM | Negative | <10% | Negative | <10% | Negative | <10% | 0 | [Not Evaluated] | Triple negative |
| TCGA-A2-A0CO | Positive | 40-49% | Positive | 20-29% | Negative | [Not Available] | [Not Available] | Negative | Luminal-HER2 neg |
| TCGA-A2-A0CP | Positive | 90-99% | Positive | 90-99% | [Not Evaluated] | [Not Available] | [Not Available] | Negative | Luminal-HER2 neg |
| TCGA-A2-A0CQ | Positive | 90-99% | Positive | 90-99% | Equivocal | [Not Available] | 2+ | Negative | Luminal-HER2 neg |
| TCGA-A2-A0CR | Positive | 60-69% | Positive | 80-89% | Negative | [Not Available] | [Not Available] | Negative | Luminal-HER2 neg |
| TCGA-A2-A0CS | Positive | 80-89% | Positive | 80-89% | Equivocal | [Not Available] | 2+ | Negative | Luminal-HER2 neg |
| TCGA-A2-A0CT | Positive | 90-99% | Negative | <10% | Equivocal | [Not Available] | 2+ | Negative | Luminal-HER2 neg |
| TCGA-A2-A0CU | Positive | 90-99% | Positive | <10% | Equivocal | [Not Available] | 2+ | Negative | Luminal-HER2 neg |
| TCGA-A2-A0CV | Positive | 90-99% | Positive | 90-99% | Negative | [Not Available] | 1+ | Negative | Luminal-HER2 neg |
| TCGA-A2-A0CW | Positive | 90-99% | Positive | 10-19% | Equivocal | [Not Available] | 2+ | Negative | Luminal-HER2 neg |
| TCGA-A2-A0CX | Positive | [Not Available] | Negative | <10% | Positive | [Not Available] | 3+ | [Not Evaluated] | Luminal B like (HER2 pos) |
| TCGA-A2-A0CY | Positive | 90-99% | Positive | 10-19% | Negative | [Not Available] | 1+ | Negative | Luminal-HER2 neg |
| TCGA-A2-A0CZ | Positive | 90-99% | Positive | 20-29% | Negative | [Not Available] | 1+ | Negative | Luminal-HER2 neg |
| TCGA-A2-A0D0 | Negative | <10% | Negative | <10% | Negative | [Not Available] | 0 | Negative | Triple negative |
| TCGA-A2-A0D1 | Negative | <10% | Negative | <10% | Positive | [Not Available] | 3+ | Positive | HER2-positive (non-luminal) |
| TCGA-A2-A0D2 | Negative | <10% | Negative | <10% | Negative | [Not Available] | 0 | Negative | Triple negative |
| TCGA-A2-A0D3 | Positive | 90-99% | Positive | 90-99% | Negative | [Not Available] | 1+ | Negative | Luminal-HER2 neg |
| TCGA-A2-A0D4 | Positive | 90-99% | Positive | 10-19% | Negative | [Not Available] | 1+ | Negative | Luminal-HER2 neg |
| TCGA-A2-A0EM | Positive | [Not Available] | Positive | [Not Available] | [Not Evaluated] | [Not Available] | [Not Available] | Negative | Luminal-HER2 neg |
| TCGA-A2-A0EN | Positive | [Not Available] | Positive | 50-59% | Equivocal | [Not Available] | 2+ | Negative | Luminal-HER2 neg |
| TCGA-A2-A0EO | Positive | [Not Available] | Positive | [Not Available] | Negative | [Not Available] | 1+ | Negative | Luminal-HER2 neg |
| TCGA-A2-A0EP | Positive | 80-89% | Negative | [Not Available] | Negative | [Not Available] | [Not Available] | Negative | Luminal-HER2 neg |
| TCGA-A2-A0EQ | Negative | <10% | Negative | <10% | Positive | [Not Available] | 3+ | Negative | Triple negative |
| TCGA-A2-A0ER | Positive | 90-99% | Positive | 80-89% | Equivocal | [Not Available] | 2+ | Negative | Luminal-HER2 neg |
| TCGA-A2-A0ES | Positive | 60-69% | Positive | 10-19% | Negative | [Not Available] | 1+ | Negative | Luminal-HER2 neg |
| TCGA-A2-A0ET | Positive | 90-99% | Positive | 70-79% | Equivocal | [Not Available] | 2+ | Negative | Luminal-HER2 neg |
| TCGA-A2-A0EU | Positive | 90-99% | Positive | 90-99% | Negative | [Not Available] | 0 | Negative | Luminal-HER2 neg |
| TCGA-A2-A0EV | Positive | 90-99% | Positive | 90-99% | Negative | <10% | 0 | Negative | Luminal-HER2 neg |
| TCGA-A2-A0EW | Positive | 90-99% | Positive | 10-19% | Negative | [Not Available] | 0 | Negative | Luminal-HER2 neg |
| TCGA-A2-A0EX | Positive | 60-69% | Positive | 90-99% | Negative | [Not Available] | 1+ | Negative | Luminal-HER2 neg |
| TCGA-A2-A0EY | Positive | 60-69% | Negative | <10% | Positive | [Not Available] | 3+ | Positive | Luminal B like (HER2 pos) |
| TCGA-A2-A0ST | Negative | [Not Available] | Negative | [Not Available] | [Not Evaluated] | [Not Available] | [Not Available] | Negative | Triple negative |
| TCGA-A2-A0SU | Positive | 90-99% | Positive | <10% | Negative | [Not Available] | 1+ | [Not Evaluated] | Luminal-HER2 neg |
| TCGA-A2-A0SV | Positive | 90-99% | Positive | 10-19% | Equivocal | [Not Available] | 2+ | Negative | Luminal-HER2 neg |
| TCGA-A2-A0SW | Positive | 40-49% | Negative | <10% | [Not Evaluated] | [Not Available] | [Not Available] | Negative | Luminal-HER2 neg |
| TCGA-A2-A0SX | Negative | [Not Available] | Negative | [Not Available] | Negative | [Not Available] | 1+ | [Not Evaluated] | Triple negative |
| TCGA-A2-A0SY | Positive | 80-89% | Positive | 40-49% | [Not Evaluated] | [Not Available] | [Not Available] | Positive | Luminal B like (HER2 pos) |
| TCGA-A2-A0T0 | Negative | <10% | Negative | <10% | Negative | <10% | 0 | [Not Evaluated] | Triple negative |
| TCGA-A2-A0T1 | Negative | <10% | Negative | <10% | Positive | [Not Available] | 3+ | [Not Evaluated] | HER2-positive (non-luminal) |
| TCGA-A2-A0T2 | Negative | <10% | Negative | <10% | Negative | [Not Available] | 0 | [Not Evaluated] | Triple negative |
| TCGA-A2-A0T3 | Positive | 90-99% | Positive | 90-99% | Equivocal | [Not Available] | 2+ | Negative | Luminal-HER2 neg |
| TCGA-A2-A0T4 | Positive | 90-99% | Positive | 60-69% | Equivocal | [Not Available] | 2+ | Negative | Luminal-HER2 neg |
| TCGA-A2-A0T5 | Positive | 90-99% | Positive | 90-99% | Negative | [Not Available] | 1+ | Negative | Luminal-HER2 neg |
| TCGA-A2-A0T6 | Positive | 90-99% | Positive | 90-99% | Negative | [Not Available] | 1+ | [Not Evaluated] | Luminal-HER2 neg |
| TCGA-A2-A0T7 | Positive | 90-99% | Positive | 70-79% | Negative | [Not Available] | 1+ | [Not Evaluated] | Luminal-HER2 neg |
| TCGA-A2-A0YC | Positive | 90-99% | Positive | 90-99% | [Not Evaluated] | [Not Available] | [Not Available] | Negative | Luminal-HER2 neg |
| TCGA-A2-A0YD | Positive | 90-99% | Positive | 80-89% | [Not Evaluated] | [Not Available] | [Not Available] | Negative | Luminal-HER2 neg |
| TCGA-A2-A0YE | Negative | <10% | Negative | <10% | Negative | <10% | 0 | [Not Evaluated] | Triple negative |
| TCGA-A2-A0YF | Positive | 90-99% | Negative | <10% | Negative | <10% | 0 | [Not Evaluated] | Luminal-HER2 neg |
| TCGA-A2-A0YG | Positive | 90-99% | Positive | <10% | Positive | [Not Available] | 3+ | [Not Evaluated] | Luminal B like (HER2 pos) |
| TCGA-A2-A0YH | Positive | 80-89% | Positive | 70-79% | Negative | <10% | 0 | [Not Evaluated] | Luminal-HER2 neg |
| TCGA-A2-A0YI | Positive | 90-99% | Positive | 60-69% | Negative | [Not Available] | 1+ | [Not Evaluated] | Luminal-HER2 neg |
| TCGA-A2-A0YJ | Positive | <10% | Negative | <10% | Negative | [Not Available] | 0 | [Not Evaluated] | Luminal-HER2 neg |
| TCGA-A2-A0YK | Positive | 90-99% | Negative | <10% | Negative | [Not Available] | 1+ | [Not Evaluated] | Luminal-HER2 neg |
| TCGA-A2-A0YL | Positive | 70-79% | Positive | 90-99% | Negative | [Not Available] | 1+ | [Not Evaluated] | Luminal-HER2 neg |
| TCGA-A2-A0YM | Negative | <10% | Negative | <10% | [Not Evaluated] | [Not Available] | [Not Available] | Negative | Triple negative |
| TCGA-A2-A0YT | Positive | 90-99% | Negative | <10% | [Not Evaluated] | [Not Available] | [Not Available] | Negative | Luminal-HER2 neg |
| TCGA-A2-A1FV | Positive | 90-99% | Positive | <10% | Negative | [Not Available] | 1+ | Negative | Luminal-HER2 neg |
| TCGA-A2-A1FW | Positive | 90-99% | Negative | [Not Available] | Negative | [Not Available] | [Not Available] | [Not Evaluated] | Luminal-HER2 neg |
| TCGA-A2-A1FX | Positive | 70-79% | Positive | 60-69% | [Not Evaluated] | [Not Available] | [Not Available] | Negative | Luminal-HER2 neg |
| TCGA-A2-A1FZ | Positive | 90-99% | Positive | 90-99% | Negative | [Not Available] | [Not Available] | [Not Evaluated] | Luminal-HER2 neg |
| TCGA-A2-A1G0 | Positive | 90-99% | Positive | 90-99% | Equivocal | [Not Available] | 2+ | Negative | Luminal-HER2 neg |
| TCGA-A2-A1G1 | Negative | [Not Available] | Negative | [Not Available] | Equivocal | [Not Available] | 2+ | Positive | HER2-positive (non-luminal) |
| TCGA-A2-A1G4 | Positive | 90-99% | Positive | 90-99% | Negative | [Not Available] | [Not Available] | [Not Evaluated] | Luminal-HER2 neg |
| TCGA-A2-A1G6 | Negative | [Not Available] | Negative | [Not Available] | Negative | [Not Available] | 1+ | [Not Evaluated] | Triple negative |
| TCGA-A2-A259 | Positive | 90-99% | Positive | 80-89% | Negative | [Not Available] | [Not Available] | Negative | Luminal-HER2 neg |
| TCGA-A2-A25A | Positive | <10% | Positive | <10% | Equivocal | [Not Available] | 2+ | Negative | Luminal-HER2 neg |
| TCGA-A2-A25B | Positive | 90-99% | Positive | 10-19% | Equivocal | [Not Available] | 2+ | Negative | Luminal-HER2 neg |
| TCGA-A2-A25C | Positive | 90-99% | Positive | 20-29% | Negative | [Not Available] | 1+ | [Not Evaluated] | Luminal-HER2 neg |
| TCGA-A2-A25D | Positive | 70-79% | Negative | [Not Available] | Negative | [Not Available] | 1+ | Negative | Luminal-HER2 neg |
| TCGA-A2-A25E | Positive | 80-89% | Positive | 60-69% | Equivocal | [Not Available] | 2+ | Equivocal | Undetermined |
| TCGA-A2-A25F | Negative | [Not Available] | Positive | <10% | Negative | <10% | [Not Available] | [Not Evaluated] | Luminal-HER2 neg |
| TCGA-A2-A3KC | Positive | 90-99% | Positive | 90-99% | Equivocal | [Not Available] | 2+ | Negative | Luminal-HER2 neg |
| TCGA-A2-A3KD | Positive | 90-99% | Positive | 70-79% | Negative | [Not Available] | 1+ | Negative | Luminal-HER2 neg |
| TCGA-A2-A3XS | Negative | [Not Available] | Negative | [Not Available] | [Not Evaluated] | [Not Available] | [Not Available] | Negative | Triple negative |
| TCGA-A2-A3XT | Negative | [Not Available] | Negative | [Not Available] | Negative | [Not Available] | [Not Available] | Negative | Triple negative |
| TCGA-A2-A3XU | Negative | [Not Available] | Negative | [Not Available] | [Not Evaluated] | [Not Available] | [Not Available] | Negative | Triple negative |
| TCGA-A2-A3XV | Positive | <10% | Negative | [Not Available] | Equivocal | [Not Available] | 2+ | Positive | Luminal B like (HER2 pos) |
| TCGA-A2-A3XW | Positive | 90-99% | Negative | [Not Available] | Negative | <10% | [Not Available] | Negative | Luminal-HER2 neg |
| TCGA-A2-A3XX | Negative | [Not Available] | Negative | [Not Available] | Negative | [Not Available] | 1+ | Negative | Triple negative |
| TCGA-A2-A3XY | Negative | [Not Available] | Negative | [Not Available] | Negative | [Not Available] | [Not Available] | Negative | Triple negative |
| TCGA-A2-A3XZ | Negative | [Not Available] | Negative | [Not Available] | Positive | [Not Available] | 3+ | [Not Evaluated] | HER2-positive (non-luminal) |
| TCGA-A2-A3Y0 | Positive | 90-99% | Negative | [Not Available] | Negative | [Not Available] | 1+ | [Not Evaluated] | Luminal-HER2 neg |
| TCGA-A2-A4RW | Positive | 80-89% | Positive | 90-99% | Negative | [Not Available] | 1+ | [Not Evaluated] | Luminal-HER2 neg |
| TCGA-A2-A4RX | Positive | <10% | Positive | <10% | Negative | [Not Available] | 1+ | [Not Evaluated] | Luminal-HER2 neg |
| TCGA-A2-A4RY | Positive | 90-99% | Positive | 90-99% | Negative | [Not Available] | 1+ | [Not Evaluated] | Luminal-HER2 neg |
| TCGA-A2-A4S0 | Positive | 90-99% | Positive | 90-99% | Negative | [Not Available] | 1+ | [Not Evaluated] | Luminal-HER2 neg |
| TCGA-A2-A4S1 | Positive | <10% | Negative | [Not Available] | Negative | [Not Available] | [Not Available] | [Not Evaluated] | Luminal-HER2 neg |
| TCGA-A2-A4S2 | Positive | 90-99% | Positive | 90-99% | Negative | [Not Available] | 1+ | [Not Evaluated] | Luminal-HER2 neg |
| TCGA-A2-A4S3 | Positive | 90-99% | Positive | 90-99% | Negative | [Not Available] | [Not Available] | [Not Evaluated] | Luminal-HER2 neg |
| TCGA-A7-A0CD | Positive | 90-99% | Positive | 70-79% | Equivocal | [Not Available] | 2+ | Negative | Luminal-HER2 neg |
| TCGA-A7-A0CE | Negative | <10% | Negative | <10% | Equivocal | [Not Available] | 2+ | Negative | Triple negative |
| TCGA-A7-A0CG | Positive | 90-99% | Negative | <10% | Negative | [Not Available] | 1+ | [Not Evaluated] | Luminal-HER2 neg |
| TCGA-A7-A0CH | [Not Evaluated] | [Not Available] | [Not Evaluated] | [Not Available] | [Not Evaluated] | [Not Available] | [Not Available] | [Not Evaluated] | Undetermined |
| TCGA-A7-A0CJ | Positive | 90-99% | Positive | 70-79% | Negative | <10% | 1+ | [Not Evaluated] | Luminal-HER2 neg |
| TCGA-A7-A0D9 | Positive | 90-99% | Negative | <10% | Negative | [Not Available] | 1+ | [Not Evaluated] | Luminal-HER2 neg |
| TCGA-A7-A0DA | Negative | [Not Available] | Negative | [Not Available] | Negative | [Not Available] | 1+ | [Not Evaluated] | Triple negative |
| TCGA-A7-A0DB | Positive | 90-99% | Positive | 60-69% | Negative | [Not Available] | 1+ | [Not Evaluated] | Luminal-HER2 neg |
| TCGA-A7-A0DC | Positive | 90-99% | Negative | <10% | Negative | [Not Available] | 1+ | [Not Evaluated] | Luminal-HER2 neg |
| TCGA-A7-A13D | Negative | <10% | Positive | <10% | Equivocal | [Not Available] | 2+ | Negative | Luminal-HER2 neg |
| TCGA-A7-A13E | Positive | 20-29% | Negative | <10% | Equivocal | [Not Available] | 2+ | Negative | Luminal-HER2 neg |
| TCGA-A7-A13F | Positive | [Not Available] | Positive | [Not Available] | Equivocal | [Not Available] | 2+ | Negative | Luminal-HER2 neg |
| TCGA-A7-A13G | Positive | 90-99% | Positive | 50-59% | Negative | [Not Available] | 1+ | [Not Evaluated] | Luminal-HER2 neg |
| TCGA-A7-A13H | Positive | 80-89% | Positive | 30-39% | Equivocal | [Not Available] | 2+ | Negative | Luminal-HER2 neg |
| TCGA-A7-A26E | Positive | 90-99% | Positive | 20-29% | Negative | [Not Available] | 1+ | [Not Evaluated] | Luminal-HER2 neg |
| TCGA-A7-A26F | Negative | [Not Available] | Negative | [Not Available] | Equivocal | [Not Available] | 2+ | Negative | Triple negative |
| TCGA-A7-A26G | Negative | [Not Available] | Negative | [Not Available] | Negative | [Not Available] | [Not Available] | [Not Evaluated] | Triple negative |
| TCGA-A7-A26H | Positive | 90-99% | Negative | [Not Available] | Equivocal | [Not Available] | 2+ | Positive | Luminal B like (HER2 pos) |
| TCGA-A7-A26I | Negative | [Not Available] | Negative | [Not Available] | Equivocal | [Not Available] | 2+ | Negative | Triple negative |
| TCGA-A7-A26J | Positive | 90-99% | Positive | 70-79% | Negative | [Not Available] | 1+ | [Not Evaluated] | Luminal-HER2 neg |
| TCGA-A7-A2KD | Positive | 90-99% | Positive | 90-99% | Positive | [Not Available] | 3+ | [Not Evaluated] | Luminal B like (HER2 pos) |
| TCGA-A7-A3IY | Positive | 90-99% | Positive | 90-99% | Negative | [Not Available] | [Not Available] | [Not Evaluated] | Luminal-HER2 neg |
| TCGA-A7-A3IZ | Positive | 90-99% | Negative | <10% | Equivocal | [Not Available] | 2+ | Negative | Luminal-HER2 neg |
| TCGA-A7-A3J0 | Positive | 90-99% | Positive | 90-99% | Negative | [Not Available] | 1+ | [Not Evaluated] | Luminal-HER2 neg |
| TCGA-A7-A3J1 | Positive | 90-99% | Positive | <10% | Negative | [Not Available] | [Not Available] | [Not Evaluated] | Luminal-HER2 neg |
| TCGA-A7-A3RF | Positive | 90-99% | Positive | 30-39% | Negative | [Not Available] | [Not Available] | [Not Evaluated] | Luminal-HER2 neg |
| TCGA-A7-A425 | Positive | 90-99% | Positive | 70-79% | Indeterminate | 30-39% | 3+ | Negative | Luminal-HER2 neg |
| TCGA-A7-A426 | Positive | 70-79% | Positive | 70-79% | Negative | <10% | [Not Available] | [Not Evaluated] | Luminal-HER2 neg |
| TCGA-A7-A4SA | Positive | 80-89% | Negative | [Not Available] | Negative | [Not Available] | 1+ | [Not Evaluated] | Luminal-HER2 neg |
| TCGA-A7-A4SB | Positive | 70-79% | Positive | 50-59% | Negative | [Not Available] | [Not Available] | [Not Evaluated] | Luminal-HER2 neg |
| TCGA-A7-A4SC | Positive | 90-99% | Negative | [Not Available] | Equivocal | [Not Available] | 3+ | Negative | Luminal-HER2 neg |
| TCGA-A7-A4SD | Negative | [Not Available] | Negative | [Not Available] | Equivocal | [Not Available] | 2+ | Negative | Triple negative |
| TCGA-A7-A4SE | Negative | [Not Available] | Negative | [Not Available] | Negative | <10% | 1+ | [Not Evaluated] | Triple negative |
| TCGA-A7-A4SF | Positive | 90-99% | Negative | [Not Available] | Positive | [Not Available] | 3+ | [Not Evaluated] | Luminal B like (HER2 pos) |
| TCGA-A7-A56D | Positive | 90-99% | Positive | <10% | Negative | [Not Available] | [Not Available] | [Not Evaluated] | Luminal-HER2 neg |
| TCGA-A7-A5ZV | Negative | [Not Available] | Negative | [Not Available] | Equivocal | [Not Available] | 2+ | Negative | Triple negative |
| TCGA-A7-A5ZW | Positive | 90-99% | Positive | 80-89% | Negative | [Not Available] | 1+ | [Not Evaluated] | Luminal-HER2 neg |
| TCGA-A7-A5ZX | Positive | [Not Available] | Positive | [Not Available] | Negative | [Not Available] | [Not Available] | [Not Evaluated] | Luminal-HER2 neg |
| TCGA-A7-A6VV | Negative | [Not Available] | Negative | [Not Available] | Negative | [Not Available] | [Not Available] | [Not Evaluated] | Triple negative |
| TCGA-A7-A6VW | Negative | [Not Available] | Negative | [Not Available] | Negative | [Not Available] | [Not Available] | [Not Evaluated] | Triple negative |
| TCGA-A7-A6VX | Positive | 90-99% | Positive | [Not Available] | Negative | [Not Available] | 1+ | [Not Evaluated] | Luminal-HER2 neg |
| TCGA-A7-A6VY | Negative | [Not Available] | Negative | [Not Available] | Negative | [Not Available] | [Not Available] | [Not Evaluated] | Triple negative |
| TCGA-A8-A06N | Positive | [Not Available] | Negative | [Not Available] | Negative | [Not Available] | 0 | Negative | Luminal-HER2 neg |
| TCGA-A8-A06O | Positive | [Not Available] | Positive | [Not Available] | Negative | [Not Available] | 1+ | Negative | Luminal-HER2 neg |
| TCGA-A8-A06P | Positive | [Not Available] | Positive | [Not Available] | Negative | [Not Available] | [Not Available] | Negative | Luminal-HER2 neg |
| TCGA-A8-A06Q | Positive | [Not Available] | Positive | [Not Available] | Negative | [Not Available] | 1+ | Negative | Luminal-HER2 neg |
| TCGA-A8-A06R | Positive | [Not Available] | Negative | [Not Available] | Positive | [Not Available] | 2+ | Positive | Luminal B like (HER2 pos) |
| TCGA-A8-A06T | Positive | [Not Available] | Positive | [Not Available] | Positive | [Not Available] | 2+ | Positive | Luminal B like (HER2 pos) |
| TCGA-A8-A06U | Positive | [Not Available] | Positive | [Not Available] | Positive | [Not Available] | 2+ | Positive | Luminal B like (HER2 pos) |
| TCGA-A8-A06X | Positive | [Not Available] | Negative | [Not Available] | Positive | [Not Available] | 3+ | Positive | Luminal B like (HER2 pos) |
| TCGA-A8-A06Y | Positive | [Not Available] | Positive | [Not Available] | Negative | [Not Available] | 1+ | Negative | Luminal-HER2 neg |
| TCGA-A8-A06Z | Positive | [Not Available] | Positive | [Not Available] | Negative | [Not Available] | 1+ | Negative | Luminal-HER2 neg |
| TCGA-A8-A075 | Positive | [Not Available] | Positive | [Not Available] | Positive | [Not Available] | 2+ | Positive | Luminal B like (HER2 pos) |
| TCGA-A8-A076 | Positive | [Not Available] | Positive | [Not Available] | Positive | [Not Available] | 3+ | Positive | Luminal B like (HER2 pos) |
| TCGA-A8-A079 | Positive | [Not Available] | Positive | [Not Available] | Negative | [Not Available] | 1+ | Negative | Luminal-HER2 neg |
| TCGA-A8-A07B | Positive | [Not Available] | Positive | [Not Available] | Positive | [Not Available] | 3+ | Positive | Luminal B like (HER2 pos) |
| TCGA-A8-A07C | Negative | [Not Available] | Negative | [Not Available] | Negative | [Not Available] | 1+ | Negative | Triple negative |
| TCGA-A8-A07E | Positive | [Not Available] | Positive | [Not Available] | Negative | [Not Available] | 1+ | Negative | Luminal-HER2 neg |
| TCGA-A8-A07F | Positive | [Not Available] | Positive | [Not Available] | Negative | [Not Available] | 1+ | Negative | Luminal-HER2 neg |
| TCGA-A8-A07G | Positive | [Not Available] | Positive | [Not Available] | Negative | [Not Available] | 1+ | Negative | Luminal-HER2 neg |
| TCGA-A8-A07I | Positive | [Not Available] | Negative | [Not Available] | Positive | [Not Available] | 3+ | Positive | Luminal B like (HER2 pos) |
| TCGA-A8-A07J | Positive | [Not Available] | Positive | [Not Available] | Negative | [Not Available] | 1+ | Negative | Luminal-HER2 neg |
| TCGA-A8-A07L | Positive | [Not Available] | Positive | [Not Available] | Negative | [Not Available] | 0 | Negative | Luminal-HER2 neg |
| TCGA-A8-A07O | Negative | [Not Available] | Negative | [Not Available] | Negative | [Not Available] | 1+ | Negative | Triple negative |
| TCGA-A8-A07P | Positive | [Not Available] | Positive | [Not Available] | Positive | [Not Available] | 2+ | Positive | Luminal B like (HER2 pos) |
| TCGA-A8-A07R | Negative | [Not Available] | Negative | [Not Available] | Positive | [Not Available] | 2+ | [Not Available] | HER2-positive (non-luminal) |
| TCGA-A8-A07S | Positive | [Not Available] | Negative | [Not Available] | Negative | [Not Available] | [Not Available] | Negative | Luminal-HER2 neg |
| TCGA-A8-A07U | Negative | [Not Available] | Positive | [Not Available] | Negative | [Not Available] | [Not Available] | Negative | Luminal-HER2 neg |
| TCGA-A8-A07W | Positive | [Not Available] | Positive | [Not Available] | Negative | [Not Available] | [Not Available] | Negative | Luminal-HER2 neg |
| TCGA-A8-A07Z | Positive | [Not Available] | Positive | [Not Available] | Negative | [Not Available] | [Not Available] | Negative | Luminal-HER2 neg |
| TCGA-A8-A081 | Positive | [Not Available] | Positive | [Not Available] | Negative | [Not Available] | 1+ | Negative | Luminal-HER2 neg |
| TCGA-A8-A082 | Positive | [Not Available] | Positive | [Not Available] | Negative | [Not Available] | 1+ | Negative | Luminal-HER2 neg |
| TCGA-A8-A083 | Positive | [Not Available] | Positive | [Not Available] | Negative | [Not Available] | 1+ | Negative | Luminal-HER2 neg |
| TCGA-A8-A084 | Positive | [Not Available] | Negative | [Not Available] | Negative | [Not Available] | 1+ | Negative | Luminal-HER2 neg |
| TCGA-A8-A085 | Positive | [Not Available] | Positive | [Not Available] | Negative | [Not Available] | 1+ | Negative | Luminal-HER2 neg |
| TCGA-A8-A086 | Positive | [Not Available] | Positive | [Not Available] | Negative | [Not Available] | 0 | Negative | Luminal-HER2 neg |
| TCGA-A8-A08A | Positive | [Not Available] | Positive | [Not Available] | Negative | [Not Available] | 1+ | Negative | Luminal-HER2 neg |
| TCGA-A8-A08B | Positive | [Not Available] | Negative | [Not Available] | Positive | [Not Available] | 3+ | Positive | Luminal B like (HER2 pos) |
| TCGA-A8-A08C | Positive | [Not Available] | Positive | [Not Available] | Positive | [Not Available] | 2+ | Positive | Luminal B like (HER2 pos) |
| TCGA-A8-A08F | Positive | [Not Available] | Positive | [Not Available] | Negative | [Not Available] | 1+ | Negative | Luminal-HER2 neg |
| TCGA-A8-A08G | Positive | [Not Available] | Positive | [Not Available] | Positive | [Not Available] | 3+ | Positive | Luminal B like (HER2 pos) |
| TCGA-A8-A08H | Positive | [Not Available] | Positive | [Not Available] | Positive | [Not Available] | 3+ | Positive | Luminal B like (HER2 pos) |
| TCGA-A8-A08I | Positive | [Not Available] | Positive | [Not Available] | Negative | [Not Available] | 1+ | Negative | Luminal-HER2 neg |
| TCGA-A8-A08J | Positive | [Not Available] | Negative | [Not Available] | Negative | [Not Available] | 1+ | Negative | Luminal-HER2 neg |
| TCGA-A8-A08L | Positive | [Not Available] | Negative | [Not Available] | Negative | [Not Available] | 1+ | Negative | Luminal-HER2 neg |
| TCGA-A8-A08O | Positive | [Not Available] | Positive | [Not Available] | Negative | [Not Available] | 1+ | Negative | Luminal-HER2 neg |
| TCGA-A8-A08P | Positive | [Not Available] | Positive | [Not Available] | Positive | [Not Available] | 3+ | Positive | Luminal B like (HER2 pos) |
| TCGA-A8-A08R | Negative | [Not Available] | Negative | [Not Available] | Negative | [Not Available] | 1+ | Negative | Triple negative |
| TCGA-A8-A08S | Positive | [Not Available] | Positive | [Not Available] | Positive | [Not Available] | 3+ | Positive | Luminal B like (HER2 pos) |
| TCGA-A8-A08T | Positive | [Not Available] | Positive | [Not Available] | Positive | [Not Available] | 2+ | Positive | Luminal B like (HER2 pos) |
| TCGA-A8-A08X | Negative | [Not Available] | Negative | [Not Available] | Positive | [Not Available] | 3+ | Positive | HER2-positive (non-luminal) |
| TCGA-A8-A08Z | Positive | [Not Available] | Positive | [Not Available] | Negative | [Not Available] | 1+ | Negative | Luminal-HER2 neg |
| TCGA-A8-A090 | Positive | [Not Available] | Positive | [Not Available] | Positive | [Not Available] | 2+ | Positive | Luminal B like (HER2 pos) |
| TCGA-A8-A091 | Positive | [Not Available] | Negative | [Not Available] | Negative | [Not Available] | 1+ | Negative | Luminal-HER2 neg |
| TCGA-A8-A092 | Positive | [Not Available] | Positive | [Not Available] | Negative | [Not Available] | 1+ | Negative | Luminal-HER2 neg |
| TCGA-A8-A093 | Positive | [Not Available] | Positive | [Not Available] | Negative | [Not Available] | 1+ | Negative | Luminal-HER2 neg |
| TCGA-A8-A094 | Positive | [Not Available] | Negative | [Not Available] | Negative | [Not Available] | 1+ | Negative | Luminal-HER2 neg |
| TCGA-A8-A095 | Positive | [Not Available] | Positive | [Not Available] | Negative | [Not Available] | 1+ | Negative | Luminal-HER2 neg |
| TCGA-A8-A096 | Positive | [Not Available] | Positive | [Not Available] | Negative | [Not Available] | 1+ | Negative | Luminal-HER2 neg |
| TCGA-A8-A097 | Positive | [Not Available] | Positive | [Not Available] | Positive | [Not Available] | 2+ | Positive | Luminal B like (HER2 pos) |
| TCGA-A8-A099 | Positive | [Not Available] | Positive | [Not Available] | Positive | [Not Available] | 3+ | Positive | Luminal B like (HER2 pos) |
| TCGA-A8-A09A | Positive | [Not Available] | Positive | [Not Available] | Negative | [Not Available] | 1+ | Negative | Luminal-HER2 neg |
| TCGA-A8-A09B | Positive | [Not Available] | Positive | [Not Available] | Negative | [Not Available] | [Not Available] | Negative | Luminal-HER2 neg |
| TCGA-A8-A09C | Positive | [Not Available] | Positive | [Not Available] | Negative | [Not Available] | 0 | Negative | Luminal-HER2 neg |
| TCGA-A8-A09D | Positive | [Not Available] | Positive | [Not Available] | Negative | [Not Available] | 1+ | Negative | Luminal-HER2 neg |
| TCGA-A8-A09E | Positive | [Not Available] | Positive | [Not Available] | Positive | [Not Available] | 2+ | Positive | Luminal B like (HER2 pos) |
| TCGA-A8-A09G | Positive | [Not Available] | Negative | [Not Available] | Positive | [Not Available] | 2+ | Positive | Luminal B like (HER2 pos) |
| TCGA-A8-A09I | Positive | [Not Available] | Positive | [Not Available] | Positive | [Not Available] | 3+ | Positive | Luminal B like (HER2 pos) |
| TCGA-A8-A09K | Positive | [Not Available] | Positive | [Not Available] | Negative | [Not Available] | 0 | Negative | Luminal-HER2 neg |
| TCGA-A8-A09M | Positive | [Not Available] | Positive | [Not Available] | Negative | [Not Available] | 1+ | Negative | Luminal-HER2 neg |
| TCGA-A8-A09N | Positive | [Not Available] | Positive | [Not Available] | Positive | [Not Available] | 3+ | Positive | Luminal B like (HER2 pos) |
| TCGA-A8-A09Q | Positive | [Not Available] | Positive | [Not Available] | Negative | [Not Available] | [Not Available] | Negative | Luminal-HER2 neg |
| TCGA-A8-A09R | Positive | [Not Available] | Positive | [Not Available] | Negative | [Not Available] | 1+ | Negative | Luminal-HER2 neg |
| TCGA-A8-A09T | Positive | [Not Available] | Positive | [Not Available] | Negative | [Not Available] | [Not Available] | Negative | Luminal-HER2 neg |
| TCGA-A8-A09V | Positive | [Not Available] | Positive | [Not Available] | Negative | [Not Available] | 0 | Negative | Luminal-HER2 neg |
| TCGA-A8-A09W | Positive | [Not Available] | Positive | [Not Available] | Negative | [Not Available] | 0 | Negative | Luminal-HER2 neg |
| TCGA-A8-A09X | Negative | [Not Available] | Negative | [Not Available] | Negative | [Not Available] | 1+ | Negative | Triple negative |
| TCGA-A8-A09Z | Positive | [Not Available] | Negative | [Not Available] | Negative | [Not Available] | 1+ | Negative | Luminal-HER2 neg |
| TCGA-A8-A0A1 | Positive | [Not Available] | Positive | [Not Available] | Negative | [Not Available] | [Not Available] | Negative | Luminal-HER2 neg |
| TCGA-A8-A0A2 | Positive | [Not Available] | Positive | [Not Available] | Negative | [Not Available] | 0 | Negative | Luminal-HER2 neg |
| TCGA-A8-A0A4 | Positive | [Not Available] | Positive | [Not Available] | Negative | [Not Available] | [Not Available] | Negative | Luminal-HER2 neg |
| TCGA-A8-A0A6 | Positive | [Not Available] | Positive | [Not Available] | Negative | [Not Available] | 1+ | Negative | Luminal-HER2 neg |
| TCGA-A8-A0A7 | Negative | [Not Available] | Negative | [Not Available] | Positive | [Not Available] | 3+ | Positive | HER2-positive (non-luminal) |
| TCGA-A8-A0A9 | Positive | [Not Available] | Positive | [Not Available] | Negative | [Not Available] | [Not Available] | Negative | Luminal-HER2 neg |
| TCGA-A8-A0AB | Positive | [Not Available] | Positive | [Not Available] | Positive | [Not Available] | 2+ | Positive | Luminal B like (HER2 pos) |
| TCGA-A8-A0AD | Positive | [Not Available] | Positive | [Not Available] | Negative | [Not Available] | 1+ | Negative | Luminal-HER2 neg |
| TCGA-AC-A23C | Positive | 90-99% | Positive | 50-59% | Positive | [Not Available] | [Not Available] | Positive | Luminal B like (HER2 pos) |
| TCGA-AC-A23E | Positive | 30-39% | Positive | 80-89% | [Not Evaluated] | [Not Available] | [Not Available] | [Not Evaluated] | Undetermined |
| TCGA-AC-A23G | Positive | 90-99% | Positive | 80-89% | Positive | [Not Available] | [Not Available] | Positive | Luminal B like (HER2 pos) |
| TCGA-AC-A23H | Positive | 10-19% | Negative | [Not Available] | Positive | [Not Available] | [Not Available] | Positive | Luminal B like (HER2 pos) |
| TCGA-AC-A2B8 | Positive | 90-99% | Positive | 90-99% | Negative | [Not Available] | [Not Available] | Negative | Luminal-HER2 neg |
| TCGA-AC-A2BK | Negative | [Not Available] | Negative | [Not Available] | Negative | [Not Available] | [Not Available] | [Not Evaluated] | Triple negative |
| TCGA-AC-A2BM | Positive | 60-69% | Positive | 10-19% | [Not Evaluated] | [Not Available] | [Not Available] | Negative | Luminal-HER2 neg |
| TCGA-AC-A2FB | Positive | 90-99% | Positive | <10% | Positive | 10-19% | 3+ | [Not Evaluated] | Luminal B like (HER2 pos) |
| TCGA-AC-A2FF | Positive | 30-39% | Positive | 70-79% | [Not Evaluated] | [Not Available] | [Not Available] | Negative | Luminal-HER2 neg |
| TCGA-AC-A2FG | Positive | 90-99% | Negative | [Not Available] | [Not Evaluated] | [Not Available] | [Not Available] | Negative | Luminal-HER2 neg |
| TCGA-AC-A2FK | Positive | 90-99% | Positive | 90-99% | [Not Evaluated] | [Not Available] | [Not Available] | Negative | Luminal-HER2 neg |
| TCGA-AC-A2FM | [Not Evaluated] | [Not Available] | [Not Evaluated] | [Not Available] | [Not Evaluated] | [Not Available] | [Not Available] | [Not Evaluated] | Undetermined |
| TCGA-AC-A2FO | Positive | 90-99% | Negative | [Not Available] | Negative | [Not Available] | [Not Available] | Indeterminate | Luminal-HER2 neg |
| TCGA-AC-A2QH | Negative | [Not Available] | Negative | [Not Available] | Negative | [Not Available] | [Not Available] | [Not Evaluated] | Triple negative |
| TCGA-AC-A2QI | Positive | 90-99% | Negative | [Not Available] | Negative | [Not Available] | 1+ | [Not Evaluated] | Luminal-HER2 neg |
| TCGA-AC-A2QJ | Negative | [Not Available] | Negative | [Not Available] | Negative | [Not Available] | [Not Available] | [Not Evaluated] | Triple negative |
| TCGA-AC-A3BB | Positive | 90-99% | Positive | 90-99% | Negative | [Not Available] | [Not Available] | [Not Evaluated] | Luminal-HER2 neg |
| TCGA-AC-A3HN | Positive | 90-99% | Positive | <10% | Negative | <10% | [Not Available] | [Not Evaluated] | Luminal-HER2 neg |
| TCGA-AC-A3OD | Positive | 90-99% | Positive | 90-99% | Negative | [Not Available] | 1+ | [Not Evaluated] | Luminal-HER2 neg |
| TCGA-AC-A3QP | Positive | 70-79% | Positive | 90-99% | Equivocal | [Not Available] | 2+ | Negative | Luminal-HER2 neg |
| TCGA-AC-A3TN | Positive | 90-99% | Positive | 80-89% | Positive | [Not Available] | [Not Available] | Indeterminate | Luminal B like (HER2 pos) |
| TCGA-AC-A3W5 | Positive | 50-59% | Positive | 30-39% | [Not Evaluated] | [Not Available] | [Not Available] | Positive | Luminal B like (HER2 pos) |
| TCGA-AC-A3W6 | Positive | 90-99% | Positive | 20-29% | Negative | [Not Available] | 3+ | [Not Evaluated] | Luminal-HER2 neg |
| TCGA-AC-A3YI | Positive | 90-99% | Positive | 20-29% | Positive | [Not Available] | 1+ | [Not Evaluated] | Luminal B like (HER2 pos) |
| TCGA-AC-A3YJ | Positive | [Not Available] | Positive | [Not Available] | [Not Available] | [Not Available] | [Not Available] | [Not Available] | Undetermined |
| TCGA-AC-A5XS | Positive | [Not Available] | Positive | [Not Available] | Negative | [Not Available] | [Not Available] | [Not Evaluated] | Luminal-HER2 neg |
| TCGA-AC-A62V | Positive | 90-99% | Positive | <10% | [Not Evaluated] | [Not Available] | [Not Available] | [Not Evaluated] | Undetermined |
| TCGA-AC-A62X | Positive | <10% | Negative | [Not Available] | [Not Evaluated] | [Not Available] | [Not Available] | [Not Evaluated] | Undetermined |
| TCGA-AC-A62Y | Positive | 90-99% | Positive | 80-89% | Negative | [Not Available] | 1+ | [Not Evaluated] | Luminal-HER2 neg |
| TCGA-AC-A6IV | Positive | 90-99% | Positive | 60-69% | Equivocal | [Not Available] | 2+ | [Not Evaluated] | Undetermined |
| TCGA-AC-A6IW | Negative | <10% | Negative | <10% | Negative | [Not Available] | [Not Available] | [Not Evaluated] | Triple negative |
| TCGA-AC-A6IX | Positive | 90-99% | Positive | <10% | [Not Evaluated] | [Not Available] | [Not Available] | [Not Evaluated] | Undetermined |
| TCGA-AC-A6NO | Positive | [Not Available] | Positive | [Not Available] | Negative | [Not Available] | [Not Available] | [Not Evaluated] | Luminal-HER2 neg |
| TCGA-AC-A7VB | Positive | 80-89% | Negative | <10% | [Not Evaluated] | [Not Available] | [Not Available] | [Not Evaluated] | Undetermined |
| TCGA-AC-A7VC | Negative | [Not Available] | Negative | [Not Available] | Negative | [Not Available] | [Not Available] | [Not Evaluated] | Triple negative |
| TCGA-AC-A8OP | Positive | 90-99% | Positive | 10-19% | Indeterminate | [Not Available] | 1+ | [Not Evaluated] | Undetermined |
| TCGA-AC-A8OQ | Negative | [Not Available] | Negative | [Not Available] | Indeterminate | [Not Available] | [Not Available] | [Not Evaluated] | Undetermined |
| TCGA-AC-A8OR | Positive | 90-99% | Positive | 90-99% | Negative | <10% | [Not Available] | [Not Evaluated] | Luminal-HER2 neg |
| TCGA-AC-A8OS | Positive | 90-99% | Positive | <10% | Positive | 10-19% | 1+ | [Not Evaluated] | Luminal B like (HER2 pos) |
| TCGA-AN-A03X | Positive | [Not Available] | Positive | [Not Available] | Positive | [Not Available] | 1+ | [Not Evaluated] | Luminal B like (HER2 pos) |
| TCGA-AN-A03Y | Positive | [Not Available] | Positive | [Not Available] | Negative | [Not Available] | 0 | [Not Evaluated] | Luminal-HER2 neg |
| TCGA-AN-A041 | Positive | [Not Available] | Negative | [Not Available] | Positive | [Not Available] | 2+ | [Not Evaluated] | Luminal B like (HER2 pos) |
| TCGA-AN-A046 | Positive | [Not Available] | Positive | [Not Available] | Negative | [Not Available] | [Not Available] | [Not Evaluated] | Luminal-HER2 neg |
| TCGA-AN-A049 | Positive | [Not Available] | Positive | [Not Available] | Negative | [Not Available] | 0 | [Not Evaluated] | Luminal-HER2 neg |
| TCGA-AN-A04A | Positive | [Not Available] | Positive | [Not Available] | Negative | [Not Available] | 0 | [Not Evaluated] | Luminal-HER2 neg |
| TCGA-AN-A04C | Negative | [Not Available] | Negative | [Not Available] | Positive | [Not Available] | 3+ | [Not Evaluated] | HER2-positive (non-luminal) |
| TCGA-AN-A04D | Negative | [Not Available] | Negative | [Not Available] | Negative | [Not Available] | 0 | [Not Evaluated] | Triple negative |
| TCGA-AN-A0AJ | Positive | 80-89% | Positive | 40-49% | Positive | 90-99% | 3+ | [Not Evaluated] | Luminal B like (HER2 pos) |
| TCGA-AN-A0AK | Positive | 80-89% | Negative | <10% | Positive | 70-79% | 2+ | [Not Evaluated] | Luminal B like (HER2 pos) |
| TCGA-AN-A0AL | Negative | <10% | Negative | <10% | Negative | <10% | 0 | [Not Evaluated] | Triple negative |
| TCGA-AN-A0AM | Positive | [Not Available] | Negative | [Not Available] | Negative | [Not Available] | [Not Available] | [Not Evaluated] | Luminal-HER2 neg |
| TCGA-AN-A0AR | Negative | [Not Available] | Negative | [Not Available] | Negative | [Not Available] | 0 | [Not Evaluated] | Triple negative |
| TCGA-AN-A0AS | Positive | [Not Available] | Negative | [Not Available] | Negative | [Not Available] | 0 | [Not Evaluated] | Luminal-HER2 neg |
| TCGA-AN-A0AT | Negative | [Not Available] | Negative | [Not Available] | Negative | [Not Available] | [Not Available] | [Not Evaluated] | Triple negative |
| TCGA-AN-A0FD | Positive | 80-89% | Positive | 10-19% | Positive | 60-69% | 2+ | Positive | Luminal B like (HER2 pos) |
| TCGA-AN-A0FF | Positive | 90-99% | Positive | 90-99% | Negative | <10% | 0 | [Not Evaluated] | Luminal-HER2 neg |
| TCGA-AN-A0FJ | Positive | 10-19% | Negative | <10% | Positive | 40-49% | 1+ | [Not Evaluated] | Luminal B like (HER2 pos) |
| TCGA-AN-A0FK | Positive | 90-99% | Positive | 90-99% | Positive | 20-29% | 1+ | [Not Evaluated] | Luminal B like (HER2 pos) |
| TCGA-AN-A0FL | Negative | <10% | Negative | <10% | Positive | 20-29% | 1+ | [Not Evaluated] | HER2-positive (non-luminal) |
| TCGA-AN-A0FN | Positive | 90-99% | Positive | 10-19% | Positive | 20-29% | 1+ | [Not Evaluated] | Luminal B like (HER2 pos) |
| TCGA-AN-A0FS | Positive | 90-99% | Negative | <10% | Positive | 20-29% | 1+ | [Not Evaluated] | Luminal B like (HER2 pos) |
| TCGA-AN-A0FT | Positive | 90-99% | Positive | 90-99% | Positive | 90-99% | 3+ | [Not Evaluated] | Luminal B like (HER2 pos) |
| TCGA-AN-A0FV | Negative | [Not Available] | Negative | [Not Available] | Positive | [Not Available] | 3+ | [Not Evaluated] | HER2-positive (non-luminal) |
| TCGA-AN-A0FW | Positive | [Not Available] | Indeterminate | [Not Available] | Negative | [Not Available] | 1+ | [Not Evaluated] | Luminal-HER2 neg |
| TCGA-AN-A0FX | Negative | [Not Available] | Negative | [Not Available] | Positive | [Not Available] | 1+ | [Not Evaluated] | HER2-positive (non-luminal) |
| TCGA-AN-A0FY | Positive | [Not Available] | Positive | [Not Available] | Negative | [Not Available] | 0 | [Not Evaluated] | Luminal-HER2 neg |
| TCGA-AN-A0FZ | Positive | [Not Available] | Negative | [Not Available] | Positive | [Not Available] | 2+ | [Not Evaluated] | Luminal B like (HER2 pos) |
| TCGA-AN-A0G0 | Negative | [Not Available] | Negative | [Not Available] | Negative | [Not Available] | 1+ | [Not Evaluated] | Triple negative |
| TCGA-AN-A0XL | Positive | [Not Available] | Positive | [Not Available] | Negative | [Not Available] | 1+ | [Not Evaluated] | Luminal-HER2 neg |
| TCGA-AN-A0XN | Negative | [Not Available] | Positive | [Not Available] | Negative | [Not Available] | [Not Available] | [Not Evaluated] | Luminal-HER2 neg |
| TCGA-AN-A0XO | Positive | [Not Available] | Negative | [Not Available] | Negative | [Not Available] | 1+ | [Not Evaluated] | Luminal-HER2 neg |
| TCGA-AN-A0XP | Positive | [Not Available] | Positive | [Not Available] | Positive | [Not Available] | 2+ | [Not Evaluated] | Luminal B like (HER2 pos) |
| TCGA-AN-A0XR | Positive | [Not Available] | Negative | [Not Available] | Negative | [Not Available] | [Not Available] | [Not Evaluated] | Luminal-HER2 neg |
| TCGA-AN-A0XS | Negative | [Not Available] | Positive | [Not Available] | Negative | [Not Available] | [Not Available] | [Not Evaluated] | Luminal-HER2 neg |
| TCGA-AN-A0XT | Positive | [Not Available] | Negative | [Not Available] | Negative | [Not Available] | [Not Available] | [Not Evaluated] | Luminal-HER2 neg |
| TCGA-AN-A0XU | Negative | [Not Available] | Negative | [Not Available] | Negative | [Not Available] | [Not Available] | [Not Evaluated] | Triple negative |
| TCGA-AN-A0XV | Positive | 90-99% | Positive | 30-39% | Positive | 60-69% | 2+ | Negative | Luminal-HER2 neg |
| TCGA-AN-A0XW | Positive | 90-99% | Positive | 90-99% | Positive | 60-69% | 2+ | Negative | Luminal-HER2 neg |
| TCGA-AO-A03L | Positive | 70-79% | Positive | 10-19% | Positive | 10-19% | 2+ | Negative | Luminal-HER2 neg |
| TCGA-AO-A03M | Positive | 70-79% | Positive | 60-69% | Negative | 30-39% | 2+ | Negative | Luminal-HER2 neg |
| TCGA-AO-A03N | Positive | 70-79% | Positive | <10% | Negative | <10% | 0 | [Not Evaluated] | Luminal-HER2 neg |
| TCGA-AO-A03O | Positive | [Not Available] | Positive | [Not Available] | Negative | <10% | 1+ | Negative | Luminal-HER2 neg |
| TCGA-AO-A03P | Positive | 90-99% | Positive | 40-49% | Negative | <10% | 0 | [Not Evaluated] | Luminal-HER2 neg |
| TCGA-AO-A03R | Positive | 90-99% | Positive | <10% | Negative | <10% | 0 | Negative | Luminal-HER2 neg |
| TCGA-AO-A03T | Positive | 90-99% | Positive | 60-69% | Negative | <10% | 0 | [Not Evaluated] | Luminal-HER2 neg |
| TCGA-AO-A03U | Negative | <10% | Negative | <10% | Negative | <10% | 0 | Negative | Triple negative |
| TCGA-AO-A03V | Positive | 90-99% | Positive | 80-89% | Negative | <10% | 0 | [Not Evaluated] | Luminal-HER2 neg |
| TCGA-AO-A0J2 | Negative | <10% | Negative | <10% | Equivocal | 30-39% | 2+ | Negative | Triple negative |
| TCGA-AO-A0J3 | Positive | <10% | Positive | <10% | Equivocal | 10-19% | 2+ | Negative | Luminal-HER2 neg |
| TCGA-AO-A0J4 | Negative | <10% | Negative | <10% | Negative | <10% | [Not Available] | [Not Evaluated] | Triple negative |
| TCGA-AO-A0J5 | Positive | 90-99% | Negative | <10% | Equivocal | 10-19% | 2+ | Negative | Luminal-HER2 neg |
| TCGA-AO-A0J6 | Negative | <10% | Negative | <10% | Negative | <10% | 0 | [Not Evaluated] | Triple negative |
| TCGA-AO-A0J7 | Positive | 90-99% | Positive | <10% | Negative | <10% | 1+ | [Not Evaluated] | Luminal-HER2 neg |
| TCGA-AO-A0J8 | Positive | 90-99% | Positive | 70-79% | Negative | <10% | 1+ | [Not Evaluated] | Luminal-HER2 neg |
| TCGA-AO-A0J9 | Positive | 90-99% | Positive | 30-39% | [Not Evaluated] | [Not Available] | [Not Available] | Negative | Luminal-HER2 neg |
| TCGA-AO-A0JA | Positive | 90-99% | Positive | <10% | Negative | <10% | 1+ | [Not Evaluated] | Luminal-HER2 neg |
| TCGA-AO-A0JB | Positive | 90-99% | Positive | 60-69% | Negative | <10% | 0 | [Not Evaluated] | Luminal-HER2 neg |
| TCGA-AO-A0JC | Positive | 90-99% | Positive | 50-59% | Negative | <10% | 0 | [Not Evaluated] | Luminal-HER2 neg |
| TCGA-AO-A0JD | Positive | 90-99% | Positive | 40-49% | Negative | <10% | 1+ | Negative | Luminal-HER2 neg |
| TCGA-AO-A0JE | Negative | <10% | Negative | <10% | Positive | 50-59% | 3+ | Positive | HER2-positive (non-luminal) |
| TCGA-AO-A0JF | Positive | 90-99% | Positive | 70-79% | Negative | <10% | 1+ | [Not Evaluated] | Luminal-HER2 neg |
| TCGA-AO-A0JG | Positive | 90-99% | Positive | 10-19% | Negative | <10% | 1+ | [Not Evaluated] | Luminal-HER2 neg |
| TCGA-AO-A0JI | Positive | 90-99% | Negative | <10% | Negative | <10% | 1+ | [Not Evaluated] | Luminal-HER2 neg |
| TCGA-AO-A0JJ | Positive | 90-99% | Positive | 70-79% | Negative | <10% | 0 | [Not Evaluated] | Luminal-HER2 neg |
| TCGA-AO-A0JL | Negative | <10% | Negative | <10% | Negative | <10% | 1+ | Positive | HER2-positive (non-luminal) |
| TCGA-AO-A0JM | Positive | 90-99% | Positive | <10% | Positive | 70-79% | 3+ | Positive | Luminal B like (HER2 pos) |
| TCGA-AO-A124 | Negative | <10% | Negative | <10% | Negative | <10% | 0 | [Not Evaluated] | Triple negative |
| TCGA-AO-A125 | Positive | 50-59% | Positive | 90-99% | Negative | <10% | 0 | Negative | Luminal-HER2 neg |
| TCGA-AO-A126 | Positive | 10-19% | Positive | 10-19% | Negative | <10% | 1+ | Negative | Luminal-HER2 neg |
| TCGA-AO-A128 | Negative | <10% | Negative | <10% | Negative | <10% | 0 | Negative | Triple negative |
| TCGA-AO-A129 | Negative | <10% | Negative | <10% | Negative | <10% | 0 | Negative | Triple negative |
| TCGA-AO-A12A | Positive | 90-99% | Positive | 90-99% | Negative | <10% | 1+ | Negative | Luminal-HER2 neg |
| TCGA-AO-A12B | Positive | 90-99% | Positive | 90-99% | Negative | <10% | 0 | [Not Evaluated] | Luminal-HER2 neg |
| TCGA-AO-A12C | Positive | 90-99% | Positive | 40-49% | Positive | 90-99% | 3+ | Negative | Luminal-HER2 neg |
| TCGA-AO-A12D | Negative | <10% | Negative | <10% | Positive | 90-99% | 3+ | Positive | HER2-positive (non-luminal) |
| TCGA-AO-A12E | Positive | 80-89% | Positive | 40-49% | Negative | <10% | 0 | [Not Evaluated] | Luminal-HER2 neg |
| TCGA-AO-A12F | Negative | <10% | Negative | <10% | Negative | <10% | 1+ | [Not Evaluated] | Triple negative |
| TCGA-AO-A12G | Positive | 90-99% | Positive | 30-39% | Positive | [Not Available] | 2+ | Negative | Luminal-HER2 neg |
| TCGA-AO-A12H | Positive | 90-99% | Positive | <10% | Negative | <10% | 0 | [Not Evaluated] | Luminal-HER2 neg |
| TCGA-AO-A1KO | Positive | 90-99% | Positive | 90-99% | Negative | <10% | 1+ | [Not Evaluated] | Luminal-HER2 neg |
| TCGA-AO-A1KP | Positive | 80-89% | Positive | 40-49% | Negative | <10% | 1+ | Negative | Luminal-HER2 neg |
| TCGA-AO-A1KQ | Positive | 90-99% | Positive | 80-89% | Negative | <10% | 1+ | [Not Evaluated] | Luminal-HER2 neg |
| TCGA-AO-A1KR | Negative | [Not Available] | Negative | [Not Available] | Negative | [Not Available] | 1+ | Negative | Triple negative |
| TCGA-AO-A1KS | Positive | 90-99% | Positive | 90-99% | Equivocal | 10-19% | 2+ | Negative | Luminal-HER2 neg |
| TCGA-AO-A1KT | Positive | 90-99% | Positive | 40-49% | Equivocal | 10-19% | 2+ | Negative | Luminal-HER2 neg |
| TCGA-AQ-A04H | Positive | 90-99% | Positive | 20-29% | Indeterminate | [Not Available] | 2+ | Positive | Luminal B like (HER2 pos) |
| TCGA-AQ-A04J | Negative | <10% | Negative | <10% | Negative | <10% | 0 | [Not Evaluated] | Triple negative |
| TCGA-AQ-A04L | Positive | 60-69% | Negative | [Not Available] | Positive | [Not Available] | 3+ | [Not Evaluated] | Luminal B like (HER2 pos) |
| TCGA-AQ-A0Y5 | Positive | 70-79% | Positive | 40-49% | Positive | [Not Available] | 2+ | Positive | Luminal B like (HER2 pos) |
| TCGA-AQ-A1H2 | Positive | 90-99% | Positive | 10-19% | Indeterminate | [Not Available] | 2+ | Positive | Luminal B like (HER2 pos) |
| TCGA-AQ-A1H3 | Positive | 90-99% | Positive | 80-89% | Negative | [Not Available] | 1+ | [Not Evaluated] | Luminal-HER2 neg |
| TCGA-AQ-A54N | Negative | [Not Available] | Negative | [Not Available] | Equivocal | [Not Available] | 2+ | Negative | Triple negative |
| TCGA-AQ-A54O | Positive | 90-99% | Positive | 70-79% | Negative | [Not Available] | 1+ | [Not Evaluated] | Luminal-HER2 neg |
| TCGA-AQ-A7U7 | Positive | 80-89% | Positive | 80-89% | Negative | [Not Available] | [Not Available] | [Not Evaluated] | Luminal-HER2 neg |
| TCGA-AR-A0TP | Positive | <10% | Negative | [Not Available] | Negative | [Not Available] | [Not Available] | [Not Evaluated] | Luminal-HER2 neg |
| TCGA-AR-A0TQ | Positive | 10-19% | Negative | [Not Available] | Equivocal | [Not Available] | 2+ | Positive | Luminal B like (HER2 pos) |
| TCGA-AR-A0TR | Positive | [Not Available] | Positive | [Not Available] | Equivocal | [Not Available] | 2+ | Negative | Luminal-HER2 neg |
| TCGA-AR-A0TS | Negative | [Not Available] | Negative | [Not Available] | Negative | [Not Available] | 1+ | [Not Evaluated] | Triple negative |
| TCGA-AR-A0TT | Positive | 10-19% | Negative | [Not Available] | Negative | [Not Available] | 1+ | [Not Evaluated] | Luminal-HER2 neg |
| TCGA-AR-A0TU | Negative | [Not Available] | Negative | [Not Available] | Negative | [Not Available] | 1+ | [Not Evaluated] | Triple negative |
| TCGA-AR-A0TV | Positive | 90-99% | Positive | [Not Available] | Equivocal | [Not Available] | 2+ | Negative | Luminal-HER2 neg |
| TCGA-AR-A0TW | Positive | [Not Available] | Positive | [Not Available] | Equivocal | [Not Available] | 2+ | Negative | Luminal-HER2 neg |
| TCGA-AR-A0TX | Positive | <10% | Positive | [Not Available] | Positive | [Not Available] | 3+ | [Not Evaluated] | Luminal B like (HER2 pos) |
| TCGA-AR-A0TY | Positive | 10-19% | Negative | [Not Available] | Negative | [Not Available] | [Not Available] | [Not Evaluated] | Luminal-HER2 neg |
| TCGA-AR-A0TZ | Positive | [Not Available] | Positive | [Not Available] | Equivocal | [Not Available] | 2+ | Negative | Luminal-HER2 neg |
| TCGA-AR-A0U0 | Negative | [Not Available] | Negative | [Not Available] | [Not Evaluated] | [Not Available] | [Not Available] | [Not Evaluated] | Undetermined |
| TCGA-AR-A0U1 | Negative | [Not Available] | Negative | [Not Available] | Negative | [Not Available] | 1+ | [Not Available] | Triple negative |
| TCGA-AR-A0U2 | Positive | 10-19% | Positive | [Not Available] | Negative | [Not Available] | 1+ | [Not Evaluated] | Luminal-HER2 neg |
| TCGA-AR-A0U3 | Positive | 10-19% | Positive | [Not Available] | Negative | [Not Available] | 1+ | [Not Evaluated] | Luminal-HER2 neg |
| TCGA-AR-A0U4 | Negative | [Not Available] | Negative | [Not Available] | Negative | [Not Available] | [Not Available] | [Not Evaluated] | Triple negative |
| TCGA-AR-A1AH | Positive | 10-19% | Negative | [Not Available] | Negative | [Not Available] | [Not Available] | [Not Evaluated] | Luminal-HER2 neg |
| TCGA-AR-A1AI | Negative | [Not Available] | Negative | [Not Available] | Equivocal | [Not Available] | 2+ | Negative | Triple negative |
| TCGA-AR-A1AJ | Positive | 10-19% | Negative | [Not Available] | Negative | [Not Available] | [Not Available] | [Not Evaluated] | Luminal-HER2 neg |
| TCGA-AR-A1AK | Positive | 10-19% | Positive | 10-19% | Negative | [Not Available] | 1+ | [Not Evaluated] | Luminal-HER2 neg |
| TCGA-AR-A1AL | Positive | 70-79% | Positive | 70-79% | Negative | [Not Available] | 1+ | [Not Evaluated] | Luminal-HER2 neg |
| TCGA-AR-A1AM | Positive | [Not Available] | Positive | [Not Available] | Equivocal | [Not Available] | 2+ | Negative | Luminal-HER2 neg |
| TCGA-AR-A1AN | Positive | 70-79% | Positive | 70-79% | Negative | [Not Available] | [Not Available] | [Not Evaluated] | Luminal-HER2 neg |
| TCGA-AR-A1AO | Positive | [Not Available] | Negative | [Not Available] | Negative | [Not Available] | 1+ | [Not Evaluated] | Luminal-HER2 neg |
| TCGA-AR-A1AP | Positive | 70-79% | Positive | <10% | Equivocal | [Not Available] | 2+ | Negative | Luminal-HER2 neg |
| TCGA-AR-A1AQ | Negative | [Not Available] | Negative | [Not Available] | Equivocal | [Not Available] | 2+ | Negative | Triple negative |
| TCGA-AR-A1AR | Negative | [Not Available] | Negative | [Not Available] | Negative | [Not Available] | [Not Available] | [Not Evaluated] | Triple negative |
| TCGA-AR-A1AS | Positive | 70-79% | Positive | 70-79% | Negative | [Not Available] | 1+ | [Not Evaluated] | Luminal-HER2 neg |
| TCGA-AR-A1AT | Positive | <10% | Positive | <10% | Positive | [Not Available] | 3+ | [Not Evaluated] | Luminal B like (HER2 pos) |
| TCGA-AR-A1AU | Positive | 70-79% | Positive | 70-79% | Equivocal | [Not Available] | 2+ | Negative | Luminal-HER2 neg |
| TCGA-AR-A1AV | Positive | 70-79% | Positive | 70-79% | Negative | [Not Available] | 1+ | [Not Evaluated] | Luminal-HER2 neg |
| TCGA-AR-A1AW | Positive | 70-79% | Positive | 70-79% | Equivocal | [Not Available] | 2+ | Negative | Luminal-HER2 neg |
| TCGA-AR-A1AX | Positive | [Not Available] | Positive | 70-79% | Positive | [Not Available] | 3+ | [Not Evaluated] | Luminal B like (HER2 pos) |
| TCGA-AR-A1AY | Negative | [Not Available] | Negative | [Not Available] | Negative | [Not Available] | [Not Available] | [Not Evaluated] | Triple negative |
| TCGA-AR-A24H | Positive | [Not Available] | Positive | [Not Available] | Negative | [Not Available] | 1+ | [Not Evaluated] | Luminal-HER2 neg |
| TCGA-AR-A24K | Positive | 10-19% | Positive | 10-19% | Equivocal | [Not Available] | 2+ | Negative | Luminal-HER2 neg |
| TCGA-AR-A24L | Positive | 10-19% | Positive | 10-19% | Equivocal | [Not Available] | 2+ | Negative | Luminal-HER2 neg |
| TCGA-AR-A24M | Positive | 10-19% | Positive | 10-19% | Negative | [Not Available] | 1+ | [Not Evaluated] | Luminal-HER2 neg |
| TCGA-AR-A24N | Positive | 10-19% | Positive | 10-19% | Equivocal | [Not Available] | 2+ | Negative | Luminal-HER2 neg |
| TCGA-AR-A24O | Positive | [Not Available] | Positive | [Not Available] | Negative | [Not Available] | [Not Available] | [Not Evaluated] | Luminal-HER2 neg |
| TCGA-AR-A24P | Positive | 10-19% | Positive | 10-19% | Equivocal | [Not Available] | 2+ | Negative | Luminal-HER2 neg |
| TCGA-AR-A24Q | Positive | <10% | Negative | [Not Available] | Negative | [Not Available] | [Not Available] | [Not Evaluated] | Luminal-HER2 neg |
| TCGA-AR-A24R | Positive | 10-19% | Positive | 10-19% | Negative | [Not Available] | 1+ | Negative | Luminal-HER2 neg |
| TCGA-AR-A24S | Positive | 70-79% | Positive | [Not Available] | Negative | [Not Available] | [Not Available] | [Not Evaluated] | Luminal-HER2 neg |
| TCGA-AR-A24T | Positive | 10-19% | Positive | 10-19% | Equivocal | [Not Available] | 2+ | Negative | Luminal-HER2 neg |
| TCGA-AR-A24U | Negative | [Not Available] | Negative | [Not Available] | Positive | [Not Available] | 3+ | [Not Evaluated] | HER2-positive (non-luminal) |
| TCGA-AR-A24V | Positive | 80-89% | Positive | <10% | Negative | [Not Available] | [Not Available] | [Not Evaluated] | Luminal-HER2 neg |
| TCGA-AR-A24W | Positive | [Not Available] | Positive | [Not Available] | Negative | [Not Available] | [Not Available] | [Not Evaluated] | Luminal-HER2 neg |
| TCGA-AR-A24X | Positive | 80-89% | Positive | 90-99% | Equivocal | [Not Available] | 2+ | Negative | Luminal-HER2 neg |
| TCGA-AR-A24Z | Positive | [Not Available] | Positive | [Not Available] | Negative | [Not Available] | [Not Available] | [Not Evaluated] | Luminal-HER2 neg |
| TCGA-AR-A250 | Positive | 70-79% | Negative | [Not Available] | Equivocal | [Not Available] | 2+ | Positive | Luminal B like (HER2 pos) |
| TCGA-AR-A251 | Positive | [Not Available] | Negative | [Not Available] | Equivocal | [Not Available] | 2+ | Negative | Luminal-HER2 neg |
| TCGA-AR-A252 | Positive | 70-79% | Positive | [Not Available] | Negative | [Not Available] | [Not Available] | [Not Evaluated] | Luminal-HER2 neg |
| TCGA-AR-A254 | Positive | [Not Available] | Positive | [Not Available] | Positive | [Not Available] | 3+ | [Not Evaluated] | Luminal B like (HER2 pos) |
| TCGA-AR-A255 | Positive | [Not Available] | Positive | [Not Available] | Positive | [Not Available] | 3+ | [Not Evaluated] | Luminal B like (HER2 pos) |
| TCGA-AR-A256 | Negative | [Not Available] | Negative | [Not Available] | Negative | [Not Available] | 1+ | [Not Evaluated] | Triple negative |
| TCGA-AR-A2LE | Positive | [Not Available] | Negative | [Not Available] | [Not Evaluated] | [Not Available] | [Not Available] | [Not Evaluated] | Undetermined |
| TCGA-AR-A2LH | Negative | [Not Available] | Negative | [Not Available] | Equivocal | [Not Available] | 2+ | Negative | Triple negative |
| TCGA-AR-A2LJ | Positive | 70-79% | Positive | 70-79% | Positive | [Not Available] | 3+ | Positive | Luminal B like (HER2 pos) |
| TCGA-AR-A2LK | Positive | 70-79% | Positive | [Not Available] | Equivocal | [Not Available] | 1+ | Negative | Luminal-HER2 neg |
| TCGA-AR-A2LL | Positive | 70-79% | Positive | 70-79% | Equivocal | [Not Available] | 2+ | Negative | Luminal-HER2 neg |
| TCGA-AR-A2LM | Positive | 70-79% | Positive | 70-79% | [Not Evaluated] | [Not Available] | [Not Available] | Negative | Luminal-HER2 neg |
| TCGA-AR-A2LN | Positive | 70-79% | Positive | 70-79% | Negative | [Not Available] | 1+ | [Not Evaluated] | Luminal-HER2 neg |
| TCGA-AR-A2LO | Positive | 70-79% | Positive | [Not Available] | Equivocal | [Not Available] | 2+ | Negative | Luminal-HER2 neg |
| TCGA-AR-A2LQ | Positive | 70-79% | Positive | <10% | Negative | [Not Available] | 1+ | [Not Evaluated] | Luminal-HER2 neg |
| TCGA-AR-A2LR | Negative | [Not Available] | Negative | [Not Available] | Negative | [Not Available] | [Not Available] | [Not Evaluated] | Triple negative |
| TCGA-AR-A5QM | Positive | [Not Available] | Positive | [Not Available] | Equivocal | [Not Available] | 2+ | [Not Available] | Undetermined |
| TCGA-AR-A5QN | Positive | [Not Available] | Positive | [Not Available] | Positive | [Not Available] | 3+ | [Not Available] | Luminal B like (HER2 pos) |
| TCGA-AR-A5QP | Positive | [Not Available] | Positive | [Not Available] | Equivocal | [Not Available] | 2+ | [Not Available] | Undetermined |
| TCGA-AR-A5QQ | Negative | [Not Available] | Negative | [Not Available] | Negative | [Not Available] | 1+ | [Not Available] | Triple negative |
| TCGA-B6-A0I1 | Negative | [Not Available] | Negative | [Not Available] | [Not Evaluated] | [Not Available] | [Not Available] | [Not Evaluated] | Undetermined |
| TCGA-B6-A0I2 | [Not Evaluated] | [Not Available] | [Not Evaluated] | [Not Available] | [Not Evaluated] | [Not Available] | [Not Available] | [Not Evaluated] | Undetermined |
| TCGA-B6-A0I5 | Positive | [Not Available] | Positive | [Not Available] | [Not Evaluated] | [Not Available] | [Not Available] | [Not Available] | Undetermined |
| TCGA-B6-A0I6 | Negative | [Not Available] | Negative | [Not Available] | [Not Evaluated] | [Not Available] | [Not Available] | [Not Available] | Undetermined |
| TCGA-B6-A0I8 | [Not Evaluated] | [Not Available] | [Not Evaluated] | [Not Available] | [Not Evaluated] | [Not Available] | [Not Available] | [Not Available] | Undetermined |
| TCGA-B6-A0I9 | Indeterminate | [Not Available] | Positive | [Not Available] | [Not Evaluated] | [Not Available] | [Not Available] | [Not Available] | Undetermined |
| TCGA-B6-A0IA | Positive | [Not Available] | Positive | [Not Available] | [Not Evaluated] | [Not Available] | [Not Available] | [Not Available] | Undetermined |
| TCGA-B6-A0IB | Positive | [Not Available] | Positive | [Not Available] | [Not Evaluated] | [Not Available] | [Not Available] | [Not Available] | Undetermined |
| TCGA-B6-A0IC | Positive | [Not Available] | Positive | [Not Available] | [Not Evaluated] | [Not Available] | [Not Available] | [Not Available] | Undetermined |
| TCGA-B6-A0IE | Negative | [Not Available] | Negative | [Not Available] | [Not Evaluated] | [Not Available] | [Not Available] | [Not Available] | Undetermined |
| TCGA-B6-A0IG | Positive | [Not Available] | Positive | [Not Available] | [Not Evaluated] | [Not Available] | [Not Available] | [Not Available] | Undetermined |
| TCGA-B6-A0IH | Positive | [Not Available] | Positive | [Not Available] | [Not Evaluated] | [Not Available] | [Not Available] | [Not Evaluated] | Undetermined |
| TCGA-B6-A0IJ | Positive | [Not Available] | Positive | [Not Available] | [Not Evaluated] | [Not Available] | [Not Available] | [Not Available] | Undetermined |
| TCGA-B6-A0IK | Negative | [Not Available] | Negative | [Not Available] | [Not Evaluated] | [Not Available] | [Not Available] | [Not Available] | Undetermined |
| TCGA-B6-A0IM | Positive | [Not Available] | Positive | [Not Available] | [Not Evaluated] | [Not Available] | [Not Available] | [Not Available] | Undetermined |
| TCGA-B6-A0IN | Positive | [Not Available] | Negative | [Not Available] | [Not Evaluated] | [Not Available] | [Not Available] | [Not Available] | Undetermined |
| TCGA-B6-A0IO | Positive | [Not Available] | Indeterminate | [Not Available] | [Not Evaluated] | [Not Available] | [Not Available] | [Not Available] | Undetermined |
| TCGA-B6-A0IP | Positive | [Not Available] | Positive | [Not Available] | [Not Evaluated] | [Not Available] | [Not Available] | [Not Evaluated] | Undetermined |
| TCGA-B6-A0IQ | Negative | [Not Available] | Negative | [Not Available] | [Not Evaluated] | [Not Available] | [Not Available] | [Not Available] | Undetermined |
| TCGA-B6-A0RE | Negative | [Not Available] | Negative | [Not Available] | [Not Evaluated] | [Not Available] | [Not Available] | [Not Available] | Undetermined |
| TCGA-B6-A0RG | Negative | [Not Available] | Negative | [Not Available] | [Not Evaluated] | [Not Available] | [Not Available] | [Not Available] | Undetermined |
| TCGA-B6-A0RH | Positive | [Not Available] | Positive | [Not Available] | [Not Evaluated] | [Not Available] | [Not Available] | [Not Evaluated] | Undetermined |
| TCGA-B6-A0RI | Positive | [Not Available] | Positive | [Not Available] | [Not Evaluated] | [Not Available] | [Not Available] | [Not Available] | Undetermined |
| TCGA-B6-A0RL | Positive | [Not Available] | Positive | [Not Available] | [Not Evaluated] | [Not Available] | [Not Available] | [Not Available] | Undetermined |
| TCGA-B6-A0RM | Positive | [Not Available] | Positive | [Not Available] | [Not Evaluated] | [Not Available] | [Not Available] | [Not Available] | Undetermined |
| TCGA-B6-A0RN | Negative | [Not Available] | Negative | [Not Available] | [Not Evaluated] | [Not Available] | [Not Available] | [Not Available] | Undetermined |
| TCGA-B6-A0RO | Positive | [Not Available] | Positive | [Not Available] | [Not Evaluated] | [Not Available] | [Not Available] | [Not Available] | Undetermined |
| TCGA-B6-A0RP | Positive | [Not Available] | Positive | [Not Available] | [Not Evaluated] | [Not Available] | [Not Available] | [Not Available] | Undetermined |
| TCGA-B6-A0RQ | Positive | [Not Available] | Positive | [Not Available] | [Not Evaluated] | [Not Available] | [Not Available] | [Not Evaluated] | Undetermined |
| TCGA-B6-A0RS | Negative | [Not Available] | Negative | [Not Available] | [Not Evaluated] | [Not Available] | [Not Available] | [Not Available] | Undetermined |
| TCGA-B6-A0RT | Negative | [Not Available] | Negative | [Not Available] | [Not Evaluated] | [Not Available] | [Not Available] | [Not Available] | Undetermined |
| TCGA-B6-A0RU | Negative | [Not Available] | Negative | [Not Available] | [Not Evaluated] | [Not Available] | [Not Available] | [Not Available] | Undetermined |
| TCGA-B6-A0RV | Positive | [Not Available] | Positive | [Not Available] | [Not Evaluated] | [Not Available] | [Not Available] | [Not Available] | Undetermined |
| TCGA-B6-A0WS | Positive | [Not Available] | Positive | [Not Available] | [Not Evaluated] | [Not Available] | [Not Available] | [Not Evaluated] | Undetermined |
| TCGA-B6-A0WT | Positive | [Not Available] | Positive | [Not Available] | [Not Evaluated] | [Not Available] | [Not Available] | [Not Evaluated] | Undetermined |
| TCGA-B6-A0WV | Positive | [Not Available] | Positive | [Not Available] | [Not Evaluated] | [Not Available] | [Not Available] | [Not Evaluated] | Undetermined |
| TCGA-B6-A0WW | Positive | [Not Available] | Positive | [Not Available] | [Not Evaluated] | [Not Available] | [Not Available] | [Not Evaluated] | Undetermined |
| TCGA-B6-A0WX | Negative | [Not Available] | Negative | [Not Available] | [Not Evaluated] | [Not Available] | [Not Available] | [Not Evaluated] | Undetermined |
| TCGA-B6-A0WY | Positive | [Not Available] | Negative | [Not Available] | [Not Evaluated] | [Not Available] | [Not Available] | [Not Evaluated] | Undetermined |
| TCGA-B6-A0WZ | Positive | [Not Available] | Positive | [Not Available] | [Not Evaluated] | [Not Available] | [Not Available] | [Not Evaluated] | Undetermined |
| TCGA-B6-A0X0 | Positive | [Not Available] | Positive | [Not Available] | [Not Evaluated] | [Not Available] | [Not Available] | [Not Evaluated] | Undetermined |
| TCGA-B6-A0X1 | Negative | [Not Available] | Negative | [Not Available] | [Not Evaluated] | [Not Available] | [Not Available] | [Not Evaluated] | Undetermined |
| TCGA-B6-A0X4 | Positive | [Not Available] | Positive | [Not Available] | [Not Evaluated] | [Not Available] | [Not Available] | [Not Evaluated] | Undetermined |
| TCGA-B6-A0X5 | Positive | [Not Available] | Positive | [Not Available] | [Not Evaluated] | [Not Available] | [Not Available] | [Not Evaluated] | Undetermined |
| TCGA-B6-A0X7 | Positive | [Not Available] | Positive | [Not Available] | [Not Evaluated] | [Not Available] | [Not Available] | [Not Evaluated] | Undetermined |
| TCGA-B6-A1KC | Positive | [Not Available] | Negative | [Not Available] | Negative | [Not Available] | [Not Available] | [Not Evaluated] | Luminal-HER2 neg |
| TCGA-B6-A1KF | Negative | [Not Available] | Negative | [Not Available] | Equivocal | 60-69% | 2+ | [Not Evaluated] | Undetermined |
| TCGA-B6-A1KI | Positive | [Not Available] | Positive | [Not Available] | Equivocal | 90-99% | 2+ | Negative | Luminal-HER2 neg |
| TCGA-B6-A1KN | Negative | [Not Available] | Negative | [Not Available] | [Not Evaluated] | [Not Available] | [Not Available] | [Not Evaluated] | Undetermined |
| TCGA-B6-A2IU | Positive | [Not Available] | Positive | [Not Available] | [Not Evaluated] | [Not Available] | [Not Available] | [Not Evaluated] | Undetermined |
| TCGA-B6-A401 | Positive | 80-89% | Positive | 90-99% | Negative | <10% | 1+ | [Not Evaluated] | Luminal-HER2 neg |
| TCGA-B6-A402 | Negative | <10% | Negative | <10% | Negative | <10% | 1+ | [Not Evaluated] | Triple negative |
| TCGA-B6-A408 | Positive | 90-99% | Positive | 60-69% | Equivocal | 20-29% | 2+ | Negative | Luminal-HER2 neg |
| TCGA-B6-A40B | Positive | 90-99% | Positive | 90-99% | Negative | <10% | 1+ | [Not Evaluated] | Luminal-HER2 neg |
| TCGA-B6-A40C | Positive | 50-59% | Positive | 90-99% | Negative | <10% | 1+ | [Not Evaluated] | Luminal-HER2 neg |
| TCGA-BH-A0AU | Positive | [Not Available] | Positive | [Not Available] | Positive | [Not Available] | [Not Available] | Negative | Luminal-HER2 neg |
| TCGA-BH-A0AV | Negative | [Not Available] | Negative | [Not Available] | [Not Available] | [Not Available] | [Not Available] | Negative | Triple negative |
| TCGA-BH-A0AW | Positive | [Not Available] | Negative | [Not Available] | Positive | [Not Available] | 3+ | [Not Evaluated] | Luminal B like (HER2 pos) |
| TCGA-BH-A0AY | Positive | [Not Available] | Positive | [Not Available] | Negative | [Not Available] | 0 | [Not Evaluated] | Luminal-HER2 neg |
| TCGA-BH-A0AZ | Positive | [Not Available] | Positive | [Not Available] | Negative | [Not Available] | [Not Available] | [Not Available] | Luminal-HER2 neg |
| TCGA-BH-A0B0 | Positive | [Not Available] | Positive | [Not Available] | Negative | [Not Available] | [Not Available] | [Not Available] | Luminal-HER2 neg |
| TCGA-BH-A0B1 | Positive | 90-99% | Positive | 40-49% | Negative | [Not Available] | 1+ | [Not Evaluated] | Luminal-HER2 neg |
| TCGA-BH-A0B2 | Positive | [Not Available] | Positive | [Not Available] | Negative | [Not Available] | [Not Available] | [Not Available] | Luminal-HER2 neg |
| TCGA-BH-A0B3 | Negative | [Not Available] | Negative | [Not Available] | Negative | [Not Available] | 1+ | [Not Evaluated] | Triple negative |
| TCGA-BH-A0B4 | Positive | 80-89% | Positive | 50-59% | Equivocal | [Not Available] | 2+ | Positive | Luminal B like (HER2 pos) |
| TCGA-BH-A0B5 | Positive | [Not Available] | Positive | [Not Available] | Negative | [Not Available] | [Not Available] | [Not Available] | Luminal-HER2 neg |
| TCGA-BH-A0B6 | Positive | [Not Available] | Positive | [Not Available] | Positive | [Not Available] | [Not Available] | [Not Available] | Luminal B like (HER2 pos) |
| TCGA-BH-A0B7 | Positive | [Not Available] | Positive | [Not Available] | Positive | [Not Available] | 3+ | [Not Available] | Luminal B like (HER2 pos) |
| TCGA-BH-A0B8 | Positive | 90-99% | Positive | 30-39% | Equivocal | [Not Available] | 2+ | Negative | Luminal-HER2 neg |
| TCGA-BH-A0B9 | Negative | [Not Available] | Negative | [Not Available] | Negative | <10% | 0 | [Not Evaluated] | Triple negative |
| TCGA-BH-A0BA | Positive | 80-89% | Positive | 40-49% | Negative | [Not Available] | 1+ | [Not Evaluated] | Luminal-HER2 neg |
| TCGA-BH-A0BC | Positive | 90-99% | Positive | 30-39% | Negative | <10% | 0 | [Not Evaluated] | Luminal-HER2 neg |
| TCGA-BH-A0BD | Positive | [Not Available] | Positive | [Not Available] | Negative | [Not Available] | 1+ | [Not Evaluated] | Luminal-HER2 neg |
| TCGA-BH-A0BF | Positive | [Not Available] | Positive | [Not Available] | Negative | [Not Available] | [Not Available] | [Not Available] | Luminal-HER2 neg |
| TCGA-BH-A0BG | Negative | [Not Available] | Negative | [Not Available] | Negative | [Not Available] | [Not Available] | [Not Available] | Triple negative |
| TCGA-BH-A0BJ | Positive | 90-99% | Positive | 70-79% | Negative | [Not Available] | 1+ | [Not Evaluated] | Luminal-HER2 neg |
| TCGA-BH-A0BL | Negative | [Not Available] | Negative | [Not Available] | Negative | [Not Available] | [Not Available] | [Not Available] | Triple negative |
| TCGA-BH-A0BM | Positive | [Not Available] | Negative | [Not Available] | Negative | <10% | 0 | [Not Evaluated] | Luminal-HER2 neg |
| TCGA-BH-A0BO | Positive | [Not Available] | Positive | [Not Available] | Negative | [Not Available] | [Not Available] | [Not Available] | Luminal-HER2 neg |
| TCGA-BH-A0BP | Positive | [Not Available] | Positive | [Not Available] | Negative | [Not Available] | [Not Available] | [Not Available] | Luminal-HER2 neg |
| TCGA-BH-A0BQ | Positive | [Not Available] | Positive | [Not Available] | Positive | [Not Available] | [Not Available] | [Not Available] | Luminal B like (HER2 pos) |
| TCGA-BH-A0BR | Positive | [Not Available] | Positive | [Not Available] | Negative | [Not Available] | [Not Available] | [Not Available] | Luminal-HER2 neg |
| TCGA-BH-A0BS | Positive | [Not Available] | Positive | [Not Available] | [Not Available] | [Not Available] | [Not Available] | Negative | Luminal-HER2 neg |
| TCGA-BH-A0BT | Positive | [Not Available] | Positive | [Not Available] | Negative | [Not Available] | [Not Available] | [Not Available] | Luminal-HER2 neg |
| TCGA-BH-A0BV | Positive | 90-99% | Positive | 50-59% | Negative | <10% | 0 | [Not Evaluated] | Luminal-HER2 neg |
| TCGA-BH-A0BW | Negative | [Not Available] | Negative | [Not Available] | [Not Available] | [Not Available] | [Not Available] | Negative | Triple negative |
| TCGA-BH-A0BZ | Positive | [Not Available] | Positive | [Not Available] | Negative | [Not Available] | [Not Available] | [Not Available] | Luminal-HER2 neg |
| TCGA-BH-A0C0 | Positive | 90-99% | Positive | 60-69% | Equivocal | [Not Available] | 2+ | Positive | Luminal B like (HER2 pos) |
| TCGA-BH-A0C1 | Positive | [Not Available] | Positive | [Not Available] | Negative | [Not Available] | [Not Available] | [Not Available] | Luminal-HER2 neg |
| TCGA-BH-A0C3 | Positive | [Not Available] | Negative | [Not Available] | Negative | [Not Available] | [Not Available] | [Not Available] | Luminal-HER2 neg |
| TCGA-BH-A0C7 | Positive | [Not Available] | Negative | [Not Available] | [Not Available] | [Not Available] | [Not Available] | Positive | Luminal B like (HER2 pos) |
| TCGA-BH-A0DD | Positive | [Not Available] | Positive | [Not Available] | Positive | [Not Available] | [Not Available] | Positive | Luminal B like (HER2 pos) |
| TCGA-BH-A0DE | Positive | [Not Available] | Positive | [Not Available] | Negative | [Not Available] | [Not Available] | [Not Available] | Luminal-HER2 neg |
| TCGA-BH-A0DG | Positive | [Not Available] | Negative | [Not Available] | Negative | [Not Available] | [Not Available] | [Not Available] | Luminal-HER2 neg |
| TCGA-BH-A0DH | Positive | 90-99% | Positive | 40-49% | Negative | <10% | 0 | [Not Evaluated] | Luminal-HER2 neg |
| TCGA-BH-A0DI | Positive | [Not Available] | Positive | [Not Available] | Negative | [Not Available] | [Not Available] | [Not Available] | Luminal-HER2 neg |
| TCGA-BH-A0DK | Positive | [Not Available] | Positive | [Not Available] | Negative | [Not Available] | 0 | [Not Evaluated] | Luminal-HER2 neg |
| TCGA-BH-A0DL | Positive | [Not Available] | Negative | [Not Available] | Negative | [Not Available] | [Not Available] | [Not Available] | Luminal-HER2 neg |
| TCGA-BH-A0DO | Positive | [Not Available] | Positive | [Not Available] | Negative | [Not Available] | [Not Available] | [Not Available] | Luminal-HER2 neg |
| TCGA-BH-A0DP | Positive | 80-89% | Positive | 80-89% | Negative | <10% | 0 | [Not Evaluated] | Luminal-HER2 neg |
| TCGA-BH-A0DQ | Positive | 60-69% | Positive | 70-79% | Negative | <10% | 0 | [Not Evaluated] | Luminal-HER2 neg |
| TCGA-BH-A0DS | Positive | 70-79% | Positive | 70-79% | Negative | [Not Available] | 1+ | [Not Evaluated] | Luminal-HER2 neg |
| TCGA-BH-A0DT | Positive | [Not Available] | Positive | [Not Available] | Negative | [Not Available] | [Not Available] | [Not Available] | Luminal-HER2 neg |
| TCGA-BH-A0DV | Positive | [Not Available] | Positive | [Not Available] | Negative | [Not Available] | [Not Available] | [Not Available] | Luminal-HER2 neg |
| TCGA-BH-A0DX | Positive | [Not Available] | Positive | [Not Available] | Negative | [Not Available] | [Not Available] | [Not Available] | Luminal-HER2 neg |
| TCGA-BH-A0DZ | Positive | 50-59% | Positive | <10% | Positive | 30-39% | 3+ | [Not Evaluated] | Luminal B like (HER2 pos) |
| TCGA-BH-A0E0 | Negative | [Not Available] | Negative | [Not Available] | Negative | [Not Available] | 1+ | [Not Evaluated] | Triple negative |
| TCGA-BH-A0E1 | Positive | 70-79% | Positive | 50-59% | Equivocal | [Not Available] | 2+ | Negative | Luminal-HER2 neg |
| TCGA-BH-A0E2 | Positive | 90-99% | Positive | <10% | Negative | [Not Available] | 1+ | [Not Evaluated] | Luminal-HER2 neg |
| TCGA-BH-A0E6 | Negative | [Not Available] | Negative | [Not Available] | Equivocal | [Not Available] | 2+ | Negative | Triple negative |
| TCGA-BH-A0E7 | Positive | 50-59% | Positive | 60-69% | Negative | [Not Available] | 1+ | [Not Evaluated] | Luminal-HER2 neg |
| TCGA-BH-A0E9 | Positive | [Not Available] | Positive | [Not Available] | Negative | [Not Available] | [Not Available] | [Not Available] | Luminal-HER2 neg |
| TCGA-BH-A0EA | Positive | [Not Available] | Positive | [Not Available] | Negative | [Not Available] | [Not Available] | [Not Available] | Luminal-HER2 neg |
| TCGA-BH-A0EB | Positive | 90-99% | Positive | 40-49% | Equivocal | [Not Available] | 2+ | Negative | Luminal-HER2 neg |
| TCGA-BH-A0EE | Negative | [Not Available] | Negative | [Not Available] | Positive | 30-39% | 3+ | [Not Evaluated] | HER2-positive (non-luminal) |
| TCGA-BH-A0EI | Positive | [Not Available] | Positive | [Not Available] | Negative | [Not Available] | [Not Available] | [Not Available] | Luminal-HER2 neg |
| TCGA-BH-A0GY | Positive | 40-49% | Positive | 90-99% | Negative | [Not Available] | 1+ | [Not Evaluated] | Luminal-HER2 neg |
| TCGA-BH-A0GZ | Positive | 90-99% | Positive | 80-89% | Equivocal | [Not Available] | 2+ | Negative | Luminal-HER2 neg |
| TCGA-BH-A0H0 | Positive | 50-59% | Positive | <10% | Negative | <10% | 0 | [Not Evaluated] | Luminal-HER2 neg |
| TCGA-BH-A0H3 | Positive | [Not Available] | Positive | [Not Available] | [Not Available] | [Not Available] | [Not Available] | Negative | Luminal-HER2 neg |
| TCGA-BH-A0H5 | Positive | [Not Available] | Positive | [Not Available] | [Not Available] | [Not Available] | [Not Available] | Negative | Luminal-HER2 neg |
| TCGA-BH-A0H6 | Positive | 50-59% | Positive | 60-69% | Negative | [Not Available] | 1+ | [Not Evaluated] | Luminal-HER2 neg |
| TCGA-BH-A0H7 | Positive | 70-79% | Positive | 30-39% | Negative | [Not Available] | 1+ | [Not Evaluated] | Luminal-HER2 neg |
| TCGA-BH-A0H9 | Positive | 90-99% | Positive | 30-39% | Equivocal | [Not Available] | 2+ | Negative | Luminal-HER2 neg |
| TCGA-BH-A0HA | Positive | [Not Available] | Positive | [Not Available] | [Not Available] | [Not Available] | [Not Available] | Negative | Luminal-HER2 neg |
| TCGA-BH-A0HB | Positive | 90-99% | Positive | 30-39% | Equivocal | [Not Available] | 2+ | Negative | Luminal-HER2 neg |
| TCGA-BH-A0HF | Positive | 90-99% | Positive | <10% | Negative | <10% | 0 | [Not Evaluated] | Luminal-HER2 neg |
| TCGA-BH-A0HI | Positive | 50-59% | Positive | 40-49% | Negative | [Not Available] | 1+ | [Not Evaluated] | Luminal-HER2 neg |
| TCGA-BH-A0HK | Positive | 70-79% | Negative | <10% | Negative | <10% | 0 | [Not Evaluated] | Luminal-HER2 neg |
| TCGA-BH-A0HL | Positive | 90-99% | Positive | 40-49% | Equivocal | [Not Available] | 2+ | Negative | Luminal-HER2 neg |
| TCGA-BH-A0HN | Positive | 90-99% | Positive | 90-99% | Equivocal | [Not Available] | 2+ | Negative | Luminal-HER2 neg |
| TCGA-BH-A0HO | Positive | 90-99% | Positive | 90-99% | Negative | [Not Available] | 1+ | [Not Evaluated] | Luminal-HER2 neg |
| TCGA-BH-A0HP | Positive | 80-89% | Negative | [Not Available] | Negative | [Not Available] | 1+ | [Not Evaluated] | Luminal-HER2 neg |
| TCGA-BH-A0HQ | Positive | 90-99% | Positive | 90-99% | Negative | [Not Available] | 1+ | [Not Evaluated] | Luminal-HER2 neg |
| TCGA-BH-A0HU | Positive | 40-49% | Positive | 40-49% | Negative | [Not Available] | 1+ | [Not Evaluated] | Luminal-HER2 neg |
| TCGA-BH-A0HW | Positive | 90-99% | Negative | <10% | Equivocal | [Not Available] | 2+ | Negative | Luminal-HER2 neg |
| TCGA-BH-A0HX | Positive | 80-89% | Positive | 50-59% | Negative | <10% | [Not Available] | [Not Evaluated] | Luminal-HER2 neg |
| TCGA-BH-A0HY | Positive | [Not Available] | Negative | [Not Available] | Positive | [Not Available] | [Not Available] | [Not Available] | Luminal B like (HER2 pos) |
| TCGA-BH-A0RX | Negative | <10% | Negative | <10% | Negative | [Not Available] | 1+ | [Not Evaluated] | Triple negative |
| TCGA-BH-A0W3 | Positive | [Not Available] | Positive | [Not Available] | Equivocal | [Not Available] | [Not Available] | Negative | Luminal-HER2 neg |
| TCGA-BH-A0W4 | Positive | [Not Available] | Positive | [Not Available] | Negative | [Not Available] | [Not Available] | [Not Evaluated] | Luminal-HER2 neg |
| TCGA-BH-A0W5 | Positive | [Not Available] | Positive | [Not Available] | Equivocal | [Not Available] | [Not Available] | Equivocal | Undetermined |
| TCGA-BH-A0W7 | Positive | [Not Available] | Positive | [Not Available] | Negative | [Not Available] | [Not Available] | [Not Evaluated] | Luminal-HER2 neg |
| TCGA-BH-A0WA | Negative | [Not Available] | Negative | [Not Available] | Negative | [Not Available] | [Not Available] | [Not Evaluated] | Triple negative |
| TCGA-BH-A18F | Positive | 70-79% | Positive | 40-49% | Negative | [Not Available] | [Not Available] | [Not Available] | Luminal-HER2 neg |
| TCGA-BH-A18G | Negative | [Not Available] | Negative | [Not Available] | Negative | [Not Available] | [Not Available] | [Not Available] | Triple negative |
| TCGA-BH-A18H | Positive | [Not Available] | Positive | [Not Available] | Positive | [Not Available] | [Not Available] | Equivocal | Luminal B like (HER2 pos) |
| TCGA-BH-A18I | Positive | [Not Available] | Positive | [Not Available] | Positive | [Not Available] | [Not Available] | [Not Available] | Luminal B like (HER2 pos) |
| TCGA-BH-A18J | Positive | 40-49% | Positive | 30-39% | Negative | [Not Available] | [Not Available] | [Not Available] | Luminal-HER2 neg |
| TCGA-BH-A18K | Positive | [Not Available] | Positive | [Not Available] | Negative | [Not Available] | [Not Available] | [Not Available] | Luminal-HER2 neg |
| TCGA-BH-A18L | Positive | [Not Available] | Positive | [Not Available] | Negative | [Not Available] | [Not Available] | [Not Available] | Luminal-HER2 neg |
| TCGA-BH-A18M | Positive | [Not Available] | Positive | [Not Available] | Positive | [Not Available] | [Not Available] | Positive | Luminal B like (HER2 pos) |
| TCGA-BH-A18N | Positive | [Not Available] | Positive | [Not Available] | Negative | [Not Available] | [Not Available] | [Not Available] | Luminal-HER2 neg |
| TCGA-BH-A18P | Positive | <10% | Negative | [Not Available] | Positive | 10-19% | 2+ | Positive | Luminal B like (HER2 pos) |
| TCGA-BH-A18Q | Negative | [Not Available] | Negative | [Not Available] | Positive | [Not Available] | [Not Available] | Negative | Triple negative |
| TCGA-BH-A18R | Indeterminate | 20-29% | Negative | [Not Available] | Positive | [Not Available] | 3+ | Positive | Undetermined |
| TCGA-BH-A18S | Positive | [Not Available] | Positive | [Not Available] | Negative | [Not Available] | [Not Available] | [Not Available] | Luminal-HER2 neg |
| TCGA-BH-A18T | Negative | [Not Available] | Negative | [Not Available] | Positive | [Not Available] | 2+ | Negative | Triple negative |
| TCGA-BH-A18U | Positive | [Not Available] | Positive | [Not Available] | Positive | [Not Available] | 3+ | [Not Available] | Luminal B like (HER2 pos) |
| TCGA-BH-A18V | Negative | [Not Available] | Negative | [Not Available] | Negative | [Not Available] | [Not Available] | [Not Available] | Triple negative |
| TCGA-BH-A1EN | Negative | [Not Available] | Negative | [Not Available] | Positive | [Not Available] | [Not Available] | [Not Available] | HER2-positive (non-luminal) |
| TCGA-BH-A1EO | Positive | [Not Available] | Positive | [Not Available] | Negative | [Not Available] | [Not Available] | [Not Available] | Luminal-HER2 neg |
| TCGA-BH-A1ES | Positive | [Not Available] | Positive | [Not Available] | Negative | [Not Available] | [Not Available] | [Not Available] | Luminal-HER2 neg |
| TCGA-BH-A1ET | Positive | [Not Available] | Positive | [Not Available] | Negative | [Not Available] | [Not Available] | [Not Evaluated] | Luminal-HER2 neg |
| TCGA-BH-A1EU | Positive | [Not Available] | Positive | [Not Available] | Negative | [Not Available] | [Not Available] | [Not Evaluated] | Luminal-HER2 neg |
| TCGA-BH-A1EV | Positive | [Not Available] | Positive | [Not Available] | Positive | [Not Available] | [Not Available] | [Not Evaluated] | Luminal B like (HER2 pos) |
| TCGA-BH-A1EW | Negative | [Not Available] | Negative | [Not Available] | Negative | [Not Available] | [Not Available] | [Not Evaluated] | Triple negative |
| TCGA-BH-A1EX | Positive | [Not Available] | Positive | [Not Available] | Positive | [Not Available] | [Not Available] | Negative | Luminal-HER2 neg |
| TCGA-BH-A1EY | Positive | [Not Available] | Positive | [Not Available] | Negative | [Not Available] | [Not Available] | [Not Available] | Luminal-HER2 neg |
| TCGA-BH-A1F0 | Negative | [Not Available] | Indeterminate | [Not Available] | Negative | [Not Available] | [Not Available] | [Not Evaluated] | Undetermined |
| TCGA-BH-A1F2 | Positive | [Not Available] | Positive | [Not Available] | Positive | [Not Available] | [Not Available] | [Not Available] | Luminal B like (HER2 pos) |
| TCGA-BH-A1F5 | Positive | [Not Available] | Positive | [Not Available] | Negative | [Not Available] | [Not Available] | [Not Evaluated] | Luminal-HER2 neg |
| TCGA-BH-A1F6 | Negative | [Not Available] | Negative | [Not Available] | Negative | [Not Available] | [Not Available] | [Not Evaluated] | Triple negative |
| TCGA-BH-A1F8 | Positive | [Not Available] | Positive | [Not Available] | Positive | [Not Available] | [Not Available] | [Not Available] | Luminal B like (HER2 pos) |
| TCGA-BH-A1FB | Positive | [Not Available] | Positive | [Not Available] | Negative | [Not Available] | [Not Available] | [Not Available] | Luminal-HER2 neg |
| TCGA-BH-A1FC | Negative | [Not Available] | Negative | [Not Available] | Negative | [Not Available] | [Not Available] | [Not Available] | Triple negative |
| TCGA-BH-A1FD | Positive | [Not Available] | Positive | [Not Available] | Negative | [Not Available] | [Not Available] | [Not Evaluated] | Luminal-HER2 neg |
| TCGA-BH-A1FE | Positive | [Not Available] | Positive | [Not Available] | [Not Evaluated] | [Not Available] | [Not Available] | [Not Evaluated] | Undetermined |
| TCGA-BH-A1FG | Positive | [Not Available] | Positive | [Not Available] | Negative | [Not Available] | [Not Available] | [Not Available] | Luminal-HER2 neg |
| TCGA-BH-A1FH | Positive | [Not Available] | Negative | [Not Available] | [Not Evaluated] | [Not Available] | [Not Available] | [Not Evaluated] | Undetermined |
| TCGA-BH-A1FJ | Negative | [Not Available] | Positive | [Not Available] | [Not Evaluated] | [Not Available] | [Not Available] | [Not Evaluated] | Undetermined |
| TCGA-BH-A1FL | Positive | [Not Available] | Positive | [Not Available] | [Not Evaluated] | [Not Available] | [Not Available] | [Not Evaluated] | Undetermined |
| TCGA-BH-A1FM | Positive | [Not Available] | Negative | [Not Available] | [Not Evaluated] | [Not Available] | [Not Available] | [Not Evaluated] | Undetermined |
| TCGA-BH-A1FN | Positive | [Not Available] | Positive | [Not Available] | [Not Evaluated] | [Not Available] | [Not Available] | [Not Evaluated] | Undetermined |
| TCGA-BH-A1FR | Positive | [Not Available] | Positive | [Not Available] | [Not Evaluated] | [Not Available] | [Not Available] | [Not Evaluated] | Undetermined |
| TCGA-BH-A1FU | Negative | [Not Available] | Negative | [Not Available] | [Not Evaluated] | [Not Available] | [Not Available] | [Not Evaluated] | Undetermined |
| TCGA-BH-A201 | Positive | [Not Available] | Positive | [Not Available] | Negative | [Not Available] | [Not Available] | [Not Evaluated] | Luminal-HER2 neg |
| TCGA-BH-A202 | Positive | [Not Available] | Positive | [Not Available] | Positive | [Not Available] | [Not Available] | [Not Evaluated] | Luminal B like (HER2 pos) |
| TCGA-BH-A203 | [Not Evaluated] | [Not Available] | [Not Evaluated] | [Not Available] | [Not Evaluated] | [Not Available] | [Not Available] | [Not Evaluated] | Undetermined |
| TCGA-BH-A204 | [Not Evaluated] | [Not Available] | [Not Evaluated] | [Not Available] | [Not Evaluated] | [Not Available] | [Not Available] | [Not Evaluated] | Undetermined |
| TCGA-BH-A208 | [Not Evaluated] | [Not Available] | [Not Evaluated] | [Not Available] | [Not Evaluated] | [Not Available] | [Not Available] | [Not Evaluated] | Undetermined |
| TCGA-BH-A209 | Positive | [Not Available] | Positive | [Not Available] | [Not Evaluated] | [Not Available] | [Not Available] | [Not Evaluated] | Undetermined |
| TCGA-BH-A28O | Positive | [Not Available] | Positive | [Not Available] | Negative | [Not Available] | [Not Available] | [Not Evaluated] | Luminal-HER2 neg |
| TCGA-BH-A28Q | Positive | [Not Available] | Positive | [Not Available] | Negative | [Not Available] | [Not Available] | Indeterminate | Luminal-HER2 neg |
| TCGA-BH-A2L8 | Positive | 70-79% | Positive | 80-89% | Negative | [Not Available] | [Not Available] | Negative | Luminal-HER2 neg |
| TCGA-BH-A42T | Positive | [Not Available] | Positive | [Not Available] | Positive | [Not Available] | 2+ | Equivocal | Luminal B like (HER2 pos) |
| TCGA-BH-A42U | Negative | [Not Available] | Negative | [Not Available] | Negative | [Not Available] | [Not Available] | [Not Evaluated] | Triple negative |
| TCGA-BH-A42V | Positive | [Not Available] | Positive | [Not Available] | Negative | [Not Available] | [Not Available] | [Not Evaluated] | Luminal-HER2 neg |
| TCGA-BH-A5IZ | Positive | [Not Available] | Negative | [Not Available] | Negative | [Not Available] | [Not Available] | Negative | Luminal-HER2 neg |
| TCGA-BH-A5J0 | Positive | [Not Available] | Positive | [Not Available] | Negative | [Not Available] | [Not Available] | [Not Evaluated] | Luminal-HER2 neg |
| TCGA-BH-A6R8 | Positive | [Not Available] | Positive | [Not Available] | [Not Evaluated] | [Not Available] | [Not Available] | Negative | Luminal-HER2 neg |
| TCGA-BH-A6R9 | Negative | [Not Available] | Positive | [Not Available] | Negative | [Not Available] | [Not Available] | [Not Evaluated] | Luminal-HER2 neg |
| TCGA-BH-A8FY | Positive | [Not Available] | Positive | [Not Available] | Negative | [Not Available] | [Not Available] | [Not Evaluated] | Luminal-HER2 neg |
| TCGA-BH-A8FZ | Positive | [Not Available] | Positive | [Not Available] | Equivocal | [Not Available] | [Not Available] | [Not Evaluated] | Undetermined |
| TCGA-BH-A8G0 | Positive | [Not Available] | Positive | [Not Available] | Negative | [Not Available] | [Not Available] | [Not Evaluated] | Luminal-HER2 neg |
| TCGA-BH-AB28 | Positive | [Not Available] | Positive | [Not Available] | Negative | [Not Available] | [Not Available] | Indeterminate | Luminal-HER2 neg |
| TCGA-C8-A12K | [Not Evaluated] | [Not Available] | [Not Evaluated] | [Not Available] | [Not Evaluated] | [Not Available] | [Not Available] | [Not Evaluated] | Undetermined |
| TCGA-C8-A12L | Negative | [Not Available] | Negative | [Not Available] | Equivocal | [Not Available] | 2+ | [Not Evaluated] | Undetermined |
| TCGA-C8-A12M | Positive | [Not Available] | Negative | [Not Available] | Negative | [Not Available] | [Not Available] | [Not Evaluated] | Luminal-HER2 neg |
| TCGA-C8-A12N | Positive | [Not Available] | Positive | [Not Available] | Negative | [Not Available] | [Not Available] | [Not Evaluated] | Luminal-HER2 neg |
| TCGA-C8-A12O | Positive | [Not Available] | Positive | [Not Available] | Negative | [Not Available] | [Not Available] | [Not Evaluated] | Luminal-HER2 neg |
| TCGA-C8-A12P | Negative | [Not Available] | Negative | [Not Available] | Positive | [Not Available] | 3+ | [Not Evaluated] | HER2-positive (non-luminal) |
| TCGA-C8-A12Q | Negative | [Not Available] | Negative | [Not Available] | Positive | [Not Available] | 3+ | [Not Evaluated] | HER2-positive (non-luminal) |
| TCGA-C8-A12T | Positive | [Not Available] | Positive | [Not Available] | Positive | [Not Available] | 3+ | [Not Evaluated] | Luminal B like (HER2 pos) |
| TCGA-C8-A12U | Positive | [Not Available] | Positive | [Not Available] | Negative | [Not Available] | 1+ | [Not Evaluated] | Luminal-HER2 neg |
| TCGA-C8-A12V | Negative | [Not Available] | Negative | [Not Available] | Negative | [Not Available] | [Not Available] | [Not Evaluated] | Triple negative |
| TCGA-C8-A12W | Positive | [Not Available] | Positive | [Not Available] | Negative | [Not Available] | 1+ | [Not Evaluated] | Luminal-HER2 neg |
| TCGA-C8-A12X | Positive | [Not Available] | Positive | [Not Available] | Negative | [Not Available] | 1+ | [Not Evaluated] | Luminal-HER2 neg |
| TCGA-C8-A12Y | [Not Evaluated] | [Not Available] | [Not Evaluated] | [Not Available] | [Not Evaluated] | [Not Available] | [Not Available] | [Not Evaluated] | Undetermined |
| TCGA-C8-A12Z | Negative | [Not Available] | Negative | [Not Available] | Positive | [Not Available] | 3+ | [Not Evaluated] | HER2-positive (non-luminal) |
| TCGA-C8-A130 | Positive | [Not Available] | Positive | [Not Available] | Equivocal | [Not Available] | 2+ | [Not Evaluated] | Undetermined |
| TCGA-C8-A131 | Negative | [Not Available] | Negative | [Not Available] | Negative | [Not Available] | 1+ | [Not Evaluated] | Triple negative |
| TCGA-C8-A132 | Positive | [Not Available] | Positive | [Not Available] | [Not Evaluated] | [Not Available] | [Not Available] | [Not Evaluated] | Undetermined |
| TCGA-C8-A133 | Positive | [Not Available] | Positive | [Not Available] | [Not Evaluated] | [Not Available] | [Not Available] | [Not Evaluated] | Undetermined |
| TCGA-C8-A134 | Negative | [Not Available] | Negative | [Not Available] | Equivocal | [Not Available] | 2+ | [Not Evaluated] | Undetermined |
| TCGA-C8-A135 | Negative | [Not Available] | Negative | [Not Available] | Positive | [Not Available] | 3+ | [Not Evaluated] | HER2-positive (non-luminal) |
| TCGA-C8-A137 | Negative | [Not Available] | Negative | [Not Available] | Positive | [Not Available] | 3+ | [Not Evaluated] | HER2-positive (non-luminal) |
| TCGA-C8-A138 | Positive | [Not Available] | Negative | [Not Available] | Equivocal | [Not Available] | 2+ | [Not Evaluated] | Undetermined |
| TCGA-C8-A1HE | Positive | [Not Available] | Positive | [Not Available] | Equivocal | [Not Available] | 2+ | [Not Evaluated] | Undetermined |
| TCGA-C8-A1HF | Negative | [Not Available] | Positive | [Not Available] | Positive | [Not Available] | 3+ | [Not Evaluated] | Undetermined |
| TCGA-C8-A1HG | Positive | [Not Available] | Positive | [Not Available] | Negative | [Not Available] | 1+ | [Not Evaluated] | Luminal-HER2 neg |
| TCGA-C8-A1HI | Positive | [Not Available] | Positive | [Not Available] | Negative | [Not Available] | 1+ | [Not Evaluated] | Luminal-HER2 neg |
| TCGA-C8-A1HJ | Negative | [Not Available] | Negative | [Not Available] | Negative | [Not Available] | [Not Available] | [Not Evaluated] | Triple negative |
| TCGA-C8-A1HK | Negative | [Not Available] | Negative | [Not Available] | Positive | [Not Available] | 3+ | [Not Evaluated] | HER2-positive (non-luminal) |
| TCGA-C8-A1HL | Positive | [Not Available] | Negative | [Not Available] | Equivocal | [Not Available] | 2+ | [Not Evaluated] | Undetermined |
| TCGA-C8-A1HM | Positive | [Not Available] | Positive | [Not Available] | Negative | [Not Available] | 1+ | [Not Evaluated] | Luminal-HER2 neg |
| TCGA-C8-A1HN | Positive | [Not Available] | Positive | [Not Available] | Equivocal | [Not Available] | 2+ | [Not Evaluated] | Undetermined |
| TCGA-C8-A1HO | Positive | [Not Available] | Positive | [Not Available] | Negative | [Not Available] | [Not Available] | [Not Evaluated] | Luminal-HER2 neg |
| TCGA-C8-A26V | Positive | [Not Available] | Positive | [Not Available] | Negative | [Not Available] | [Not Available] | [Not Evaluated] | Luminal-HER2 neg |
| TCGA-C8-A26W | Positive | [Not Available] | Positive | [Not Available] | Equivocal | [Not Available] | 2+ | [Not Evaluated] | Undetermined |
| TCGA-C8-A26X | Negative | [Not Available] | Negative | [Not Available] | Negative | [Not Available] | 1+ | [Not Evaluated] | Triple negative |
| TCGA-C8-A26Y | Negative | [Not Available] | Negative | [Not Available] | Negative | [Not Available] | 1+ | [Not Evaluated] | Triple negative |
| TCGA-C8-A26Z | Positive | [Not Available] | Positive | [Not Available] | Negative | [Not Available] | 1+ | [Not Evaluated] | Luminal-HER2 neg |
| TCGA-C8-A273 | Positive | [Not Available] | Positive | [Not Available] | Negative | [Not Available] | [Not Available] | [Not Evaluated] | Luminal-HER2 neg |
| TCGA-C8-A274 | Positive | [Not Available] | Positive | [Not Available] | Negative | [Not Available] | 1+ | [Not Evaluated] | Luminal-HER2 neg |
| TCGA-C8-A275 | [Not Evaluated] | [Not Available] | [Not Evaluated] | [Not Available] | [Not Evaluated] | [Not Available] | [Not Available] | [Not Evaluated] | Undetermined |
| TCGA-C8-A278 | Negative | [Not Available] | Negative | [Not Available] | Positive | [Not Available] | 3+ | [Not Evaluated] | HER2-positive (non-luminal) |
| TCGA-C8-A27A | Positive | [Not Available] | Positive | [Not Available] | Negative | [Not Available] | 1+ | [Not Evaluated] | Luminal-HER2 neg |
| TCGA-C8-A27B | Negative | [Not Available] | Negative | [Not Available] | Negative | [Not Available] | [Not Available] | [Not Evaluated] | Triple negative |
| TCGA-C8-A3M7 | Negative | [Not Available] | Negative | [Not Available] | Negative | [Not Available] | [Not Available] | [Not Evaluated] | Triple negative |
| TCGA-C8-A3M8 | Positive | [Not Available] | Positive | [Not Available] | Positive | [Not Available] | 3+ | [Not Evaluated] | Luminal B like (HER2 pos) |
| TCGA-C8-A8HP | Negative | [Not Available] | Negative | [Not Available] | Positive | [Not Available] | [Not Available] | [Not Evaluated] | HER2-positive (non-luminal) |
| TCGA-C8-A8HQ | Positive | [Not Available] | Positive | [Not Available] | Negative | [Not Available] | [Not Available] | [Not Evaluated] | Luminal-HER2 neg |
| TCGA-C8-A8HR | [Not Evaluated] | [Not Available] | [Not Evaluated] | [Not Available] | [Not Evaluated] | [Not Available] | [Not Available] | [Not Evaluated] | Undetermined |
| TCGA-C8-A9FZ | [Not Evaluated] | [Not Available] | [Not Evaluated] | [Not Available] | Negative | [Not Available] | [Not Available] | [Not Evaluated] | Undetermined |
| TCGA-D8-A13Y | Positive | 70-79% | Positive | 70-79% | Negative | [Not Available] | 1+ | [Not Evaluated] | Luminal-HER2 neg |
| TCGA-D8-A13Z | Negative | [Not Available] | Negative | [Not Available] | Negative | [Not Available] | 1+ | [Not Evaluated] | Triple negative |
| TCGA-D8-A140 | Positive | [Not Available] | Positive | <10% | Positive | [Not Available] | 3+ | [Not Evaluated] | Luminal B like (HER2 pos) |
| TCGA-D8-A141 | Positive | [Not Available] | Positive | [Not Available] | Negative | [Not Available] | 1+ | [Not Evaluated] | Luminal-HER2 neg |
| TCGA-D8-A142 | Negative | [Not Available] | Negative | [Not Available] | Equivocal | [Not Available] | 2+ | Negative | Triple negative |
| TCGA-D8-A143 | Negative | [Not Available] | Negative | [Not Available] | Negative | [Not Available] | 1+ | [Not Evaluated] | Triple negative |
| TCGA-D8-A145 | Positive | [Not Available] | Positive | <10% | Equivocal | [Not Available] | 2+ | Negative | Luminal-HER2 neg |
| TCGA-D8-A146 | Positive | [Not Available] | Positive | [Not Available] | Negative | [Not Available] | 1+ | [Not Evaluated] | Luminal-HER2 neg |
| TCGA-D8-A147 | Negative | [Not Available] | Negative | [Not Available] | Negative | [Not Available] | [Not Available] | [Not Evaluated] | Triple negative |
| TCGA-D8-A1J8 | Positive | 70-79% | Positive | 70-79% | Negative | [Not Available] | [Not Available] | [Not Evaluated] | Luminal-HER2 neg |
| TCGA-D8-A1J9 | Positive | [Not Available] | Negative | [Not Available] | Positive | [Not Available] | 3+ | [Not Evaluated] | Luminal B like (HER2 pos) |
| TCGA-D8-A1JA | Negative | <10% | Negative | [Not Available] | Positive | [Not Available] | 3+ | [Not Evaluated] | HER2-positive (non-luminal) |
| TCGA-D8-A1JB | Positive | [Not Available] | Positive | [Not Available] | Equivocal | [Not Available] | 2+ | Positive | Luminal B like (HER2 pos) |
| TCGA-D8-A1JC | Positive | [Not Available] | Positive | [Not Available] | Negative | [Not Available] | 1+ | [Not Evaluated] | Luminal-HER2 neg |
| TCGA-D8-A1JD | Positive | [Not Available] | Positive | [Not Available] | Negative | [Not Available] | 1+ | [Not Evaluated] | Luminal-HER2 neg |
| TCGA-D8-A1JE | Positive | [Not Available] | Positive | [Not Available] | Negative | [Not Available] | 1+ | [Not Evaluated] | Luminal-HER2 neg |
| TCGA-D8-A1JF | Negative | [Not Available] | Negative | [Not Available] | Negative | [Not Available] | 1+ | [Not Evaluated] | Triple negative |
| TCGA-D8-A1JG | Negative | [Not Available] | Negative | [Not Available] | Equivocal | [Not Available] | 2+ | Negative | Triple negative |
| TCGA-D8-A1JH | Positive | [Not Available] | Positive | [Not Available] | Negative | [Not Available] | 1+ | [Not Evaluated] | Luminal-HER2 neg |
| TCGA-D8-A1JI | Positive | [Not Available] | Positive | [Not Available] | Negative | [Not Available] | [Not Available] | [Not Evaluated] | Luminal-HER2 neg |
| TCGA-D8-A1JJ | Positive | [Not Available] | Positive | [Not Available] | Negative | [Not Available] | 1+ | [Not Evaluated] | Luminal-HER2 neg |
| TCGA-D8-A1JK | Negative | [Not Available] | Positive | <10% | Negative | [Not Available] | 1+ | [Not Evaluated] | Luminal-HER2 neg |
| TCGA-D8-A1JL | Negative | [Not Available] | Negative | [Not Available] | Negative | [Not Available] | 1+ | [Not Evaluated] | Triple negative |
| TCGA-D8-A1JM | Positive | <10% | Negative | [Not Available] | Negative | [Not Available] | 1+ | [Not Evaluated] | Luminal-HER2 neg |
| TCGA-D8-A1JN | Positive | [Not Available] | Positive | <10% | Indeterminate | [Not Available] | [Not Available] | Negative | Luminal-HER2 neg |
| TCGA-D8-A1JP | Positive | [Not Available] | Positive | <10% | Negative | [Not Available] | 1+ | [Not Evaluated] | Luminal-HER2 neg |
| TCGA-D8-A1JS | Positive | [Not Available] | Positive | [Not Available] | Negative | [Not Available] | 1+ | [Not Evaluated] | Luminal-HER2 neg |
| TCGA-D8-A1JT | Positive | 70-79% | Positive | 70-79% | Equivocal | [Not Available] | 2+ | Negative | Luminal-HER2 neg |
| TCGA-D8-A1JU | Positive | [Not Available] | Positive | [Not Available] | Negative | [Not Available] | 1+ | [Not Evaluated] | Luminal-HER2 neg |
| TCGA-D8-A1X5 | Positive | [Not Available] | Positive | [Not Available] | Positive | [Not Available] | 3+ | [Not Evaluated] | Luminal B like (HER2 pos) |
| TCGA-D8-A1X6 | Positive | [Not Available] | Positive | [Not Available] | Negative | [Not Available] | 1+ | [Not Evaluated] | Luminal-HER2 neg |
| TCGA-D8-A1X7 | Positive | [Not Available] | Positive | [Not Available] | Negative | [Not Available] | [Not Available] | [Not Evaluated] | Luminal-HER2 neg |
| TCGA-D8-A1X8 | Positive | [Not Available] | Indeterminate | [Not Available] | Negative | [Not Available] | [Not Available] | [Not Evaluated] | Luminal-HER2 neg |
| TCGA-D8-A1X9 | Positive | [Not Available] | Positive | [Not Available] | Indeterminate | [Not Available] | [Not Available] | Negative | Luminal-HER2 neg |
| TCGA-D8-A1XA | Positive | [Not Available] | Positive | [Not Available] | Equivocal | [Not Available] | 2+ | [Not Evaluated] | Undetermined |
| TCGA-D8-A1XB | Positive | [Not Available] | Positive | [Not Available] | Negative | [Not Available] | 1+ | [Not Evaluated] | Luminal-HER2 neg |
| TCGA-D8-A1XC | Positive | [Not Available] | Positive | [Not Available] | Negative | [Not Available] | 1+ | [Not Evaluated] | Luminal-HER2 neg |
| TCGA-D8-A1XD | Positive | [Not Available] | Positive | [Not Available] | Negative | [Not Available] | 1+ | [Not Evaluated] | Luminal-HER2 neg |
| TCGA-D8-A1XF | Positive | [Not Available] | Positive | [Not Available] | Negative | [Not Available] | 1+ | [Not Evaluated] | Luminal-HER2 neg |
| TCGA-D8-A1XG | Positive | [Not Available] | Negative | [Not Available] | Negative | [Not Available] | [Not Available] | [Not Evaluated] | Luminal-HER2 neg |
| TCGA-D8-A1XJ | Positive | [Not Available] | Positive | [Not Available] | Positive | [Not Available] | 3+ | [Not Evaluated] | Luminal B like (HER2 pos) |
| TCGA-D8-A1XK | Negative | [Not Available] | Negative | [Not Available] | Negative | [Not Available] | [Not Available] | [Not Evaluated] | Triple negative |
| TCGA-D8-A1XL | Positive | [Not Available] | Positive | [Not Available] | Equivocal | [Not Available] | 2+ | Negative | Luminal-HER2 neg |
| TCGA-D8-A1XM | Positive | [Not Available] | Positive | [Not Available] | Negative | [Not Available] | 1+ | [Not Evaluated] | Luminal-HER2 neg |
| TCGA-D8-A1XO | Positive | [Not Available] | Positive | [Not Available] | Negative | [Not Available] | 1+ | [Not Evaluated] | Luminal-HER2 neg |
| TCGA-D8-A1XQ | Negative | [Not Available] | Negative | <10% | Negative | [Not Available] | 1+ | [Not Evaluated] | Triple negative |
| TCGA-D8-A1XR | Positive | [Not Available] | Positive | [Not Available] | Negative | [Not Available] | 1+ | [Not Evaluated] | Luminal-HER2 neg |
| TCGA-D8-A1XS | Positive | [Not Available] | Positive | [Not Available] | Positive | [Not Available] | 3+ | [Not Evaluated] | Luminal B like (HER2 pos) |
| TCGA-D8-A1XT | Negative | [Not Available] | Negative | [Not Available] | Positive | [Not Available] | 3+ | [Not Evaluated] | HER2-positive (non-luminal) |
| TCGA-D8-A1XU | Positive | [Not Available] | Positive | [Not Available] | Negative | [Not Available] | 1+ | [Not Evaluated] | Luminal-HER2 neg |
| TCGA-D8-A1XV | Positive | [Not Available] | Positive | [Not Available] | Equivocal | [Not Available] | 2+ | Negative | Luminal-HER2 neg |
| TCGA-D8-A1XW | Negative | [Not Available] | Positive | <10% | Negative | [Not Available] | 1+ | [Not Evaluated] | Luminal-HER2 neg |
| TCGA-D8-A1XY | Positive | [Not Available] | Positive | [Not Available] | Positive | [Not Available] | 3+ | [Not Evaluated] | Luminal B like (HER2 pos) |
| TCGA-D8-A1XZ | Positive | [Not Available] | Negative | [Not Available] | Negative | [Not Available] | 1+ | [Not Evaluated] | Luminal-HER2 neg |
| TCGA-D8-A1Y0 | Positive | [Not Available] | Positive | [Not Available] | Negative | [Not Available] | 1+ | [Not Evaluated] | Luminal-HER2 neg |
| TCGA-D8-A1Y1 | Positive | [Not Available] | Positive | [Not Available] | Negative | [Not Available] | 1+ | [Not Evaluated] | Luminal-HER2 neg |
| TCGA-D8-A1Y2 | Positive | [Not Available] | Positive | <10% | Indeterminate | [Not Available] | 2+ | Negative | Luminal-HER2 neg |
| TCGA-D8-A1Y3 | Positive | [Not Available] | Positive | [Not Available] | Indeterminate | [Not Available] | 2+ | Negative | Luminal-HER2 neg |
| TCGA-D8-A27E | Positive | [Not Available] | Positive | <10% | Negative | [Not Available] | 1+ | [Not Evaluated] | Luminal-HER2 neg |
| TCGA-D8-A27F | Negative | [Not Available] | Negative | [Not Available] | Negative | [Not Available] | 1+ | [Not Evaluated] | Triple negative |
| TCGA-D8-A27G | Positive | [Not Available] | Positive | [Not Available] | Equivocal | [Not Available] | 2+ | Negative | Luminal-HER2 neg |
| TCGA-D8-A27H | Negative | [Not Available] | Negative | [Not Available] | Negative | [Not Available] | [Not Available] | [Not Evaluated] | Triple negative |
| TCGA-D8-A27I | Positive | [Not Available] | Positive | [Not Available] | Negative | [Not Available] | 1+ | [Not Evaluated] | Luminal-HER2 neg |
| TCGA-D8-A27K | Positive | [Not Available] | Positive | [Not Available] | Negative | [Not Available] | 1+ | [Not Evaluated] | Luminal-HER2 neg |
| TCGA-D8-A27L | Positive | [Not Available] | Positive | [Not Available] | Negative | [Not Available] | 1+ | [Not Evaluated] | Luminal-HER2 neg |
| TCGA-D8-A27M | Negative | [Not Available] | Negative | [Not Available] | Negative | [Not Available] | [Not Available] | [Not Evaluated] | Triple negative |
| TCGA-D8-A27N | Positive | [Not Available] | Positive | [Not Available] | Positive | [Not Available] | 3+ | [Not Evaluated] | Luminal B like (HER2 pos) |
| TCGA-D8-A27P | Positive | [Not Available] | Positive | [Not Available] | Negative | [Not Available] | 1+ | [Not Evaluated] | Luminal-HER2 neg |
| TCGA-D8-A27R | Positive | [Not Available] | Positive | [Not Available] | Equivocal | [Not Available] | 2+ | Negative | Luminal-HER2 neg |
| TCGA-D8-A27T | Positive | [Not Available] | Positive | [Not Available] | Negative | [Not Available] | 1+ | [Not Evaluated] | Luminal-HER2 neg |
| TCGA-D8-A27V | Positive | [Not Available] | Positive | <10% | Negative | [Not Available] | 1+ | [Not Evaluated] | Luminal-HER2 neg |
| TCGA-D8-A27W | Positive | [Not Available] | Positive | [Not Available] | Equivocal | [Not Available] | 2+ | Positive | Luminal B like (HER2 pos) |
| TCGA-D8-A3Z5 | Positive | [Not Available] | Positive | [Not Available] | Negative | [Not Available] | [Not Available] | [Not Evaluated] | Luminal-HER2 neg |
| TCGA-D8-A3Z6 | Positive | [Not Available] | Positive | [Not Available] | Negative | [Not Available] | [Not Available] | [Not Evaluated] | Luminal-HER2 neg |
| TCGA-D8-A4Z1 | Positive | 70-79% | Positive | 10-19% | Negative | [Not Available] | 1+ | [Not Evaluated] | Luminal-HER2 neg |
| TCGA-D8-A73U | Positive | 70-79% | Positive | 70-79% | Negative | <10% | 1+ | Negative | Luminal-HER2 neg |
| TCGA-D8-A73W | Positive | 70-79% | Negative | [Not Available] | Negative | [Not Available] | 1+ | [Not Evaluated] | Luminal-HER2 neg |
| TCGA-D8-A73X | Positive | 70-79% | Positive | 70-79% | Negative | [Not Available] | 1+ | Negative | Luminal-HER2 neg |
| TCGA-E2-A105 | Positive | [Not Available] | Positive | [Not Available] | Equivocal | 70-79% | 2+ | [Not Evaluated] | Undetermined |
| TCGA-E2-A106 | Positive | 90-99% | Positive | 80-89% | Equivocal | 10-19% | 2+ | Negative | Luminal-HER2 neg |
| TCGA-E2-A107 | Positive | [Not Available] | Negative | [Not Available] | Equivocal | [Not Available] | 2+ | Negative | Luminal-HER2 neg |
| TCGA-E2-A108 | Positive | [Not Available] | Positive | [Not Available] | Negative | [Not Available] | 1+ | [Not Evaluated] | Luminal-HER2 neg |
| TCGA-E2-A109 | Positive | [Not Available] | Negative | [Not Available] | Equivocal | 50-59% | 2+ | Negative | Luminal-HER2 neg |
| TCGA-E2-A10A | Positive | [Not Available] | Positive | [Not Available] | Positive | 40-49% | 2+ | Negative | Luminal-HER2 neg |
| TCGA-E2-A10B | Positive | [Not Available] | Positive | [Not Available] | Equivocal | 10-19% | 2+ | Negative | Luminal-HER2 neg |
| TCGA-E2-A10C | Positive | [Not Available] | Positive | [Not Available] | Negative | <10% | 1+ | [Not Evaluated] | Luminal-HER2 neg |
| TCGA-E2-A10E | Positive | [Not Available] | Positive | [Not Available] | Equivocal | 40-49% | 2+ | Negative | Luminal-HER2 neg |
| TCGA-E2-A10F | Positive | [Not Available] | Positive | [Not Available] | Equivocal | 20-29% | 2+ | Negative | Luminal-HER2 neg |
| TCGA-E2-A14N | Negative | [Not Available] | Negative | [Not Available] | Negative | <10% | [Not Available] | [Not Evaluated] | Triple negative |
| TCGA-E2-A14O | Positive | [Not Available] | Positive | [Not Available] | Equivocal | 50-59% | 2+ | Negative | Luminal-HER2 neg |
| TCGA-E2-A14P | Negative | [Not Available] | Negative | [Not Available] | Positive | 90-99% | 3+ | [Not Evaluated] | HER2-positive (non-luminal) |
| TCGA-E2-A14Q | Positive | [Not Available] | Positive | [Not Available] | Negative | <10% | 1+ | [Not Evaluated] | Luminal-HER2 neg |
| TCGA-E2-A14R | Negative | [Not Available] | Negative | [Not Available] | Negative | <10% | 1+ | [Not Evaluated] | Triple negative |
| TCGA-E2-A14S | Positive | [Not Available] | Positive | [Not Available] | Equivocal | [Not Available] | 2+ | Negative | Luminal-HER2 neg |
| TCGA-E2-A14T | Positive | [Not Available] | Positive | [Not Available] | Negative | [Not Available] | 1+ | Negative | Luminal-HER2 neg |
| TCGA-E2-A14U | Positive | [Not Available] | Positive | [Not Available] | Equivocal | 40-49% | 2+ | Negative | Luminal-HER2 neg |
| TCGA-E2-A14V | Positive | [Not Available] | Positive | [Not Available] | Positive | 90-99% | 3+ | [Not Evaluated] | Luminal B like (HER2 pos) |
| TCGA-E2-A14W | Positive | [Not Available] | Positive | [Not Available] | [Not Evaluated] | [Not Available] | [Not Available] | Positive | Luminal B like (HER2 pos) |
| TCGA-E2-A14X | Negative | [Not Available] | Negative | [Not Available] | Negative | <10% | 1+ | [Not Evaluated] | Triple negative |
| TCGA-E2-A14Y | Positive | [Not Available] | Positive | [Not Available] | Equivocal | [Not Available] | 2+ | Positive | Luminal B like (HER2 pos) |
| TCGA-E2-A14Z | Positive | [Not Available] | Positive | [Not Available] | Negative | <10% | 1+ | [Not Evaluated] | Luminal-HER2 neg |
| TCGA-E2-A150 | Negative | [Not Available] | Negative | [Not Available] | Negative | [Not Available] | [Not Available] | [Not Evaluated] | Triple negative |
| TCGA-E2-A152 | Positive | [Not Available] | Negative | [Not Available] | Positive | [Not Available] | 3+ | [Not Evaluated] | Luminal B like (HER2 pos) |
| TCGA-E2-A153 | Positive | [Not Available] | Positive | [Not Available] | Negative | [Not Available] | 1+ | [Not Evaluated] | Luminal-HER2 neg |
| TCGA-E2-A154 | Positive | 90-99% | Positive | 90-99% | Negative | [Not Available] | 1+ | [Not Evaluated] | Luminal-HER2 neg |
| TCGA-E2-A155 | Positive | [Not Available] | Negative | [Not Available] | Negative | [Not Available] | [Not Available] | [Not Evaluated] | Luminal-HER2 neg |
| TCGA-E2-A156 | Positive | [Not Available] | Positive | [Not Available] | Negative | [Not Available] | [Not Available] | [Not Evaluated] | Luminal-HER2 neg |
| TCGA-E2-A158 | Negative | [Not Available] | Negative | [Not Available] | Negative | <10% | 1+ | [Not Evaluated] | Triple negative |
| TCGA-E2-A159 | Negative | [Not Available] | Negative | [Not Available] | [Not Evaluated] | [Not Available] | [Not Available] | Negative | Triple negative |
| TCGA-E2-A15A | Positive | [Not Available] | Positive | [Not Available] | Negative | [Not Available] | 1+ | [Not Evaluated] | Luminal-HER2 neg |
| TCGA-E2-A15C | Positive | [Not Available] | Positive | [Not Available] | Equivocal | 20-29% | 2+ | Negative | Luminal-HER2 neg |
| TCGA-E2-A15D | Positive | [Not Available] | Positive | [Not Available] | Equivocal | [Not Available] | 2+ | Negative | Luminal-HER2 neg |
| TCGA-E2-A15E | Positive | [Not Available] | Positive | [Not Available] | [Not Evaluated] | [Not Available] | [Not Available] | Positive | Luminal B like (HER2 pos) |
| TCGA-E2-A15F | Positive | 90-99% | Positive | 90-99% | Negative | [Not Available] | 1+ | [Not Evaluated] | Luminal-HER2 neg |
| TCGA-E2-A15G | Positive | [Not Available] | Positive | [Not Available] | Negative | [Not Available] | 1+ | [Not Evaluated] | Luminal-HER2 neg |
| TCGA-E2-A15H | Positive | [Not Available] | Positive | [Not Available] | Equivocal | [Not Available] | 2+ | Positive | Luminal B like (HER2 pos) |
| TCGA-E2-A15I | Positive | 90-99% | Positive | 90-99% | Equivocal | [Not Available] | 2+ | Negative | Luminal-HER2 neg |
| TCGA-E2-A15J | Positive | [Not Available] | Positive | [Not Available] | Equivocal | [Not Available] | 2+ | Negative | Luminal-HER2 neg |
| TCGA-E2-A15K | Positive | [Not Available] | Positive | [Not Available] | Equivocal | 10-19% | 2+ | Negative | Luminal-HER2 neg |
| TCGA-E2-A15L | Positive | [Not Available] | Positive | [Not Available] | Equivocal | 30-39% | 2+ | Negative | Luminal-HER2 neg |
| TCGA-E2-A15M | Positive | [Not Available] | Positive | [Not Available] | Negative | [Not Available] | [Not Available] | [Not Evaluated] | Luminal-HER2 neg |
| TCGA-E2-A15O | Positive | [Not Available] | Positive | [Not Available] | Equivocal | 10-19% | 2+ | Negative | Luminal-HER2 neg |
| TCGA-E2-A15P | Positive | [Not Available] | Positive | [Not Available] | Negative | [Not Available] | 1+ | [Not Evaluated] | Luminal-HER2 neg |
| TCGA-E2-A15R | Positive | [Not Available] | Positive | [Not Available] | Equivocal | [Not Available] | 2+ | Negative | Luminal-HER2 neg |
| TCGA-E2-A15S | Positive | [Not Available] | Negative | [Not Available] | Equivocal | [Not Available] | 2+ | Negative | Luminal-HER2 neg |
| TCGA-E2-A15T | Positive | [Not Available] | Positive | [Not Available] | Equivocal | [Not Available] | 2+ | Negative | Luminal-HER2 neg |
| TCGA-E2-A1AZ | Negative | [Not Available] | Negative | [Not Available] | Indeterminate | [Not Available] | [Not Available] | Negative | Triple negative |
| TCGA-E2-A1B0 | Negative | [Not Available] | Negative | [Not Available] | Positive | 90-99% | 3+ | [Not Evaluated] | HER2-positive (non-luminal) |
| TCGA-E2-A1B1 | Positive | [Not Available] | Positive | [Not Available] | Equivocal | 70-79% | 2+ | Positive | Luminal B like (HER2 pos) |
| TCGA-E2-A1B4 | Positive | [Not Available] | Positive | [Not Available] | Negative | 10-19% | 1+ | [Not Evaluated] | Luminal-HER2 neg |
| TCGA-E2-A1B5 | Positive | [Not Available] | Positive | [Not Available] | Negative | <10% | [Not Available] | [Not Evaluated] | Luminal-HER2 neg |
| TCGA-E2-A1B6 | Negative | [Not Available] | Negative | [Not Available] | Equivocal | 10-19% | 2+ | Negative | Triple negative |
| TCGA-E2-A1BC | Positive | [Not Available] | Positive | [Not Available] | Negative | [Not Available] | [Not Available] | [Not Evaluated] | Luminal-HER2 neg |
| TCGA-E2-A1BD | Positive | [Not Available] | Positive | [Not Available] | Equivocal | 10-19% | 2+ | Negative | Luminal-HER2 neg |
| TCGA-E2-A1IE | Positive | [Not Available] | Positive | [Not Available] | Equivocal | 20-29% | 2+ | Negative | Luminal-HER2 neg |
| TCGA-E2-A1IF | Positive | [Not Available] | Positive | [Not Available] | Negative | <10% | 1+ | [Not Evaluated] | Luminal-HER2 neg |
| TCGA-E2-A1IG | Positive | [Not Available] | Positive | [Not Available] | Negative | [Not Available] | [Not Available] | [Not Evaluated] | Luminal-HER2 neg |
| TCGA-E2-A1IH | Positive | [Not Available] | Positive | [Not Available] | Equivocal | 20-29% | 2+ | [Not Evaluated] | Undetermined |
| TCGA-E2-A1II | Negative | [Not Available] | Positive | [Not Available] | Negative | <10% | 1+ | [Not Evaluated] | Luminal-HER2 neg |
| TCGA-E2-A1IJ | Positive | 90-99% | Positive | 90-99% | Equivocal | [Not Available] | 2+ | Negative | Luminal-HER2 neg |
| TCGA-E2-A1IK | Positive | [Not Available] | Positive | [Not Available] | Negative | [Not Available] | [Not Available] | [Not Evaluated] | Luminal-HER2 neg |
| TCGA-E2-A1IL | Positive | [Not Available] | Positive | [Not Available] | Negative | [Not Available] | [Not Available] | [Not Evaluated] | Luminal-HER2 neg |
| TCGA-E2-A1IN | Positive | [Not Available] | Positive | [Not Available] | Negative | [Not Available] | 1+ | [Not Evaluated] | Luminal-HER2 neg |
| TCGA-E2-A1IO | Positive | [Not Available] | Positive | [Not Available] | Equivocal | 30-39% | 2+ | Negative | Luminal-HER2 neg |
| TCGA-E2-A1IP | Positive | [Not Available] | Negative | [Not Available] | Negative | [Not Available] | 1+ | Not Performed | Luminal-HER2 neg |
| TCGA-E2-A1IU | Positive | [Not Available] | Positive | [Not Available] | Negative | [Not Available] | [Not Available] | [Not Evaluated] | Luminal-HER2 neg |
| TCGA-E2-A1L6 | Positive | [Not Available] | Positive | [Not Available] | Negative | 40-49% | 1+ | [Not Evaluated] | Luminal-HER2 neg |
| TCGA-E2-A1L7 | Negative | [Not Available] | Negative | [Not Available] | Negative | [Not Available] | [Not Available] | [Not Evaluated] | Triple negative |
| TCGA-E2-A1L8 | Positive | [Not Available] | Positive | [Not Available] | Equivocal | 40-49% | 2+ | Negative | Luminal-HER2 neg |
| TCGA-E2-A1L9 | Positive | 80-89% | Positive | 80-89% | Negative | [Not Available] | [Not Available] | [Not Evaluated] | Luminal-HER2 neg |
| TCGA-E2-A1LA | Positive | [Not Available] | Positive | [Not Available] | Equivocal | [Not Available] | 2+ | Negative | Luminal-HER2 neg |
| TCGA-E2-A1LB | Negative | [Not Available] | Negative | [Not Available] | Positive | 90-99% | 3+ | [Not Evaluated] | HER2-positive (non-luminal) |
| TCGA-E2-A1LE | Negative | [Not Available] | Negative | [Not Available] | Positive | 90-99% | 3+ | [Not Evaluated] | HER2-positive (non-luminal) |
| TCGA-E2-A1LG | Negative | [Not Available] | Negative | [Not Available] | Equivocal | 60-69% | 2+ | Negative | Triple negative |
| TCGA-E2-A1LH | Negative | [Not Available] | Negative | [Not Available] | Negative | [Not Available] | [Not Available] | [Not Evaluated] | Triple negative |
| TCGA-E2-A1LI | Negative | [Not Available] | Negative | [Not Available] | Equivocal | [Not Available] | [Not Available] | Negative | Triple negative |
| TCGA-E2-A1LK | Negative | [Not Available] | Negative | [Not Available] | Equivocal | 80-89% | 2+ | Negative | Triple negative |
| TCGA-E2-A1LL | Negative | [Not Available] | Negative | [Not Available] | Negative | <10% | 1+ | [Not Evaluated] | Triple negative |
| TCGA-E2-A1LS | Negative | [Not Available] | Negative | [Not Available] | Negative | [Not Available] | 1+ | [Not Evaluated] | Triple negative |
| TCGA-E2-A2P5 | Positive | [Not Available] | Positive | [Not Available] | [Not Evaluated] | [Not Available] | [Not Available] | Negative | Luminal-HER2 neg |
| TCGA-E2-A2P6 | Positive | [Not Available] | Positive | [Not Available] | [Not Evaluated] | [Not Available] | [Not Available] | Negative | Luminal-HER2 neg |
| TCGA-E2-A3DX | Positive | [Not Available] | Positive | [Not Available] | [Not Evaluated] | [Not Available] | [Not Available] | Positive | Luminal B like (HER2 pos) |
| TCGA-E2-A56Z | Positive | 10-19% | Positive | <10% | Equivocal | 10-19% | 2+ | Negative | Luminal-HER2 neg |
| TCGA-E2-A570 | Positive | [Not Available] | Positive | [Not Available] | Equivocal | 10-19% | 2+ | Negative | Luminal-HER2 neg |
| TCGA-E2-A572 | Positive | [Not Available] | Positive | [Not Available] | Equivocal | [Not Available] | [Not Available] | Negative | Luminal-HER2 neg |
| TCGA-E2-A573 | Negative | [Not Available] | Negative | [Not Available] | Equivocal | 10-19% | 2+ | Negative | Triple negative |
| TCGA-E2-A574 | Negative | [Not Available] | Negative | [Not Available] | Equivocal | 10-19% | 2+ | Negative | Triple negative |
| TCGA-E2-A576 | Positive | [Not Available] | Positive | [Not Available] | Equivocal | [Not Available] | 2+ | Negative | Luminal-HER2 neg |
| TCGA-E2-A9RU | Positive | 70-79% | Negative | [Not Available] | Equivocal | [Not Available] | 2+ | Negative | Luminal-HER2 neg |
| TCGA-E9-A1N3 | [Not Evaluated] | [Not Available] | [Not Evaluated] | [Not Available] | [Not Evaluated] | [Not Available] | [Not Available] | [Not Evaluated] | Undetermined |
| TCGA-E9-A1N4 | Positive | [Not Available] | Positive | [Not Available] | Positive | [Not Available] | [Not Available] | [Not Evaluated] | Luminal B like (HER2 pos) |
| TCGA-E9-A1N5 | Positive | [Not Available] | Positive | [Not Available] | Positive | [Not Available] | [Not Available] | [Not Evaluated] | Luminal B like (HER2 pos) |
| TCGA-E9-A1N6 | Positive | [Not Available] | Positive | [Not Available] | Positive | [Not Available] | [Not Available] | [Not Evaluated] | Luminal B like (HER2 pos) |
| TCGA-E9-A1N8 | Negative | [Not Available] | [Not Evaluated] | [Not Available] | Negative | [Not Available] | [Not Available] | [Not Evaluated] | Undetermined |
| TCGA-E9-A1N9 | Negative | [Not Available] | Positive | [Not Available] | Positive | [Not Available] | [Not Available] | [Not Evaluated] | Undetermined |
| TCGA-E9-A1NA | Positive | [Not Available] | Positive | [Not Available] | Positive | [Not Available] | [Not Available] | [Not Evaluated] | Luminal B like (HER2 pos) |
| TCGA-E9-A1NC | Negative | [Not Available] | Positive | [Not Available] | Positive | [Not Available] | [Not Available] | [Not Evaluated] | Undetermined |
| TCGA-E9-A1ND | Negative | [Not Available] | Negative | [Not Available] | Positive | [Not Available] | [Not Available] | [Not Evaluated] | HER2-positive (non-luminal) |
| TCGA-E9-A1NE | Positive | [Not Available] | Positive | [Not Available] | Negative | [Not Available] | [Not Available] | [Not Evaluated] | Luminal-HER2 neg |
| TCGA-E9-A1NF | Positive | [Not Available] | Positive | [Not Available] | Negative | [Not Available] | [Not Available] | [Not Evaluated] | Luminal-HER2 neg |
| TCGA-E9-A1NG | Positive | [Not Available] | Positive | [Not Available] | Negative | [Not Available] | [Not Available] | [Not Evaluated] | Luminal-HER2 neg |
| TCGA-E9-A1NH | Positive | [Not Available] | Positive | [Not Available] | Negative | [Not Available] | [Not Available] | [Not Evaluated] | Luminal-HER2 neg |
| TCGA-E9-A1NI | Positive | [Not Available] | Positive | [Not Available] | Negative | [Not Available] | [Not Available] | [Not Evaluated] | Luminal-HER2 neg |
| TCGA-E9-A1QZ | [Not Evaluated] | [Not Available] | [Not Evaluated] | [Not Available] | [Not Evaluated] | [Not Available] | [Not Available] | [Not Evaluated] | Undetermined |
| TCGA-E9-A1R0 | [Not Evaluated] | [Not Available] | [Not Evaluated] | [Not Available] | [Not Evaluated] | [Not Available] | [Not Available] | [Not Evaluated] | Undetermined |
| TCGA-E9-A1R2 | Positive | 90-99% | Negative | [Not Available] | Equivocal | 90-99% | [Not Available] | Negative | Luminal-HER2 neg |
| TCGA-E9-A1R3 | [Not Evaluated] | [Not Available] | [Not Evaluated] | [Not Available] | [Not Evaluated] | [Not Available] | [Not Available] | [Not Evaluated] | Undetermined |
| TCGA-E9-A1R4 | [Not Evaluated] | [Not Available] | [Not Evaluated] | [Not Available] | [Not Evaluated] | [Not Available] | [Not Available] | [Not Evaluated] | Undetermined |
| TCGA-E9-A1R5 | [Not Evaluated] | [Not Available] | [Not Evaluated] | [Not Available] | [Not Evaluated] | [Not Available] | [Not Available] | [Not Evaluated] | Undetermined |
| TCGA-E9-A1R6 | [Not Evaluated] | [Not Available] | [Not Evaluated] | [Not Available] | [Not Evaluated] | [Not Available] | [Not Available] | [Not Evaluated] | Undetermined |
| TCGA-E9-A1R7 | [Not Evaluated] | [Not Available] | [Not Evaluated] | [Not Available] | [Not Evaluated] | [Not Available] | [Not Available] | [Not Evaluated] | Undetermined |
| TCGA-E9-A1RA | [Not Evaluated] | [Not Available] | [Not Evaluated] | [Not Available] | [Not Evaluated] | [Not Available] | [Not Available] | [Not Evaluated] | Undetermined |
| TCGA-E9-A1RB | [Not Evaluated] | [Not Available] | [Not Evaluated] | [Not Available] | [Not Evaluated] | [Not Available] | [Not Available] | [Not Evaluated] | Undetermined |
| TCGA-E9-A1RC | [Not Evaluated] | [Not Available] | [Not Evaluated] | [Not Available] | [Not Evaluated] | [Not Available] | [Not Available] | [Not Evaluated] | Undetermined |
| TCGA-E9-A1RD | [Not Evaluated] | [Not Available] | [Not Evaluated] | [Not Available] | [Not Evaluated] | [Not Available] | [Not Available] | [Not Evaluated] | Undetermined |
| TCGA-E9-A1RE | [Not Evaluated] | [Not Available] | [Not Evaluated] | [Not Available] | [Not Evaluated] | [Not Available] | [Not Available] | [Not Evaluated] | Undetermined |
| TCGA-E9-A1RF | [Not Evaluated] | [Not Available] | [Not Evaluated] | [Not Available] | [Not Evaluated] | [Not Available] | [Not Available] | [Not Evaluated] | Undetermined |
| TCGA-E9-A1RG | [Not Evaluated] | [Not Available] | [Not Evaluated] | [Not Available] | [Not Evaluated] | [Not Available] | [Not Available] | [Not Evaluated] | Undetermined |
| TCGA-E9-A1RH | [Not Evaluated] | [Not Available] | [Not Evaluated] | [Not Available] | [Not Evaluated] | [Not Available] | [Not Available] | [Not Evaluated] | Undetermined |
| TCGA-E9-A1RI | [Not Evaluated] | [Not Available] | [Not Evaluated] | [Not Available] | [Not Evaluated] | [Not Available] | [Not Available] | [Not Evaluated] | Undetermined |
| TCGA-E9-A226 | [Not Evaluated] | [Not Available] | [Not Evaluated] | [Not Available] | [Not Evaluated] | [Not Available] | [Not Available] | [Not Evaluated] | Undetermined |
| TCGA-E9-A227 | Positive | 90-99% | Positive | 80-89% | Negative | [Not Available] | [Not Available] | [Not Evaluated] | Luminal-HER2 neg |
| TCGA-E9-A228 | [Not Evaluated] | [Not Available] | [Not Evaluated] | [Not Available] | [Not Evaluated] | [Not Available] | [Not Available] | [Not Evaluated] | Undetermined |
| TCGA-E9-A229 | [Not Evaluated] | [Not Available] | [Not Evaluated] | [Not Available] | [Not Evaluated] | [Not Available] | [Not Available] | [Not Evaluated] | Undetermined |
| TCGA-E9-A22A | Positive | 10-19% | Negative | [Not Available] | Negative | [Not Available] | [Not Available] | [Not Evaluated] | Luminal-HER2 neg |
| TCGA-E9-A22B | Positive | 10-19% | Negative | [Not Available] | Negative | [Not Available] | [Not Available] | [Not Evaluated] | Luminal-HER2 neg |
| TCGA-E9-A22D | Positive | 10-19% | Positive | <10% | Positive | <10% | [Not Available] | [Not Evaluated] | Luminal B like (HER2 pos) |
| TCGA-E9-A22E | Positive | 10-19% | Positive | <10% | Positive | <10% | [Not Available] | [Not Evaluated] | Luminal B like (HER2 pos) |
| TCGA-E9-A22G | Negative | [Not Available] | Negative | [Not Available] | Positive | <10% | [Not Available] | [Not Evaluated] | HER2-positive (non-luminal) |
| TCGA-E9-A22H | Positive | <10% | Positive | 10-19% | Positive | <10% | [Not Available] | [Not Evaluated] | Luminal B like (HER2 pos) |
| TCGA-E9-A243 | [Not Evaluated] | [Not Available] | [Not Evaluated] | [Not Available] | [Not Evaluated] | [Not Available] | [Not Available] | [Not Evaluated] | Undetermined |
| TCGA-E9-A244 | [Not Evaluated] | [Not Available] | [Not Evaluated] | [Not Available] | [Not Evaluated] | [Not Available] | [Not Available] | [Not Evaluated] | Undetermined |
| TCGA-E9-A245 | [Not Evaluated] | [Not Available] | [Not Evaluated] | [Not Available] | [Not Evaluated] | [Not Available] | [Not Available] | [Not Evaluated] | Undetermined |
| TCGA-E9-A247 | [Not Evaluated] | [Not Available] | [Not Evaluated] | [Not Available] | [Not Evaluated] | [Not Available] | [Not Available] | [Not Evaluated] | Undetermined |
| TCGA-E9-A248 | [Not Evaluated] | [Not Available] | [Not Evaluated] | [Not Available] | [Not Evaluated] | [Not Available] | [Not Available] | [Not Evaluated] | Undetermined |
| TCGA-E9-A249 | [Not Evaluated] | [Not Available] | [Not Evaluated] | [Not Available] | [Not Evaluated] | [Not Available] | [Not Available] | [Not Evaluated] | Undetermined |
| TCGA-E9-A24A | [Not Evaluated] | [Not Available] | [Not Evaluated] | [Not Available] | [Not Evaluated] | [Not Available] | [Not Available] | [Not Evaluated] | Undetermined |
| TCGA-E9-A295 | Positive | 80-89% | Positive | 80-89% | Positive | [Not Available] | 1+ | [Not Evaluated] | Luminal B like (HER2 pos) |
| TCGA-E9-A2JS | [Not Evaluated] | [Not Available] | [Not Evaluated] | [Not Available] | [Not Evaluated] | [Not Available] | [Not Available] | [Not Evaluated] | Undetermined |
| TCGA-E9-A2JT | [Not Evaluated] | [Not Available] | [Not Evaluated] | [Not Available] | [Not Evaluated] | [Not Available] | [Not Available] | [Not Evaluated] | Undetermined |
| TCGA-E9-A3HO | [Not Evaluated] | [Not Available] | [Not Evaluated] | [Not Available] | [Not Evaluated] | [Not Available] | [Not Available] | [Not Evaluated] | Undetermined |
| TCGA-E9-A3Q9 | Positive | 40-49% | Positive | 40-49% | Indeterminate | <10% | [Not Available] | [Not Evaluated] | Undetermined |
| TCGA-E9-A3QA | [Not Evaluated] | [Not Available] | [Not Evaluated] | [Not Available] | [Not Evaluated] | [Not Available] | [Not Available] | [Not Evaluated] | Undetermined |
| TCGA-E9-A3X8 | Positive | 20-29% | Positive | 20-29% | Positive | <10% | [Not Available] | [Not Evaluated] | Luminal B like (HER2 pos) |
| TCGA-E9-A54X | Positive | 90-99% | Positive | 90-99% | Negative | [Not Available] | [Not Available] | [Not Evaluated] | Luminal-HER2 neg |
| TCGA-E9-A54Y | Positive | 90-99% | Positive | 50-59% | Negative | [Not Available] | 1+ | [Not Evaluated] | Luminal-HER2 neg |
| TCGA-E9-A5FK | Positive | 80-89% | Positive | 70-79% | Negative | [Not Available] | [Not Available] | [Not Evaluated] | Luminal-HER2 neg |
| TCGA-E9-A5FL | Negative | [Not Available] | Negative | [Not Available] | Negative | [Not Available] | [Not Available] | [Not Evaluated] | Triple negative |
| TCGA-E9-A5UO | [Not Evaluated] | [Not Available] | [Not Evaluated] | [Not Available] | [Not Evaluated] | [Not Available] | [Not Available] | [Not Evaluated] | Undetermined |
| TCGA-E9-A5UP | [Not Evaluated] | [Not Available] | [Not Evaluated] | [Not Available] | [Not Evaluated] | [Not Available] | [Not Available] | [Not Evaluated] | Undetermined |
| TCGA-E9-A6HE | Positive | 90-99% | Positive | 90-99% | Negative | <10% | 1+ | Negative | Luminal-HER2 neg |
| TCGA-EW-A1IW | Positive | 90-99% | Positive | 90-99% | Positive | 90-99% | 3+ | [Not Evaluated] | Luminal B like (HER2 pos) |
| TCGA-EW-A1IX | Positive | 90-99% | Positive | 90-99% | Negative | <10% | [Not Available] | [Not Evaluated] | Luminal-HER2 neg |
| TCGA-EW-A1IY | Positive | 90-99% | Positive | 90-99% | Negative | <10% | [Not Available] | [Not Evaluated] | Luminal-HER2 neg |
| TCGA-EW-A1IZ | Positive | 90-99% | Positive | 90-99% | Negative | <10% | [Not Available] | [Not Evaluated] | Luminal-HER2 neg |
| TCGA-EW-A1J1 | Positive | 90-99% | Positive | 90-99% | Negative | <10% | [Not Available] | [Not Evaluated] | Luminal-HER2 neg |
| TCGA-EW-A1J2 | Positive | 90-99% | Positive | 90-99% | Negative | <10% | [Not Available] | [Not Evaluated] | Luminal-HER2 neg |
| TCGA-EW-A1J3 | Positive | 90-99% | Positive | 90-99% | Positive | 90-99% | 3+ | [Not Evaluated] | Luminal B like (HER2 pos) |
| TCGA-EW-A1J5 | Positive | 90-99% | Positive | 90-99% | Negative | <10% | [Not Available] | [Not Evaluated] | Luminal-HER2 neg |
| TCGA-EW-A1J6 | Positive | 90-99% | Positive | 90-99% | Negative | <10% | [Not Available] | [Not Evaluated] | Luminal-HER2 neg |
| TCGA-EW-A1OV | Negative | <10% | Negative | <10% | Negative | <10% | [Not Available] | Negative | Triple negative |
| TCGA-EW-A1OW | Negative | <10% | Negative | <10% | Negative | <10% | 1+ | [Not Evaluated] | Triple negative |
| TCGA-EW-A1OX | Positive | 90-99% | Positive | 90-99% | Negative | <10% | [Not Available] | [Not Evaluated] | Luminal-HER2 neg |
| TCGA-EW-A1OY | Positive | 90-99% | Positive | 90-99% | Negative | <10% | [Not Available] | Negative | Luminal-HER2 neg |
| TCGA-EW-A1OZ | Positive | 90-99% | Negative | <10% | Equivocal | 20-29% | 2+ | Positive | Luminal B like (HER2 pos) |
| TCGA-EW-A1P0 | Positive | 90-99% | Negative | <10% | Negative | <10% | [Not Available] | [Not Evaluated] | Luminal-HER2 neg |
| TCGA-EW-A1P1 | Negative | <10% | Negative | <10% | Equivocal | <10% | 2+ | Negative | Triple negative |
| TCGA-EW-A1P3 | Positive | 90-99% | Positive | 90-99% | Negative | <10% | [Not Available] | [Not Evaluated] | Luminal-HER2 neg |
| TCGA-EW-A1P4 | Negative | <10% | Negative | <10% | Negative | <10% | [Not Available] | [Not Evaluated] | Triple negative |
| TCGA-EW-A1P5 | Positive | 90-99% | Positive | 90-99% | Negative | <10% | [Not Available] | Negative | Luminal-HER2 neg |
| TCGA-EW-A1P6 | Positive | 90-99% | Positive | 90-99% | Negative | <10% | [Not Available] | [Not Evaluated] | Luminal-HER2 neg |
| TCGA-EW-A1P7 | Negative | <10% | Negative | <10% | Equivocal | 10-19% | 2+ | Negative | Triple negative |
| TCGA-EW-A1P8 | Negative | <10% | Negative | <10% | Negative | <10% | [Not Available] | [Not Evaluated] | Triple negative |
| TCGA-EW-A1PA | Positive | 90-99% | Positive | 90-99% | Negative | <10% | 1+ | [Not Evaluated] | Luminal-HER2 neg |
| TCGA-EW-A1PB | Negative | <10% | Negative | <10% | Negative | <10% | [Not Available] | [Not Evaluated] | Triple negative |
| TCGA-EW-A1PC | Positive | 90-99% | Positive | 50-59% | Negative | <10% | 1+ | [Not Evaluated] | Luminal-HER2 neg |
| TCGA-EW-A1PD | Positive | 90-99% | Positive | 90-99% | Equivocal | 20-29% | 2+ | Positive | Luminal B like (HER2 pos) |
| TCGA-EW-A1PE | Positive | 90-99% | Positive | 90-99% | Negative | <10% | [Not Available] | [Not Evaluated] | Luminal-HER2 neg |
| TCGA-EW-A1PF | Positive | 90-99% | Positive | 90-99% | Negative | <10% | [Not Available] | [Not Evaluated] | Luminal-HER2 neg |
| TCGA-EW-A1PG | Positive | 90-99% | Positive | 90-99% | Equivocal | 10-19% | 2+ | Negative | Luminal-HER2 neg |
| TCGA-EW-A1PH | Negative | <10% | Negative | <10% | Negative | <10% | [Not Available] | [Not Evaluated] | Triple negative |
| TCGA-EW-A2FR | Negative | <10% | Negative | <10% | Equivocal | 10-19% | 2+ | Positive | HER2-positive (non-luminal) |
| TCGA-EW-A2FS | Positive | 90-99% | Negative | <10% | Negative | <10% | 1+ | [Not Evaluated] | Luminal-HER2 neg |
| TCGA-EW-A2FV | Positive | 90-99% | Positive | 90-99% | Negative | <10% | 1+ | [Not Evaluated] | Luminal-HER2 neg |
| TCGA-EW-A2FW | Positive | 90-99% | Positive | 90-99% | Negative | <10% | 1+ | [Not Evaluated] | Luminal-HER2 neg |
| TCGA-EW-A3E8 | Positive | [Not Available] | Negative | [Not Available] | Negative | [Not Available] | [Not Available] | [Not Evaluated] | Luminal-HER2 neg |
| TCGA-EW-A3U0 | Negative | [Not Available] | Negative | [Not Available] | Negative | [Not Available] | [Not Available] | [Not Evaluated] | Triple negative |
| TCGA-EW-A423 | Positive | 50-59% | Positive | <10% | Negative | [Not Available] | [Not Available] | [Not Evaluated] | Luminal-HER2 neg |
| TCGA-EW-A424 | Positive | 50-59% | Positive | [Not Available] | Positive | [Not Available] | [Not Available] | [Not Evaluated] | Luminal B like (HER2 pos) |
| TCGA-GI-A2C8 | Positive | 90-99% | Positive | <10% | Negative | [Not Available] | [Not Available] | Negative | Luminal-HER2 neg |
| TCGA-GI-A2C9 | Negative | [Not Available] | Negative | [Not Available] | Negative | [Not Available] | [Not Available] | [Not Evaluated] | Triple negative |
| TCGA-GM-A2D9 | Positive | 90-99% | Positive | 40-49% | [Not Evaluated] | [Not Available] | [Not Available] | Negative | Luminal-HER2 neg |
| TCGA-GM-A2DA | Positive | 90-99% | Positive | 50-59% | Equivocal | 90-99% | 2+ | Positive | Luminal B like (HER2 pos) |
| TCGA-GM-A2DB | Negative | <10% | Negative | <10% | Negative | [Not Available] | 1+ | Negative | Triple negative |
| TCGA-GM-A2DC | Positive | 90-99% | Positive | 90-99% | [Not Evaluated] | [Not Available] | [Not Available] | Negative | Luminal-HER2 neg |
| TCGA-GM-A2DD | Negative | <10% | Negative | <10% | Equivocal | <10% | 2+ | Negative | Triple negative |
| TCGA-GM-A2DF | Negative | <10% | Negative | <10% | Negative | [Not Available] | 1+ | Negative | Triple negative |
| TCGA-GM-A2DH | Negative | <10% | Negative | <10% | Negative | [Not Available] | 1+ | Negative | Triple negative |
| TCGA-GM-A2DI | Negative | <10% | Negative | <10% | [Not Evaluated] | [Not Available] | [Not Available] | Negative | Triple negative |
| TCGA-GM-A2DK | Positive | 90-99% | Negative | <10% | Negative | [Not Available] | [Not Available] | [Not Evaluated] | Luminal-HER2 neg |
| TCGA-GM-A2DL | Positive | 90-99% | Positive | 90-99% | Negative | <10% | 1+ | [Not Evaluated] | Luminal-HER2 neg |
| TCGA-GM-A2DM | Positive | 40-49% | Positive | 30-39% | Negative | <10% | 1+ | [Not Evaluated] | Luminal-HER2 neg |
| TCGA-GM-A2DN | Positive | 90-99% | Positive | 90-99% | Negative | [Not Available] | 1+ | [Not Evaluated] | Luminal-HER2 neg |
| TCGA-GM-A2DO | Positive | 90-99% | Positive | 90-99% | Negative | <10% | [Not Available] | [Not Evaluated] | Luminal-HER2 neg |
| TCGA-GM-A3NW | Negative | [Not Available] | Positive | 20-29% | Equivocal | 30-39% | 1+ | Negative | Luminal-HER2 neg |
| TCGA-GM-A3NY | Positive | 90-99% | Positive | 20-29% | Negative | [Not Available] | [Not Available] | [Not Evaluated] | Luminal-HER2 neg |
| TCGA-GM-A3XG | Positive | 90-99% | Positive | 90-99% | Negative | [Not Available] | 1+ | Negative | Luminal-HER2 neg |
| TCGA-GM-A3XL | Negative | <10% | Negative | <10% | [Not Evaluated] | [Not Available] | [Not Available] | Negative | Triple negative |
| TCGA-GM-A3XN | Positive | 80-89% | Positive | 90-99% | Negative | [Not Available] | 1+ | Negative | Luminal-HER2 neg |
| TCGA-GM-A4E0 | Positive | 90-99% | Positive | 90-99% | [Not Evaluated] | [Not Available] | [Not Available] | Negative | Luminal-HER2 neg |
| TCGA-GM-A5PV | Positive | 90-99% | Positive | 30-39% | [Not Evaluated] | [Not Available] | [Not Available] | Negative | Luminal-HER2 neg |
| TCGA-GM-A5PX | Positive | 90-99% | Positive | 90-99% | Negative | 30-39% | 1+ | Negative | Luminal-HER2 neg |
| TCGA-HN-A2NL | Negative | [Not Available] | Negative | [Not Available] | Negative | [Not Available] | [Not Available] | [Not Evaluated] | Triple negative |
| TCGA-HN-A2OB | Positive | [Not Available] | Positive | [Not Available] | Negative | [Not Available] | [Not Available] | Negative | Luminal-HER2 neg |
| TCGA-JL-A3YW | Positive | 30-39% | Positive | 30-39% | Positive | 30-39% | 1+ | [Not Evaluated] | Luminal B like (HER2 pos) |
| TCGA-JL-A3YX | Positive | 30-39% | Positive | 30-39% | Positive | 30-39% | 3+ | [Not Evaluated] | Luminal B like (HER2 pos) |
| TCGA-LD-A66U | Positive | 90-99% | Positive | 90-99% | Negative | <10% | [Not Available] | [Not Evaluated] | Luminal-HER2 neg |
| TCGA-LD-A74U | Positive | <10% | Negative | <10% | Negative | [Not Available] | [Not Available] | [Not Evaluated] | Luminal-HER2 neg |
| TCGA-LD-A7W5 | Positive | 90-99% | Positive | [Not Available] | Negative | [Not Available] | [Not Available] | [Not Evaluated] | Luminal-HER2 neg |
| TCGA-LD-A7W6 | Positive | 90-99% | Positive | [Not Available] | Negative | [Not Available] | [Not Available] | [Not Evaluated] | Luminal-HER2 neg |
| TCGA-LD-A9QF | Negative | [Not Available] | Negative | [Not Available] | Positive | 90-99% | 3+ | [Not Evaluated] | HER2-positive (non-luminal) |
| TCGA-LL-A440 | Positive | 90-99% | Positive | 90-99% | Equivocal | [Not Available] | 2+ | Negative | Luminal-HER2 neg |
| TCGA-LL-A441 | Negative | [Not Available] | Negative | [Not Available] | Negative | [Not Available] | 1+ | [Not Evaluated] | Triple negative |
| TCGA-LL-A442 | Positive | 90-99% | Positive | 90-99% | Equivocal | [Not Available] | 2+ | Negative | Luminal-HER2 neg |
| TCGA-LL-A50Y | Positive | 90-99% | Positive | 90-99% | Equivocal | [Not Available] | 2+ | Negative | Luminal-HER2 neg |
| TCGA-LL-A5YL | Positive | 90-99% | Negative | <10% | Positive | [Not Available] | 3+ | Negative | Luminal-HER2 neg |
| TCGA-LL-A5YM | Positive | 90-99% | Positive | 90-99% | Equivocal | [Not Available] | 2+ | Positive | Luminal B like (HER2 pos) |
| TCGA-LL-A5YN | Positive | 90-99% | Positive | 90-99% | Equivocal | [Not Available] | 2+ | Positive | Luminal B like (HER2 pos) |
| TCGA-LL-A5YO | Negative | [Not Available] | Negative | [Not Available] | Negative | [Not Available] | 1+ | [Not Evaluated] | Triple negative |
| TCGA-LL-A5YP | Positive | <10% | Negative | <10% | Negative | <10% | 1+ | Positive | Luminal B like (HER2 pos) |
| TCGA-LL-A6FP | Positive | 90-99% | Positive | 20-29% | Equivocal | [Not Available] | 2+ | Negative | Luminal-HER2 neg |
| TCGA-LL-A6FQ | Positive | 90-99% | Positive | 80-89% | Negative | [Not Available] | 1+ | [Not Evaluated] | Luminal-HER2 neg |
| TCGA-LL-A6FR | Negative | [Not Available] | Positive | <10% | Equivocal | [Not Available] | 2+ | Positive | Undetermined |
| TCGA-LL-A73Y | Negative | [Not Available] | Negative | [Not Available] | Negative | [Not Available] | 3+ | [Not Evaluated] | Triple negative |
| TCGA-LL-A73Z | Positive | 10-19% | Positive | <10% | Equivocal | [Not Available] | 2+ | Negative | Luminal-HER2 neg |
| TCGA-LL-A740 | Negative | [Not Available] | Negative | [Not Available] | Equivocal | [Not Available] | 2+ | Negative | Triple negative |
| TCGA-LL-A7SZ | Positive | 90-99% | Positive | 90-99% | Negative | [Not Available] | 1+ | [Not Evaluated] | Luminal-HER2 neg |
| TCGA-LL-A7T0 | Positive | 90-99% | Positive | <10% | Positive | [Not Available] | 3+ | [Not Evaluated] | Luminal B like (HER2 pos) |
| TCGA-LL-A8F5 | Positive | 20-29% | Negative | <10% | Negative | <10% | 1+ | [Not Evaluated] | Luminal-HER2 neg |
| TCGA-LL-A9Q3 | Positive | 90-99% | Positive | <10% | Positive | [Not Available] | 3+ | [Not Evaluated] | Luminal B like (HER2 pos) |
| TCGA-LQ-A4E4 | Positive | 90-99% | Positive | 90-99% | [Not Evaluated] | [Not Available] | [Not Available] | Negative | Luminal-HER2 neg |
| TCGA-MS-A51U | Positive | 90-99% | Positive | 20-29% | Negative | [Not Available] | [Not Available] | Negative | Luminal-HER2 neg |
| TCGA-OK-A5Q2 | Positive | 90-99% | Positive | 20-29% | Negative | [Not Available] | [Not Available] | [Not Evaluated] | Luminal-HER2 neg |
| TCGA-OL-A5D6 | Negative | [Not Available] | Negative | [Not Available] | [Not Evaluated] | [Not Available] | [Not Available] | Negative | Triple negative |
| TCGA-OL-A5D7 | Negative | [Not Available] | Negative | [Not Available] | [Not Evaluated] | [Not Available] | [Not Available] | Negative | Triple negative |
| TCGA-OL-A5D8 | Positive | 90-99% | Positive | 90-99% | [Not Evaluated] | [Not Available] | [Not Available] | Negative | Luminal-HER2 neg |
| TCGA-OL-A5DA | Positive | 60-69% | Positive | 60-69% | [Not Evaluated] | [Not Available] | [Not Available] | Negative | Luminal-HER2 neg |
| TCGA-OL-A5RU | Positive | 90-99% | Positive | 80-89% | [Not Evaluated] | [Not Available] | [Not Available] | Negative | Luminal-HER2 neg |
| TCGA-OL-A5RV | Positive | 90-99% | Positive | 90-99% | [Not Evaluated] | [Not Available] | [Not Available] | Negative | Luminal-HER2 neg |
| TCGA-OL-A5RW | Negative | [Not Available] | Negative | [Not Available] | [Not Evaluated] | [Not Available] | [Not Available] | Negative | Triple negative |
| TCGA-OL-A5RX | Positive | 90-99% | Positive | 90-99% | [Not Evaluated] | [Not Available] | [Not Available] | Negative | Luminal-HER2 neg |
| TCGA-OL-A5RY | Positive | 10-19% | Negative | [Not Available] | [Not Evaluated] | [Not Available] | [Not Available] | Positive | Luminal B like (HER2 pos) |
| TCGA-OL-A5RZ | Positive | 30-39% | Negative | <10% | [Not Evaluated] | [Not Available] | [Not Available] | Positive | Luminal B like (HER2 pos) |
| TCGA-OL-A5S0 | Positive | 30-39% | Negative | [Not Available] | [Not Evaluated] | [Not Available] | [Not Available] | Positive | Luminal B like (HER2 pos) |
| TCGA-OL-A66H | Positive | 90-99% | Positive | 80-89% | [Not Evaluated] | [Not Available] | [Not Available] | Negative | Luminal-HER2 neg |
| TCGA-OL-A66I | Negative | [Not Available] | Negative | [Not Available] | [Not Evaluated] | [Not Available] | [Not Available] | Negative | Triple negative |
| TCGA-OL-A66J | Positive | 90-99% | Positive | 50-59% | [Not Evaluated] | [Not Available] | [Not Available] | Negative | Luminal-HER2 neg |
| TCGA-OL-A66K | Positive | 70-79% | Positive | 90-99% | Equivocal | [Not Available] | 2+ | Negative | Luminal-HER2 neg |
| TCGA-OL-A66L | Positive | 90-99% | Positive | 90-99% | [Not Evaluated] | [Not Available] | [Not Available] | Negative | Luminal-HER2 neg |
| TCGA-OL-A66N | Positive | 80-89% | Negative | [Not Available] | [Not Evaluated] | [Not Available] | [Not Available] | Negative | Luminal-HER2 neg |
| TCGA-OL-A66O | Positive | 90-99% | Positive | 90-99% | [Not Evaluated] | [Not Available] | [Not Available] | Negative | Luminal-HER2 neg |
| TCGA-OL-A66P | Negative | [Not Available] | Negative | [Not Available] | [Not Evaluated] | [Not Available] | [Not Available] | Negative | Triple negative |
| TCGA-OL-A6VO | Negative | [Not Available] | Negative | [Not Available] | Negative | [Not Available] | [Not Available] | Negative | Triple negative |
| TCGA-OL-A6VQ | Positive | 90-99% | Positive | 10-19% | [Not Evaluated] | [Not Available] | [Not Available] | Negative | Luminal-HER2 neg |
| TCGA-OL-A6VR | Positive | 30-39% | Positive | 90-99% | Negative | [Not Available] | [Not Available] | Negative | Luminal-HER2 neg |
| TCGA-OL-A97C | Negative | [Not Available] | Negative | [Not Available] | [Not Evaluated] | [Not Available] | [Not Available] | Negative | Triple negative |
| TCGA-PE-A5DC | Positive | [Not Available] | Positive | [Not Available] | Positive | [Not Available] | [Not Available] | [Not Evaluated] | Luminal B like (HER2 pos) |
| TCGA-PE-A5DD | Positive | [Not Available] | Negative | [Not Available] | Positive | [Not Available] | [Not Available] | Positive | Luminal B like (HER2 pos) |
| TCGA-PE-A5DE | Positive | [Not Available] | Positive | [Not Available] | Negative | [Not Available] | [Not Available] | [Not Evaluated] | Luminal-HER2 neg |
| TCGA-PL-A8LV | [Not Evaluated] | [Not Available] | [Not Evaluated] | [Not Available] | [Not Evaluated] | [Not Available] | [Not Available] | [Not Evaluated] | Undetermined |
| TCGA-PL-A8LX | [Not Evaluated] | [Not Available] | [Not Evaluated] | [Not Available] | [Not Evaluated] | [Not Available] | [Not Available] | [Not Evaluated] | Undetermined |
| TCGA-PL-A8LY | [Not Evaluated] | [Not Available] | [Not Evaluated] | [Not Available] | [Not Evaluated] | [Not Available] | [Not Available] | [Not Evaluated] | Undetermined |
| TCGA-PL-A8LZ | [Not Evaluated] | [Not Available] | [Not Evaluated] | [Not Available] | [Not Evaluated] | [Not Available] | [Not Available] | [Not Evaluated] | Undetermined |
| TCGA-S3-A6ZF | Positive | 90-99% | Positive | 40-49% | Equivocal | 50-59% | 2+ | Negative | Luminal-HER2 neg |
| TCGA-S3-A6ZG | Positive | 90-99% | Positive | 80-89% | Negative | <10% | [Not Available] | [Not Evaluated] | Luminal-HER2 neg |
| TCGA-S3-A6ZH | Positive | 90-99% | Positive | 80-89% | Equivocal | [Not Available] | 2+ | Negative | Luminal-HER2 neg |
| TCGA-S3-AA0Z | Positive | 90-99% | Positive | <10% | Equivocal | 10-19% | 1+ | Negative | Luminal-HER2 neg |
| TCGA-S3-AA10 | Negative | [Not Available] | Negative | [Not Available] | Negative | [Not Available] | 1+ | [Not Evaluated] | Triple negative |
| TCGA-S3-AA11 | Positive | 90-99% | Positive | 60-69% | Equivocal | <10% | 2+ | Negative | Luminal-HER2 neg |
| TCGA-S3-AA12 | Positive | 90-99% | Negative | [Not Available] | Negative | [Not Available] | 1+ | Negative | Luminal-HER2 neg |
| TCGA-S3-AA14 | Positive | 70-79% | Positive | 70-79% | Positive | 70-79% | 3+ | [Not Evaluated] | Luminal B like (HER2 pos) |
| TCGA-S3-AA15 | Negative | [Not Available] | Negative | [Not Available] | Negative | [Not Available] | [Not Available] | [Not Evaluated] | Triple negative |
| TCGA-S3-AA17 | Positive | 90-99% | Positive | 70-79% | Equivocal | [Not Available] | 2+ | Negative | Luminal-HER2 neg |
| TCGA-UL-AAZ6 | Positive | 90-99% | Positive | <10% | Positive | [Not Available] | [Not Available] | [Not Evaluated] | Luminal B like (HER2 pos) |
| TCGA-UU-A93S | Negative | [Not Available] | Negative | [Not Available] | Positive | 90-99% | 3+ | [Not Evaluated] | HER2-positive (non-luminal) |
| TCGA-W8-A86G | Positive | 90-99% | Positive | 70-79% | Negative | [Not Available] | 1+ | [Not Evaluated] | Luminal-HER2 neg |
| TCGA-WT-AB41 | Positive | 90-99% | Positive | <10% | [Not Evaluated] | [Not Available] | [Not Available] | [Not Evaluated] | Undetermined |
| TCGA-WT-AB44 | Positive | 90-99% | Positive | 90-99% | Negative | [Not Available] | 1+ | [Not Evaluated] | Luminal-HER2 neg |
| TCGA-XX-A899 | Positive | 90-99% | Positive | 70-79% | Negative | [Not Available] | [Not Available] | [Not Evaluated] | Luminal-HER2 neg |
| TCGA-XX-A89A | Positive | 70-79% | Positive | 70-79% | Negative | [Not Available] | [Not Available] | [Not Evaluated] | Luminal-HER2 neg |
| TCGA-Z7-A8R5 | Positive | [Not Available] | Positive | [Not Available] | Negative | [Not Available] | [Not Available] | [Not Evaluated] | Luminal-HER2 neg |
| TCGA-Z7-A8R6 | Positive | [Not Available] | Positive | [Not Available] | Negative | [Not Available] | 1+ | [Not Evaluated] | Luminal-HER2 neg |
